# Gastrointestinal stromal tumors: a case-only analysis of single nucleotide polymorphisms and somatic mutations

**DOI:** 10.1186/2045-3329-3-12

**Published:** 2013-10-26

**Authors:** Katie M O’Brien, Irene Orlow, Cristina R Antonescu, Karla Ballman, Linda McCall, Ronald DeMatteo, Lawrence S Engel

**Affiliations:** 1 Department of Epidemiology, Gillings School of Global Public Health, University of North Carolina, Chapel Hill, NC, USA; 2 Department of Epidemiology and Biostatistics, Memorial Sloan-Kettering Cancer Center, New York, USA; 3 Department of Pathology, Memorial Sloan-Kettering Cancer Center, New York, NY, USA; 4 Department of Health Sciences Research, Mayo Clinic, Rochester, MN, USA; 5 American College of Surgeons Oncology Group, Durham, NC, USA; 6 Department of Surgery, Memorial Sloan-Kettering Cancer Center, New York, NY, USA

**Keywords:** Gastrointestinal stromal tumors, Somatic mutations, Single nucleotide polymorphisms, Epidemiology, Genetics

## Abstract

**Background:**

Gastrointestinal stromal tumors are rare soft tissue sarcomas that typically develop from mesenchymal cells with acquired gain-in-function mutations in *KIT* or *PDGFRA* oncogenes. These somatic mutations have been well-characterized, but little is known about inherited genetic risk factors. Given evidence that certain susceptibility loci and carcinogens are associated with characteristic mutations in other cancers, we hypothesized that these signature *KIT* or *PDGFRA* mutations may be similarly fundamental to understanding gastrointestinal stromal tumor etiology. Therefore, we examined associations between 522 single nucleotide polymorphisms and seven *KIT* or *PDGFRA* tumor mutations types. Candidate pathways included dioxin response, toxin metabolism, matrix metalloproteinase production, and immune and inflammatory response.

**Methods:**

We estimated odds ratios and 95% confidence intervals for associations between each candidate SNP and tumor mutation type in 279 individuals from a clinical trial of adjuvant imatinib mesylate. We used sequence kernel association tests to look for pathway-level associations.

**Results:**

One variant, rs1716 on *ITGAE*, was significantly associated with *KIT* exon 11 non-codon 557–8 deletions (odds ratio = 2.86, 95% confidence interval: 1.71-4.78) after adjustment for multiple comparisons. Other noteworthy associations included rs3024498 (*IL10*) and rs1050783 (*F13A1*) with PDGFRA mutations, rs2071888 (*TAPBP*) with wild type tumors and several matrix metalloproteinase SNPs with *KIT* exon 11 codon 557–558 deletions. Several pathways were strongly associated with somatic mutations in *PDGFRA*, including defense response (p = 0.005) and negative regulation of immune response (p = 0.01).

**Conclusions:**

This exploratory analysis offers novel insights into gastrointestinal stromal tumor etiology and provides a starting point for future studies of genetic and environmental risk factors for the disease.

## Background

Gastrointestinal stromal tumors, or GISTs, are soft tissue sarcomas that develop from mesenchymal connective tissue anywhere in the gastrointestinal tract, though they most frequently appear in the stomach or small intestine [1-3]. Recent advances in molecular biology have revealed a distinct subset of these tumors that express tyrosine kinase receptors or platelet-derived growth factor receptors [4-6]. The presence of these receptors, encoded by the *KIT* or *PDGFRA* oncogenes, respectively, is an indication that these tumors share a common origin of the interstitial cells of Cajal, the pacemaker cells of the gut. The Cajal cells normally express both CD117, the immunohistochemical (IHC) marker for tyrosine kinase receptors, and CD34, the IHC marker for platelet-derived growth factor receptors, and GISTs likely develop from Cajal cells with acquired gain-in-function *KIT* or *PDGFRA* mutations [4,7,8]. *KIT* exon 11 (50-60% of cases) and *KIT* exon 9 (5-10% of cases) are the most common mutation sites [2,9,10]. Such mutations can enable receptor activation in the absence of normal stem cell factor signaling mechanisms, thereby over-stimulating cell proliferation and leading to tumor development. Based on this discovery, a 2001 National Institutes of Health consensus panel [8] agreed to formally define GISTs as mesenchymal neoplasms of the gastrointestinal tract displaying positivity for CD117 or CD34, with some exceptions allowed for immunonegative tumors with otherwise consistent histology.

These relatively new and complex diagnostic criteria make disease surveillance and etiologic study difficult. In a recent evaluation of patients in the National Cancer Institute’s Surveillance, Epidemiology and End Results database, Rubin et al. [11] estimated an annual age-adjusted incidence rate of 0.32 cases per 100,000 individuals in the United States (US). The rarity of the disease makes it a difficult subject for population-based research or for prompt and unbiased assessment of non-genetic risk factors in any study population. An evaluation of the genetic determinants of GIST is much more feasible, as the germline DNA of individuals does not change over time or in response to disease processes. To date, no other research groups have published such evaluations.

To help fill this knowledge gap, we investigated the genetic determinants of GIST in a case-only analysis. Specifically, we examined the associations between select single nucleotide polymorphisms (SNPs), which are inherited variations in individuals’ DNA, and the several common types of acquired *KIT* and *PDGFRA* mutations present in GIST tissue. Certain susceptibility loci have been linked to characteristic mutations, or mutational “signatures”, in other cancers. These include associations between *GSTM1*-null genotype and *TP53* transversion mutations among bladder cancer patients [12], and certain functional polymorphisms in *XPD* and G:C→T:A *TP53* mutations among lung cancer patients [13]. Similarly, we hypothesized that the characteristic somatic mutations in the *KIT* and *PDGFRA* genes in GIST tumors may be mutational signatures that are causally linked to specific mutagens or susceptibility loci. As such, identifying risk factors for the individual tumor subtypes may be fundamental to understanding the disease.

We conducted our evaluation in two phases. The first phase included genes previously linked to soft tissue sarcoma or to environmental risk factors for soft tissue sarcoma, such as dioxins, phenoxyherbicides, insecticides, and vinyl chloride [14-18], as well as genes previously linked to mutational signatures in other cancers [12,13,19-21]. We found that several SNPs were associated with GIST tumor subtypes, including SNPs on two xenobiotic metabolizing genes, *CYP1B1* and *GSTM1*, and two DNA repair genes, *RAD23B* and *ERCC2*[22].

The present report includes results from the second phase of the study, in which we examined the relationship between these 7 somatic mutation categories and 522 additional candidate SNPs. These SNPs are located on genes that play secondary or less well-understood roles in dioxin response or toxin-metabolizing pathways, as well as SNPs on *PDGFRA* and 10 matrix metalloproteinase (*MMP*) genes, which are often over-expressed in GISTs and other soft tissue sarcomas and may be linked to tumor invasion and metastasis [23-25]. Based on previous evidence that immune and inflammation-related genes are associated with osteosarcomas and other gastrointestinal tumors [26-28], we also selected certain SNPs from the GeneChip® Human Immune and Inflammation SNP Kit genotyping panel designed by Affymetrix® [29,30].

Our main objective for these analyses was to identify genes or gene pathways potentially related to GIST carcinogenesis. Therefore, in addition to assessing the effect of each individual SNP, we also assessed the joint effects of SNPs in the same functional categories. By conducting these exploratory analyses with a large and diverse gene panel, our goal is to identify specific variants, genes, or functional pathways meriting further investigation.

## Methods

### Study population

The study population consists of the first 279 individuals from the American College of Surgeons Oncology Group (ACOSOG) Z9001 clinical trial who provided blood and tumor tissue samples for ancillary research and had sufficient tumor tissue for mutation analysis. The Z9001 trial was a multicenter, Phase III, randomized, double-blind study of adjuvant imatinib (Gleevec™; Novartis Pharmaceuticals) versus placebo. To be eligible for the clinical trial, cases had to have a CD117+, resected, localized, primary GIST of at least 3 cm diagnosed between July 1, 2002 and April 18, 2007. IHC staining for CD117 was completed using the Dako antibody (DakoCytomation, Copenhagen, Denmark). Additional information on the Z9001 trial is published elsewhere [31]. Institutional Review Boards at all participating institutions approved this ancillary study and all of the included participants consented to the use of their blood and tissue specimens.

### SNP selection

Among the target genes described above, we identified SNPs within 2000 or 500 base pairs of the 5′ and 3′ ends of the coding regions, respectively, that could affect gene function and had at least a 10% minor allele frequency in the HapMap CEU population [32]. This included nonsense, missense and splice site mutations, as well as SNPs overlapping with microRNA seed regions or transcription binding sites. If the selected SNPs did not meet design phase quality control standards (designability score <1 or final score <0.7), we selected a surrogate SNP in high linkage disequilibrium with the desired SNP. SNPs related to dioxin response or toxin metabolism (final n = 68), matrix metalloproteinase (final n = 24), or on *PDGFRA* (final n = 4) were selected in this manner.

We selected the remaining SNPs (final n = 426) from a pre-existing Affymetrix® panel designed to include potentially functional non-synonymous SNPs from 318 genes related to immune and inflammatory response. Genes were selected for inclusion based on their gene ontology (GO) categorization [33]. GO categories were also used to separate the genes into functional subgroups (see Additional file 1: Table S1).

### Lab assays

DNA for mutation analysis was extracted from snap-frozen tumor tissue and tested for *KIT* exon 11 mutations using polymerase chain reaction (PCR) analysis (Platinum TaqDNA Polymerase High Fidelity; Life Technologies, Inc., Gaithersburg, MD). The PCR conditions were as follows: 1) 94°C for 4 min; 2) 94°C for 30 sec, 3) the relevant annealing temperature for each primer set for 30 sec, 4) 72°C for 30 sec, (35 cycles); and 5) 72°C for 3 min. The PCR products were identified by agarose gel electrophoresis using a 2% MetaPhor™ agarose gel (BioWhittaker Applications, Rockland, ME). The PCR products were purified with the QIAquick™ PCR Purification Kit (Qiagen Inc., Valencia, CA) before sequencing. The sequencing reactions for each case were performed from both the forward and reverse directions. Tumors lacking exon 11 mutations were genotyped for mutations in *KIT* exons 9, 13, 14 and 17 and *PDGFRA* exons 12 and 18. A more detailed description of these assays can be found elsewhere [34,35].

An initial 544 candidate SNPs were genotyped using the GoldenGate genotyping assay (Illumina Inc., San Diego, CA). Briefly, allele-specific oligos were hybridized directly to genomic DNA extracted from the blood samples. The hybridized DNA was extended and ligated to downstream locus-specific oligos and then amplified using universal PCR fluorescently labeled primers and allele-specific primers [30,36]. After the resulting products were hybridized to their complementary bead types, the arrays were assessed using the BeadArray™ Reader.

Twenty-seven participants underwent duplicate genotype analysis for quality assurance purposes. Concordance for duplicate samples was 99.9%. After excluding SNPs that were mono-allelic (n = 3), had >5% missing data (n = 3), or showed poor clustering (n = 16) among our study subjects, we had 522 evaluable SNPs, as listed above. We retained three SNPs that showed evidence of possible copy number variation, but designated them accordingly.

### Statistical analysis

We conducted descriptive analyses of selected demographic variables, tumor characteristics and genotypes. As this population includes some non-white participants, we calculated race-specific MAFs and compared genotype distributions across racial groups (white vs. other) using a Pearson *χ*^2^ test of association. Fisher’s exact test was used if one or more cells had less than 5 observations.

We categorized each individual’s tumor based on the presence or absence of each of the following outcomes: i) a deletion of *KIT* exon 11 codons 557–558, ii) any other (i.e. non-codon 557–8) deletion in *KIT* exon 11, iii) a *KIT* exon 11 insertion, iv) a *KIT* exon 11 point mutation, v) a *KIT* exon 9, exon 13, exon 14, or exon 17 mutation, vi) a *PDGFRA* exon 12 or 18 mutation, and vii) no *KIT* or *PDGFRA* mutation (wild type). KIT mutations in exons 9, 13, 14 and 17 were too rare for independent evaluation.

We obtained odds ratios (ORs), 95% confidence intervals (CIs) and p-values for each SNP-mutation combination using logistic regression. All models were adjusted for race, sex, and age at diagnosis. We assumed additive genetic models, denoting whether an individual had 0, 1 or 2 copies of the minor allele. P-values were calculated using trend tests and were corrected for multiple testing by controlling for a false discovery rate of 25%. This method is less conservative than a Bonferroni approach and is thus better suited for a hypothesis-generating study such as this [37].

We used a sequence kernel association test (SKAT) to assess the joint effect of a group of SNPs on an outcome [38,39]. We grouped SNPs according to their functional category, as described above. This method is well-powered to detect associations when SNPs in a group are correlated with one another but only moderately associated with the outcome. Briefly, for individual *i*, the log odds of having the outcome given genotypes *z*_i1_ to *z*_ip_ and covariates *x*_i1_ to *x*_im_ is modeled semi-parametrically using a logistic kernel-machine regression model:

$$\begin{array}{ll} \text{logit}\;P\left(y_{i}=1\right)= & \alpha_{0}+\alpha_{1}x_{i1}+\cdots +\alpha_{m}x_{\mathrm{im}}\\ & +h\left(z_{i1},z_{i2},\ldots ,z_{\mathrm{ip}}\right), \end{array}$$

where *h*(***Z***_**i**_) is a function of a positive, definite kernel function, K(•,•) and some γ_i_, …, γ_n_:

$$h\left(Z_{i}\right)=\sum_{i=1}^{n}\gamma_{i'}K\left(Z_{i},Z_{i'}\right)$$

We used an identity-by-state kernel: $K\left(Z_{i},Z_{i'}\right)=\sum_{j=1}^{p}\left(2‒\left|Z_{\mathrm{ij}}‒Z_{i'j}\right|\right)$, as this does not require linearity assumptions and allows for epistasis [39]. Assuming **h** follows an arbitrary distribution with a mean of 0 and variance τ**K**, testing the null hypothesis H_0_: h(**Z**) = 0 is equivalent to testing H_0_: τ=0. This is accomplished using a modified variance-component score statistic:

$$Q=\frac{\left(y‒{\hat{p}}_{0}\right)'K\left(y‒{\hat{p}}_{0}\right)}{2},$$

where logit

$${\hat{p}}_{0i}={\hat{a}}_{0}+{\hat{a}}_{1}x_{i1}+{\hat{a}}_{2}x_{i2}+\cdots +{\hat{a}}_{m}x_{\mathrm{im}}\text{.}$$

Here, *Q* is comparable to a *χ*^2^ distribution with scale parameter κ and ν degrees of freedom, both of which are modified to account for correlation between SNPs in the same SNP-set (for calculations, see Appendix A in Wu et al. [38]).

## Results

Males and females were approximately equally represented in our study population, while 18% were non-white (Table 1). Median age at diagnosis was 58 years (range 18–85), though non-white participants tended to be younger (median age = 53 years). Non-white participants were also more likely to have a smaller tumor than white participants (median tumor sizes of 6.0 cm versus 6.5 cm), and more likely to have stomach tumors (74% versus 64%). Most patients had mutations in *KIT* exon 11 (70% overall), the largest proportion of which were codon 557–558 deletions (34% of exon 11 mutations). Demographic and tumor characteristics were very similar for males and females.

**Table 1:** Demographic information and tumor characteristics of patients included in genotyping ancillary study

	**Overall sample**	**Sex stratified**		**Race stratified**	
	**N = 279**	**Male (n = 142)**	**Female (n = 137)**	**White (n = 229)**	**Other (n = 50)**
**Age: Median (range)**	58.0 (18 – 85)	57.0 (18 – 85)	58.0 (18 – 81)	59.0 (18 – 85)	53.0 (27 – 78)
**Sex: N (%)**					
Male	142 (51)	---	---	---	---
Female	137 (49)	---	---	---	---
**Race: N (%)**					
White	229 (82)	122 (86)	107 (78)	---	---
Other	50 (18)	20 (14)	30 (22)	---	---
**Tumor Size: Median (range)**	6.5 (3.0 – 37.0)	6.0 (3.0 – 37.0)	6.5 (3.0 – 28.0)	6.5 (3.0 – 37.0)	6.0 (3.1 – 30.0)
**Tumor Size: N(%)**					
<5 cm	79 (28)	41 (29)	38 (28)	65 (28)	14 (28)
5-10 cm	146 (52)	72 (51)	74 (54)	119 (52)	27 (54)
>10 cm	54 (19)	29 (20)	25 (18)	45 (20)	9 (18)
**Mitotic Rate: Median (range)**	3 (0 – 351)	3 (0 – 351)	3 (0 – 207)	3 (0 – 351)	4.5 (0 – 81)
**Mitotic Rate: N(%)**					
<5	156 (60)	77 (58)	79 (63)	132 (62)	24 (50)
≥5	104 (40)	57 (42)	47 (37)	80 (38)	24 (50)
Missing	19	8	11	17	2
**Tumor Location: N(%)**					
Stomach	182 (66)	97 (69)	85 (63)	146 (64)	36 (74)
Small Intestine	85 (31)	39 (28)	46 (34)	77 (34)	8 (16)
Rectum	2 (1)	1 (1)	1 (1)	1 (0)	1 (2)
Other	8 (3)	4 (3)	4 (3)	4 (2)	4 (8)
Missing	2	1	1	1	1
**Mutation Type: N(%)**					
Exon 9	15 (5)	9 (6)	6 (4)	15 (7)	0 (0)
Exon 11	195 (70)	95 (67)	100 (73)	153 (67)	42 (84)
Exon 13	3 (1)	0 (0)	3 (2)	2 (1)	1 (2)
Exon 14	1 (0)	1 (1)	0 (0)	1 (0)	0 (0)
Exon 17	0 (0)	0 (0)	0 (0)	0 (0)	0 (0)
PDGFRA	29 (10)	21 (15)	8 (6)	25 (11)	4 (8)
Wild type	36 (13)	16 (11)	20 (15)	33 (14)	3 (6)
**Exon 11 mutation type: N(%)**					
557-558 deletion	66 (34)	33 (35)	33 (33)	51 (33)	15 (36)
Other deletion	45 (23)	25 (26)	20 (20)	34 (22)	11 (26)
Insertion	28 (14)	14 (15)	14 (14)	23 (15)	5 (12)
Point Mutation	56 (29)	23 (24)	33 (33)	45 (29)	11 (26)
**PDGFRA mutation type: N(%)**					
D842V	12 (41)	10 (48)	2 (25)	10 (40)	2 (50)
Other	17 (59)	11 (52)	6 (75)	15 (60)	2 (50)

Compared with the larger ACOSOG Z9001 cohort, whites were somewhat over-represented in this ancillary study, which had otherwise similar characteristics (data not shown). Race-stratified MAF and association p-values for all 522 SNPs are displayed in Additional file 1: Table S2. As expected based on genotype distributions in ethnically diverse HapMap populations [32], genotype distributions in this study differed by race for many of the candidate polymorphisms.

The top 5 SNPs for each mutation subtype are displayed in Table 2. Only one SNP, rs1716 on *ITGAE*, was statistically significant after adjusting for multiple comparisons. This SNP was associated with *KIT* exon 11 non-codon 557–8 deletions (OR = 2.86, 95% CI: 1.71, 4.78; p = 6.4×10^-5^).

**Table 2:** Odds ratios (ORs) for top 5 SNP-mutation associations, by mutation type

**KIT exon 11 codon 557–558 deletion**	**Gene**	MMP10	MMP1	MMP10	SELP	GRN
	**SNP**	rs3819099	rs17293642	rs17293348	rs6131	rs5848
	**MAF**^**a**^	0.11/0.20	0.11/0.20	0.12/0.20	0.18/0.30	0.31/0.45
	**OR (95% CI)**	2.29 (1.30, 4.01)	2.17 (1.25, 3.77)	2.16 (1.24, 3.77)	1.81 (1.18, 2.78)	1.78 (1.17, 2.73)
	**p-value**	0.004	0.006	0.007	0.007	0.007
**KIT exon 11 other (non-codon 557–8) deletion**	**Gene**	ITGAE	CDK2	FCER1G	ZAP70	LIF
	**SNP**	rs1716	rs2069398	rs11421	rs2276645	rs737812
	**MAF**^**a**^	0.25/0.45	0.07/0.19	0.15/0.27	0.40/0.22	0.35/0.20
	**OR (95% CI)**	2.86 (1.71, 4.78)	2.62 (1.34, 5.13)	2.13 (1.24, 3.64)	0.49 (0.28, 0.83)	0.47 (0.26, 0.83)
	**p-value**	0.00006	0.005	0.006	0.008	0.01
**KIT exon 11 insertion**	**Gene**	SH2B3	CLCF1	GZMB	IFI16	GMIP
	**SNP**	rs3184504	rs17608	rs8192917	rs866484	rs880090
	**MAF**^**a**^	0.43/0.23	0.32/0.48	0.24/0.41	0.26/0.44	0.32/0.48
	**OR (95% CI)**	0.31 (0.15, 0.64)	2.55 (1.38, 4.72)	2.33 (1.28, 4.24)	2.36 (1.27, 4.40)	2.20 (1.23, 3.94)
	**p-value**	0.002	0.003	0.006	0.007	0.008
**KIT exon 11 point mutation**	**Gene**	MMP7	PTGER3	PTPN12	MMP7	SLAMF1
	**SNP**	rs10502001	rs959	rs3750050	rs14983	rs2295612
	**MAF**^**a**^	0.18/0.29	0.18/0.31	0.16/0.29	0.19/0.29	0.17/0.07
	**OR (95% CI)**	2.20 (1.32, 3.66)	1.99 (1.25, 3.17)	2.01 (1.25, 3.25)	2.03 (1.22, 3.39)	0.33 (0.15, 0.74)
	**p-value**	0.003	0.004	0.004	0.006	0.007
**Other KIT mutation**	**Gene**	LILRA4	LAG3	IL4R	MMP1	ITGAE
	**SNP**	rs2241384	rs870849	rs1805015	rs4754880	rs1716
	**MAF**^**a**^	0.17/0.36	0.36/0.42	0.18/0.36	0.18/0.36	0.27/0.50
	**OR (95% CI)**	2.89 (1.36, 6.16)	2.76 (1.33, 5.74)	2.75 (1.32, 5.75)	2.49 (1.22, 5.09)	2.46 (1.22, 4.99)
	**p-value**	0.006	0.007	0.007	0.01	0.01
**PDGFRA mutation**	**Gene**	IL10	F13A1	PLAU	PECAM1	SPINK5
	**SNP**	rs3024498	rs1050783	rs4065	rs1050382	rs6892205
	**MAF**^**a**^	0.21/0.41	0.14/0.33	0.46/0.24	0.47/0.31	0.49/0.31
	**OR (95% CI)**	0.31 (0.16, 0.60)	0.31 (0.16, 0.61)	2.65 (1.37, 5.13)	0.43 (0.24, 0.78)	2.31 (1.26, 4.24)
	**p-value**	0.0004	0.0007	0.004	0.006	0.007
**Wild type**	**Gene**	TAPBP	ESR1	NCF2	STAT2	FGA
	**SNP**	rs2071888	rs6557171	rs2274064	rs2066807	rs6050
	**MAF**^**a**^	0.48/0.29	0.38/0.46	0.43/0.38	0.06 /0.15	0.26/0.39
	**OR (95% CI)**	0.37 (0.20, 0.67)	2.26 (1.35, 3.79)	2.35 (1.34, 4.13)	3.00 (1.32, 6.83)	2.02 (1.19, 3.44)
	**p-value**	0.001	0.002	0.003	0.009	0.009

^a^Minor allele frequency (MAF) among those without mutation / MAF among those with mutation.

**Figure 1:**
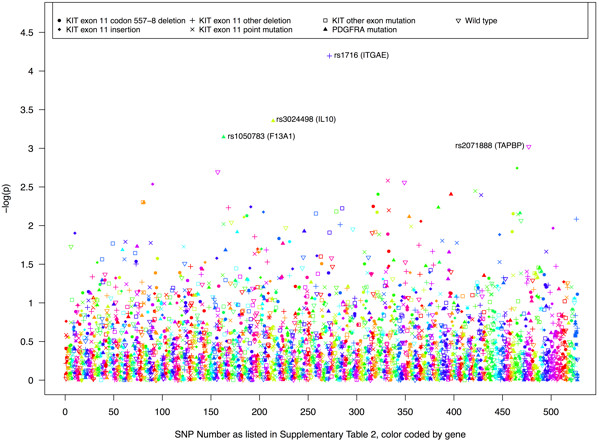
Log p-values for the association between each candidate SNP and tumor mutation type.

Though not statistically significant after adjusting for multiple comparisons, rs3024498 (*IL10*) and rs1050783 (*F13A1*) were strongly associated with *PDGFRA* mutations (OR = 0.31, 95% CI: 0.16,0.60 and OR = 0.31, 95% CI: 0.16,0.61, respectively) and rs2071888 (*TAPBP*) was strongly associated with wild type tumors (OR = 0.37, 95% CI: 0.20, 0.67). Additionally, several SNPs in matrix metalloproteinase genes were associated with tumor subtypes. *MMP10* and *MMP1* SNPs were associated with *KIT* exon 11 codon 557–558 deletions, 2 *MMP7* SNPs were associated with *KIT* exon 11 point mutations and a *MMP1* SNP was associated with *KIT* exon 9, 13, 14, or 17 mutations. Effect estimates and p-values for all 522 SNPs can be found in Additional file 1: Table S3. The relative magnitude of all SNP-subtype associations is depicted in Figure 1.

**Figure 2:**
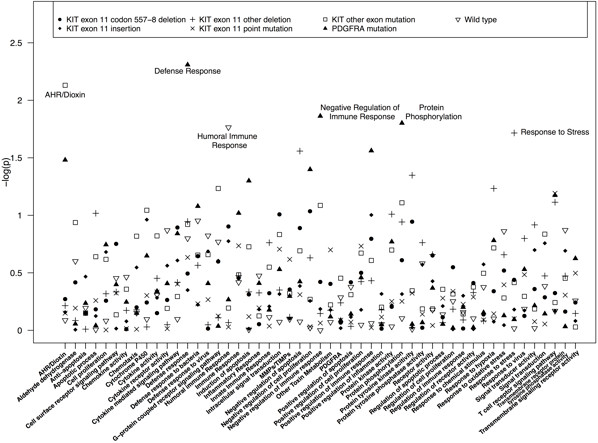
SKAT log p-values for the association between each functional pathway and tumor mutation type.

Despite strong SNP-level effects, the *MMP* pathway was not associated with any of the tumor subtypes in the SKAT analyses (minimum p-value =0.2 for *KIT* exon 11 point mutations; Additional file 1: Table S4). As seen in Table 3, the strongest pathway-level associations were in relation to somatic mutations in *PDGFRA*. This included defense response (p = 0.005), negative regulation of immune response (p = 0.01), protein phosphorylation (p = 0.02), positive regulation of immune response (p = 0.03), and AHR/dioxin response (0.03). Additionally, negative regulation of cell proliferation was associated with *PDGFRA* mutations (p = 0.04; Additional file 1: Table S4). In total, only 5 other pathways were associated with a tumor subtype at p < 0.05. These were AHR/dioxin response with non exon 11 *KIT* mutations (p = 0.01), humoral immune response with wild type mutations (p = 0.02), and response to stress, negative regulation of apoptosis, and protein tyrosine kinase activity with non-codon 557–8 *KIT* exon 11 deletions (p = 0.02, p = 0.03, and p = 0.04, respectively). No pathways were statistically significant after correcting for multiple testing. Log p-values for all pathway analyses can be seen in Figure 2.

**Table 3:** SKAT p-values for top 5 functional pathway- mutation associations, by mutation type

**KIT exon 11 codon 557–558 deletion**	**Pathway**	Negative regulation of cell proliferation	Intracellular signal transduction	Protein tyrosine kinase phosphatase activity	Humoral immune response	Cytokine mediated signaling pathway
	**p-value**	0.09	0.10	0.11	0.13	0.13
**KIT exon 11 other (non-codon 557–8) deletion**	**Pathway**	Response to stress	Negative regulation of apoptosis	Protein tyrosine kinase activity	Response to Hypoxia	T cell receptor signaling pathway
	**p-value**	0.02	0.03	0.04	0.06	0.08
**KIT exon 11 insertion**	**Pathway**	Positive regulation of immune response	Cytochrome P450	Humoral immune response	Signal transduction	Signal transducer activity
	**p-value**	0.10	0.11	0.17	0.17	0.20
**KIT exon 11 point mutation**	**Pathway**	T cell receptor signaling pathway	Immune response	Positive regulation of cell proliferation	Intracellular signal transduction	Phase I/II metabolizing
	**p-value**	0.06	0.18	0.19	0.20	0.20
**Other KIT mutation**	**Pathway**	AHR/dioxin response	G-protein coupled receptor signaling pathway	Protein phosphorylation	Negative regulation of immune response	Cytochrome P450
	**p-value**	0.01	0.06	0.08	0.08	0.09
**PDGFRA mutation**	**Pathway**	Defense Response	Negative regulation of immune response	Protein phosphorylation	Positive regulation of immune response	AHR/dioxin response
	**p-value**	0.005	0.01	0.02	0.03	0.03
**Wild type**	**Pathway**	Humoral immune response	Defense response to bacteria	Transmembrane receptor protein tyrosine phosphatase activity	Cytokine receptor activity	Response to oxidative stress
	**p-value**	0.02	0.11	0.13	0.14	0.14

## Discussion

In this exploratory analysis of genetic risk factors for GIST tumor subtypes, we identified one statistically significant association and a number of other potentially important associations for individual polymorphisms. We also identified several potentially relevant functional pathways. These novel findings offer clues about the etiology of these rare and poorly understood tumors.

The SNP with the strongest association with a tumor subtype was rs1716 on *ITGAE.* This SNP results in a missense mutation on *ITGAE* (also known as *CD103*), a gene involved in protein tyrosine phosphatase activity. This SNP was previously associated with increased risk of melanoma [40], as was another SNP in the gene. The CD103 protein is commonly expressed in intraepithelial lymphocytic T cells and hairy cell leukemia cells [41,42].

The *IL10* SNP associated with *PDGFRA* mutations, rs3024498, is located in a seed microRNA region. One previous study found an association between rs3024498 and colorectal cancer [43]. The *IL10* gene encodes a cytokine that plays a role in immunoregulation and inflammation, and has been previously linked to several cancers, including osteosarcoma [26], cervical cancer [44], and gastric cancer [45,46].

rs1050783 in *F13A1* is also in a seed microRNA region, but neither the SNP nor the gene has been previously linked to cancer. The same is true for rs2071888 in *TAPBP*, a missense mutation. As noted above, several studies have observed over-expression of matrix metalloproteinase genes in GISTs and other soft tissue sarcomas [23-25], though none of the evaluated SNPs have previously been associated with cancer risk.

Pathway analyses are common in cancer epidemiology, but there is little consistency in how the pathways are defined. We selected the well-documented and publicly available Gene Ontology [33] classification system to facilitate replication and follow-up studies. Although we did not identify any studies that examined the specific pathways included in the present analyses, numerous studies have observed associations of inflammatory or immune response genes with risk of sarcomas or gastrointestinal cancers [26,28,30,47].

The present exploratory study was undertaken in light of evidence suggesting that some mutagens and susceptibility loci are associated with specific mutational “signatures,” i.e., characteristic mutation patterns [12,13,19-21]. At this point, GIST etiology has been insufficiently researched, and we do not know how well the mutation-based tumor classifications used in this study correspond to distinct carcinogenic processes. However, the existence of multiple types of mutations suggest that more than one mutagenic process could be involved, and we believe that identifying associations between germline genetic polymorphisms and unique tumor phenotypes could contribute valuable new information about disease etiology. This information could also help to elucidate environmental risk factors for this disease.

Because tumors with *KIT* exon 11 mutations were not assessed for other *KIT* or *PDGFRA* mutations, some tumors may be misclassified, though evidence from population-based studies suggests that few GISTs have more than one mutation type [2,9]. Our study participants had similar mutation profiles to the individuals included in these population-based investigations, but the results from this predominately white clinical trials population may not be generalizable to all GIST patients. Lastly, this study had a small sample size. As such, we had limited power to detect true associations, particularly when the evaluated genotype and mutation type were rare.

## Conclusions

In this novel study of genetic risk factors for GIST, we identified several SNPs and gene pathways associated with GIST mutation subtypes. This included SNPs involved in dioxin response, toxin metabolism, matrix metalloproteinase synthesis, and inflammatory or immune response. While only a single SNP was statistically significant after correcting for multiple comparisons, our overall findings provide an important starting point for future studies of genetic and environmental risk factors for this rare and poorly understood disease.

## Abbreviations

GISTs: Gastrointestinal stromal tumors; SNPs: Single nucleotide polymorphisms; SKAT: Sequence kernel association test; OR: Odds ratio; CI: Confidence interval; US: United States; ACOSOG: American college of surgeons oncology group; GO: Gene ontology; PCR: Polymerase chain reaction; MAF: Minor allele frequency.

## Competing interests

This work was supported by Novartis Pharmaceuticals Corp. agreement #CSTI571BUS249. RD previously served as a paid consultant to Novartis Pharmaceuticals. All other authors declare that they have no conflict of interest.

## Authors’ contributions

LSE, IO, CRA and RD conceived the study design, selected the candidate genes, and supervised genetic analyses. KMO and LSE designed the statistical analysis and drafted the manuscript. KMO and LM preformed the statistical analyses, with contributions from LSE and KB. All authors read and approved the final manuscript.

## Supplementary Material

Additional file 1: Table S1 Genes and gene pathways. **Table S2.** Minor allele frequencies (MAF) and p-values for comparison of genotype frequencies: Z9001 genotyped whites (n=273) versus non-whites (n=58). **Table S3.** Odds Ratios (ORs), 95% Confidence Intervals (Cis) and p-values for the association between candidate SNPs and tumor mutation status in 279 GIST patients. **Table S4.** P-values for sequence kernel association tests (SKAT) of functional pathways and tumor mutation status. Click here for file
