# Supplementary material for: Gastrointestinal stromal tumors: a case-only analysis of single nucleotide polymorphisms and somatic mutations
Source: Clin Sarcoma Res. 2013 Oct 26;3:12. doi: 10.1186/2045-3329-3-12 (PMC3827940; doi:10.1186/2045-3329-3-12)
Supplement: Additional file 1: Table S1 — Genes and gene pathways. Table S2. Minor allele frequencies (MAF) and p-values for comparison of genotype frequencies: Z9001 genotyped whites (n=273) versus non-whites (n=58). Table S3. Odds Ratios (ORs), 95% Confidence Intervals (Cis) and p-values for the association between candidate SNPs and tumor mutation status in 279 GIST patients. Table S4. P-values for sequence kernel association tests (SKAT) of functional pathways and tumor mutation status. [file 2045-3329-3-12-S1.docx]

| **Supplementary Table 1: Genes and gene pathways** | | | |
| --- | --- | --- | --- |
| **Pathway Description** | **Number of SNPs in Pathway** | **Number of Genes in Pathway** | **Genes in Pathway** |
| **CANDIDATE GENES** | | | |
| **AHR and dioxin response** | 21 | 10 | CCNE1, CDK2, EGFR, ESR1, FASLG, FOS, SERPINB2, TGFB1, TGFB3, VEGFA |
| **Alcohol dehydrogenase (toxin metabolism)** | 4 | 2 | ALDH3B1, ALDH3B2 |
| **Cytochrome P450 (dioxin response)** | 31 | 12 | CYP11A1, CYP17A1, CYP19A1, CYP24A1, CYP2A7, CYP2B6, CYP2C19, CYP2U1, CYP4B1, CYP4F2, CYP4F3, CYP8B1 |
| **Other toxin metabolism** | 12 | 4 | GSTM3, MTHFR, PTGIS, SULT2A1 |
| **MMPs** | 24 | 10 | MMP1, MMP10, MMP12, MMP14, MMP16, MMP2, MMP3, MMP7, MMP8, MMP9 |
| **PDGFRA** | 4 | 1 | PDGFRA |
| **INFLAMMATORY AND IMMUNE RESPONSE GENES (Defined using GO ontology categories)** | | | |
| **Anti-apoptosis** | 21 | 18 | BAG1, BAG3, BCL2A1, BIRC5, CD27, CDKN2D, DAPK1, FAS, HSP90B1, IGF1, IGF1R, IL10, IL1A, IL1B, IL31RA, MATL1, MYD88, PRKCZ |
| **Apoptotic process** | 65 | 49 | ABL1, BAG1, BAG3, BCL10, BCL2A1, BCL2L14, BIRC5, BIRC8, CARD6, CASP1, CASP10, CASP2, CASP5, CASP7, CASP8, CASP9, CHIA, DAP, DAPK1, EDAR, FAM3B, FAS, GZMB, IL19, IL1A, IL1B, IL24, INPP5D, ITGB2, LITAF, LTBR, PDCD5, PDCD6IP, PIM1, PPP1R15A, PPP2R2B, PPP3R1, PRKCB, PRKCQ, PTEN, PTK2B, STK17A, TGFBR1, TNFRSF10B, TNFRSF11B, TNFRSF19, TRAF1, TRAF3, WDR92 |
| **Cell proliferation** | 25 | 16 | CD274, CD74, CSF1, CUL5, IFI16, IL15RA, IL1A, IL5RA, IRF2, LIPA, PIM1, PTEN, STAT3, SYK, TGFB2, THPO |
| **Cell surface receptor signaling pathway** | 31 | 25 | AGER, BAG1, C5, CBLB, CD101, CD27, CD274, CD36, CD97, CLCF1, CXCL10, IFNAR1, IFNGR2, IL17RA, IL18RAP, IL7R, INHBA, LAG3, LIFR, LILRB3, MERTK, MYD88, PTK2B, TNFRSF10B, TNFRSF14 |
| **Chemokine activity** | 7 | 6 | C5, CCL16, CCL26, CXCL10, CXCL16, CXCL9 |
| **Chemotaxis** | 18 | 13 | C5, CCL16, CCL26, CCR8, CMTM6, CX3CR1, CXCL10, CXCL16, CXCL9, DOCK2, FPR1, LECT2, PLAU |
| **Cytokine activity** | 33 | 29 | CLCF1, CMTM6, CSF1, CXCL9, FAM3B, GRN, IL10, IL11, IL12A, IL12B, IL13, IL16, IL19, IL1A, IL1B, IL1F10, IL24, IL25, IL29, IL3, IL36A, IL36G, IL36RN, IL37, INHBA, LIF, TGFB2, THPO, TNFRSF11B |
| **Cytokine receptor activity** | 2 | 13 | CD74, FLT3, GHR, IL11RA, IL12B, IL15RA, IL1RL1, IL23R, IL28RA, IL31RA, IL4R, IL5RA, IL7R |
| **Cytokine-mediated signaling pathway** | 39 | 29 | ADAR, CD44, CD74, CIITA, CLCF1, FLT3, HLA-DMA, HLA-DPA1, HLA-DRA, IFI35, IFNAR1, IFNGR1, IFNGR2, IL15RA, IL1A, IL1B, IL1RL1, IL28RA, IRF2, IRF3, IRF8, LIFR, MYD88, OAS1, OAS2, STAT2, STAT3, STAT6, ZNF675 |
| **Defense response** | 14 | 11 | CD74, CD84, CXCL9, DARC, GATA3, IL31RA, INHBA, LILRB3, MALT1, NOD2, TAPBP |
| **Defense response to bacterium** | 14 | 12 | BPI, FCER1G, IL10, IRF8, LTF, LYST, MICA, NOD2, SYK, TLR1, TLR3, TLR6 |
| **Defense response to virus** | 17 | 15 | CD207, CD40, CD8A, CXCL10, CXCL9, IFNAR1, IL12B, IL29, LILRB1, LYST, MICA, OAS1, TLR3, TLR7, TLR8 |
| **G-protein coupled receptor signaling pathway** | 17 | 14 | C5, CCR8, CD97, CX3CR1, CXCL9, DARC, ENTPD1, FPR1, GPR15, LPAR2, MLN, PTGER3, S1PR2, S1PR3 |
| **Humoral immune response** | 3 | 3 | BST1, LTF, PAX5 |
| **Immune response** | 80 | 57 | BPI, CBLB, CCL16, CCL26, CCR8, CD274, CD74, CD8A, CD97, CHIA, CIITA, CR2, CXCL10, CXCL9, ETS1, FAS, FCAR, FCGR2B, FYB, HLA-DMA, HLA-DPA1, HLA-DRA, IFI35, IGF1R, IGLL1, IL10RB, IL12A, IL13, IL16, IL18R1, IL18RAP, IL19, IL1A, IL1RL1, IL3, IL36A, IL37, IL4R, IL7R, IRF8, KIR2DS2, LIF, LST1, LY75, MICA, OAS1, OAS2, PDCD1LG2, PTGER4, RAG1, TAP2, TAPBP, TLR1, TLR10, TLR6, TNFRSF14, ZAP70 |
| **Induction of apoptosis** | 31 | 22 | AIFM2, BCL10, BCL2L13, C5, C6, CASP10, CASP7, CD27, DAPK1, ETS1, FAS, IL19, INHBA, PDCD5, PRCKE, PTEN, STK17A, TGFB1, TGFBR1, TNFRSF10A, TNFRSF19, TRAF3 |
| **Inflammatory response** | 61 | 45 | AGER, AIF1, APOL3, C5, CCL16, CCL26, CD40, CD97, CSF1, CXCL10, CXCL9, DARC, F11R, HDAC4, IL10, IL10RB, IL13, IL18RAP, IL1A, IL1B, IL23R, IL36A, ITGB2, LIPA, LY75, MYD88, NFATC3, NFATC4, NMI, PLA2G2D, PRKCQ, PRKCZ, S1PR3, SELE, SELP, TGFB1, TICAM2, TLR1, TLR10, TLR3, TLR5, TLR6, TLR7, TLR8, TNFAIP6 |
| **Innate immune response** | 54 | 43 | AGER, BCL10, C4BPA, C4BPB, C5, C6, CASP1, CASP10, CASP8, CD209, CFH, CLEC4M, CR2, CSF1, DUSP6, HSP90B1, IFIH1, IL18R1, IL18RAP, IL1RAPL2, IL1RL1, IRF3, LYN, MALT1, MAPK9, MASP2, MBL2, MYD88, NCF2, NOD2, PGLYRP2, PGLYRP3, SARM1, SYK, TICAM2, TLR1, TLR10, TLR3, TLR5, TLR6, TLR7, TLR8, TRAF3 |
| **Intracellular signal transduction** | 22 | 16 | CBLB, CD209, CLEC4M, DLG5, GMIP, INPP5D, LYN, NFATC1, NOD2, PRKCB, PRKCE, PRKCQ, PRKCZ, SH2B3, TEC, TRAF3IP2 |
| **Negative regulation of apoptotic process** | 20 | 16 | ADAR, AIF1, CD44, CD74, CLCF1, CX3CR1, DNAJA3, IGF1R, INSL3, PIK3R1, PIM1, PTEN, PTK2B, SERPINE1, TDGF1, TGFBR1 |
| **Negative regulation of cell proliferation** | 38 | 26 | CDKN2D, CUL5, CYP27B1, DLG5, DNAJA3, ETS1, FLT3, GATA3, HDAC4, IGF1, IGFBP3, IL1A, IL1B, IL29, INHBA, INSL3, LIF, LYN, PTEN, PTK2B, PTPRJ, PTPRK, PTPRU, TGFB1, TGFB2 |
| **Negative regulation of immune response** | 11 | 5 | INPP5D, LYN, SPINK5, TGFB1, TGFB2 |
| **Positive regulation of apoptotic process** | 20 | 17 | ABL1, ACE, AGER, CASP2, DNAJA3, DUSP6, FAS, IGFBP3, IL1B, INPP5D, LILRB1, NFATC4, PIAS4, PIK3R1, STK17A, TGFB2, TLR3 |
| **Positive regulation of cell proliferation** | 41 | 30 | BIRC5, CDK2, CLCF1, CSF1, CXCL10, DPP4, EGF, FLT3, HDAC4, IGF1, IGF1R, IL11, IL3, IL31RA, INSL3, LIF, LIFR, LYN, PDF, PTEN, PTK2B, S1PR2, S1PR3, SLAM1, TDGF1, TGFB1, TGFB2, TGFBR1, THPO, TNFRSF11A |
| **Positive regulation of immune response** | 4 | 3 | HLA-DMA, IL29, TGFB2 |
| **Positive regulation of inflammatory response** | 10 | 9 | ACE, AGER, IL12B, IL1RL1, MAPK13, PTGER4, SERPINE1, TLR3, TLR7 |
| **Protein kinase activity** | 15 | 12 | AKT3,CDK2, DAPK1, MAPK13, MAPK9, PRKCE, PRKCZ, PTK2B, SYK, TEC, TGFBR1, TXK |
| **Protein phosphorylation** | 38 | 25 | AKT3, BIRC5, CDK2, DAPK1, FYB, IGFBP3, LYN, MAP3K6, MERTK, PIK3R1, PIM1, PRKCB, PRKCE, PRKCQ, PRKCZ, PTK2B, PTPRE, STK17A, SYK, TEC, TGFB1, TGFB2, TGFRB1, TXK, ZAP70 |
| **Protein tyrosine kinase activity** | 11 | 8 | ABL1, IGF1R, LYN, MERTK, PTK2B, TESK1, TXK, XAP70 |
| **Protein tyrosine phosphatase activity** | 27 | 17 | DUSP10, DUSP2, DUSP6, PTEN, PTPN12, PTPN13, PTPN22, PTPRB, PTPRD, PTPRE, PTPGR, PTPRJ, PTPRK, PTPRR, PTPRT, PTPRU, PTPRZ1 |
| **Receptor activity** | 91 | 67 | ABL1, AGER, CD209, CD226, CD274, CD40, CD84, CHRNA2, CLEC4M, CR2, CUL5, DARC, DPP4, EDAR, FAS, FCAR, FCGR2B, FPR1, HAVCR1, IFNGR1, IFNGR2, IL10RA, IL10RB, IL15RA, IL18R1, IL18RAP, IL20RA, IL20RB, IL28RA, IL5RA, IL7R, ITGAE, ITGB2, ITGB3, KLRK1, LILRA4, LILRB3, LILRB4, LTBR, LY75, LY9, MERTK, MMD, PDCD1LG2, PILRB, PLXNC1, PPARGC1B, PTCRA, PTPN12, PTPN22, PTPRD, PTPRE, PTPRJ, PTPRK, PTPRT, SLAMF1, TLR1, TLR3, TLR5, TLR6, TLR7, TLR8, TNFRSF10A, TNFRSF10B, TNFRSF11A, TNFRSF11B, TNFRSF14 |
| **Regulation of apoptotic process** | 29 | 24 | BCL2A1, BCL2L13, BCL2L14, BIRC5, CARD14, CARD6, CASP1, CASP10, CASP2, CASP5, CASP8, CASP9, DAPK1, DLG5, DUSP2, FAS, FLT3, IFIH1, LYN, MALT1, TGFBR1, TNFRSF10B, TRAF1, TRAF3 |
| **Regulation of cell proliferation** | 6 | 4 | BAG6, FAS, PLAU, SERPINE1 |
| **Regulation of immune response** | 12 | 11 | CD226, CD247, CD40, CD8A, FCGR2B, ITGB2, KIR2DS2, KLRK1, LILRB1, MICA, PVRL2 |
| **Regulation of receptor activity** | 4 | 2 | PLAU, SERPINE1 |
| **Response to chemical stimulus** | 3 | 2 | BCL10, TLR7 |
| **Response to hypoxia** | 25 | 15 | ACE, DPP4, FAS, HSP90B1, IL1A, IL1B, NOS1, NR4A2, PLAU, PPARA, PRKCQ, PTK2B, TGFB1, TGFB2, TGFBR1, |
| **Response to oxidative stress** | 3 | 2 | GPX6, MBL2 |
| **Response to stress** | 10 | 8 | BAG1, C5, DUSP10, HSP90B1, MAPK13, MAPK9, PTK2B, TRAP1 |
| **Response to virus** | 17 | 14 | GATA3, IFI16, IFI35, IFIH1, IFNAR1, IFNGR1, IFNGR2, IL12A, IRF3, LILRB1 |
| **Signal transducer activity** | 25 | 18 | AHRR, APOL3, CBLB, CD40, FAS, IL10RA, IL11RA, IL15RA, LITAF, MALT1, PLRG1, PRKCE, PTK2B, SH2B3, STAT2, STAT3, TICAM2, TRAF3 |
| **Signal transduction** | 116 | 81 | AKT3, ALCAM, APOL3, CASP1, CBLB, CCL26, CD226, CD274, CD40, CD74, CHRNA2, CXCL10, CXCL9, DAPK1, DLG5, EDAR, EDARADD, EGF, FAS, FCGR2B, FGA, FGB, FPR1, FYB, GATA3, GRN, IFNGR1, IGF1, IGF1R, IL10RA, IL10RB, IL11RA, IL13, IL15RA, IL18R1, IL19, IL1B, IL1RL1, IL4R, IL5RA, IL7R, INPP5D, KLRK1, LILRB1, LILRB4, LITAF, LTBR, LYN, MAP3K6, MYD88, NFAT5, NR4A2, PECAM1, PIK3R1, PLAU, PLRG1, PLXNC1, PPIC, PPP2R2B, PRKCB, PRKCE, PRKCZ, PTK2B, PTPRK, PTPRT, SARM1, STAT3, TDGF1, TGFBR1, TICAM2, TLR1, TLR3, TLR6, TLR8, TNFAIP6, TNFRSF10A, TNFRSF10B, TNFRSF11A, TNFRSF11B, TRAF1, TRAF3 |
| **T cell receptor signaling pathway** | 23 | 15 | BCL10, CD247, FYB, GATA3, HLA-DMA, HLA-DPA1, HLA-DRA, INPP5D, MALT1, PIK3R1, PRKCQ, PTEN, PTPN22, TXK, ZAP70 |
| **Transmembrane receptor protein tyrosine phosphatase activity** | 13 | 8 | PTPRB, PTPRD, PTPRE, PTPRG, PTPRK, PTPRR, PTPRU, PTPRZ1 |
| **Transmembrane signaling receptor activity** | 25 | 22 | AGER, CD247, CD27, CD44, CD97, CR2, DARC, EDAR, FAS, IL11RA, IL18RAP, IL1RAPL2, KIR2DS2, LAG3, LILRB3, SELE, SLAMF1, TLR1, TLR10, TLR3, TLR6, TNFRSF11A |

| **Supplementary Table 2: Minor allele frequencies (MAF) and p-values for comparison of genotype frequencies: Z9001 genotyped whites (n=273) versus non-whites (n=58)** | | | | | |
| --- | --- | --- | --- | --- | --- |
| **SNP Number** | **Gene** | **Variant** | **MAF, study whites** | **MAF, study non-whites** | **P-value whites vs. non-whites** |
| 1 | ABL1 | rs2987902 | 0.16 | 0.34 | 2.5E-05 |
| 2 | ABL1 | rs3824400 | 0.14 | 0.40 | 3.3E-11 |
| 3 | ACE | rs4343 | 0.47 | 0.27 | 1.4E-06 |
| 4 | ACTR8 | rs2241806 | 0.32 | 0.44 | 0.05 |
| 5 | ADAR | rs2229857 | 0.28 | 0.47 | 3.3E-05 |
| 6 | ADAR | rs3738032 | 0.19 | 0.17 | 0.86 |
| 7 | AGER | rs2288419 | 0.21 | 0.37 | 1.2E-04 |
| 8 | AHRR | rs2672725 | 0.13 | 0.21 | 0.06 |
| 9 | AIF1 | rs2269475 | 0.13 | 0.10 | 0.85 |
| 10 | AIFM2 | rs7893137 | 0.11 | 0.24 | 0.002 |
| 11 | AKT3 | rs3006927 | 0.16 | 0.15 | 0.27 |
| 12 | ALCAM | rs1044240 | 0.16 | 0.07 | 0.06 |
| 13 | ALCAM | rs627925 | 0.25 | 0.29 | 0.48 |
| 14 | ALCAM | rs7648171 | 0.24 | 0.37 | 0.02 |
| 15 | ALDH3B1 | rs15518 | 0.24 | 0.35 | 0.05 |
| 16 | ALDH3B1 | rs2286169 | 0.24 | 0.41 | 0.001 |
| 17 | ALDH3B1 | rs581105 | 0.49 | 0.45 | 0.28 |
| 18 | ALDH3B2 | rs1551886 | 0.09 | 0.26 | 1.8E-05 |
| 19 | APOL3 | rs132653 | 0.17 | 0.37 | 1.1E-08 |
| 20 | ATP6V1G2 | rs2071594 | 0.30 | 0.49 | 1.7E-04 |
| 21 | B3GNT3 | rs36686 | 0.21 | 0.26 | 0.06 |
| 22 | BAG1 | rs706115 | 0.18 | 0.32 | 0.01 |
| 23 | BAG3 | rs8946 | 0.34 | 0.38 | 0.52 |
| 24 | BAG6 | rs1046089 | 0.31 | 0.47 | 6.3E-06 |
| 25 | BCL10 | rs2735592 | 0.30 | 0.23 | 0.05 |
| 26 | BCL10 | rs962409 | 0.49 | 0.42 | 0.05 |
| 27 | BCL2A1 | rs1138357 | 0.26 | 0.29 | 0.66 |
| 28 | BCL2A1 | rs1138358 | 0.26 | 0.48 | 2.6E-07 |
| 29 | BCL2L13 | rs2535704 | 0.14 | 0.03 | 0.01 |
| 30 | BCL2L13 | rs9306198 | 0.10 | 0.01 | 0.002 |
| 31 | BCL2L14 | rs1797647 | 0.27 | 0.18 | 0.06 |
| 32 | BCL2L14 | rs885720 | 0.21 | 0.44 | 1.1E-07 |
| 33 | BIRC5 | rs1042489 | 0.39 | 0.42 | 0.71 |
| 34 | BIRC8 | rs8109165 | 0.43 | 0.33 | 0.11 |
| 35 | BPI | rs1341022 | 0.44 | 0.34 | 2.6E-05 |
| 36 | BPI | rs4358188 | 0.48 | 0.38 | 0.11 |
| 37 | BRSK1 | rs2286721 | 0.16 | 0.16 | 0.74 |
| 38 | BRSK1 | rs2288523 | 0.14 | 0.28 | 0.001 |
| 39 | BRSK1 | rs2532500 | 0.32 | 0.38 | 0.43 |
| 40 | BST1 | rs2302465 | 0.14 | 0.03 | 0.002 |
| 41 | C4BPA | rs4844573 | 0.37 | 0.41 | 1.1E-04 |
| 42 | C4BPB | rs6690037 | 0.49 | 0.27 | 1.1E-07 |
| 43 | C5 | rs17611 | 0.44 | 0.19 | 1.6E-06 |
| 44 | C6 | rs1801033 | 0.36 | 0.49 | 0.04 |
| 45 | CAPN2 | rs10961 | 0.38 | 0.28 | 1.0E-11 |
| 46 | CARD14 | rs755340 | 0.22 | 0.24 | 0.10 |
| 47 | CARD6 | rs10512747 | 0.14 | 0.03 | 0.001 |
| 48 | CASP1 | rs2282659 | 0.26 | 0.22 | 0.48 |
| 49 | CASP10 | rs13006529 | 0.49 | 0.31 | 5.8E-06 |
| 50 | CASP2 | rs3181166 | 0.15 | 0.04 | 0.01 |
| 51 | CASP5 | rs507879 | 0.44 | 0.48 | 0.31 |
| 52 | CASP5 | rs523104 | 0.44 | 0.49 | 0.62 |
| 53 | CASP7 | rs4353229 | 0.28 | 0.17 | 0.08 |
| 54 | CASP8 | rs3769823 | 0.32 | 0.46 | 4.6E-04 |
| 55 | CASP9 | rs2020902 | 0.14 | 0.07 | 0.11 |
| 56 | CBLB | rs7649466 | 0.16 | 0.18 | 0.19 |
| 57 | CBLB | rs894541 | 0.12 | 0.19 | 0.06 |
| 58 | CCL16 | rs2063979 | 0.30 | 0.24 | 2.4E-18 |
| 59 | CCL26 | rs2302009 | 0.25 | 0.33 | 0.27 |
| 60 | CCNE1 | rs1406 | 0.20 | 0.36 | 0.001 |
| 61 | CCR8 | rs2853699 | 0.32 | 0.10 | 8.8E-06 |
| 62 | CD101 | rs3754112 | 0.33 | 0.07 | 3.3E-08 |
| 63 | CD109 | rs5023688 | 0.32 | 0.40 | 0.28 |
| 64 | CD207 | rs17718987 | 0.29 | 0.04 | 1.7E-10 |
| 65 | CD207 | rs741326 | 0.44 | 0.45 | 0.09 |
| 66 | CD209 | rs8105483 | 0.16 | 0.12 | 0.44 |
| 67 | CD226 | rs763361 | 0.49 | 0.38 | 0.05 |
| 68 | CD247 | rs1052231 | 0.18 | 0.24 | 0.21 |
| 69 | CD27 | rs25680 | 0.23 | 0.17 | 0.45 |
| 70 | CD274 | rs2297136 | 0.44 | 0.32 | 0.08 |
| 71 | CD36 | rs7755 | 0.45 | 0.30 | 0.02 |
| 72 | CD40 | rs1569723 | 0.27 | 0.22 | 0.001 |
| 73 | CD44 | rs1467558 | 0.18 | 0.02 | 9.7E-07 |
| 74 | CD44 | rs353612 | 0.25 | 0.26 | 0.95 |
| 75 | CD74 | rs15251 | 0.27 | 0.09 | 8.5E-05 |
| 76 | CD84 | rs3733264 | 0.30 | 0.16 | 0.003 |
| 77 | CD8A | rs3020729 | 0.18 | 0.16 | 0.89 |
| 78 | CD97 | rs2230748 | 0.14 | 0.16 | 0.13 |
| 79 | CDK2 | rs2069391 | 0.06 | 0.09 | 0.13 |
| 80 | CDK2 | rs2069398 | 0.06 | 0.24 | 1.3E-07 |
| 81 | CDKN2D | rs12984043 | 0.41 | 0.16 | 6.2E-06 |
| 82 | CFH | rs1065489 | 0.16 | 0.14 | 0.95 |
| 83 | CFH | rs800292 | 0.25 | 0.35 | 1.1E-16 |
| 84 | CHIA | rs2256721 | 0.28 | 0.27 | 0.95 |
| 85 | CHIA | rs2275254 | 0.40 | 0.37 | 3.9E-05 |
| 86 | CHIA | rs3818822 | 0.10 | 0.16 | 0.13 |
| 87 | CHRNA2 | rs2280375 | 0.15 | 0.18 | 0.51 |
| 88 | CIITA | rs3087519 | 0.50 | 0.32 | 0.001 |
| 89 | CLCF1 | rs17608 | 0.34 | 0.38 | 0.61 |
| 90 | CLEC4M | rs475896 | 0.46 | 0.28 | 2.6E-05 |
| 91 | CMTM6 | rs4796 | 0.38 | 0.46 | 0.003 |
| 92 | CR2 | rs17615 | 0.31 | 0.28 | 0.14 |
| 93 | CR2 | rs4308977 | 0.31 | 0.41 | 0.02 |
| 94 | CR2 | rs6540433 | 0.15 | 0.02 | 4.2E-05 |
| 95 | CSF1 | rs333947 | 0.18 | 0.07 | 0.01 |
| 96 | CUL5 | rs15677 | 0.22 | 0.33 | 0.05 |
| 97 | CX3CR1 | rs3732378 | 0.17 | 0.06 | 0.01 |
| 98 | CXCL10 | rs1063499 | 0.40 | 0.30 | 1.2E-08 |
| 99 | CXCL10 | rs13157656 | 0.23 | 0.10 | 0.003 |
| 100 | CXCL16 | rs1050998 | 0.47 | 0.32 | 0.01 |
| 101 | CXCL9 | rs3733236 | 0.07 | 0.25 | 1.3E-07 |
| 102 | CYP11A1 | rs11635047 | 0.44 | 0.15 | 6.1E-15 |
| 103 | CYP17A1 | rs6162 | 0.47 | 0.41 | 0.11 |
| 104 | CYP19A1 | rs12148604 | 0.48 | 0.45 | 0.79 |
| 105 | CYP19A1 | rs12591359 | 0.45 | 0.39 | 0.44 |
| 106 | CYP19A1 | rs6493487 | 0.27 | 0.13 | 0.001 |
| 107 | CYP19A1 | rs700518 | 0.46 | 0.30 | 0.01 |
| 108 | CYP19A1 | rs934634 | 0.24 | 0.28 | 0.39 |
| 109 | CYP24A1 | rs2296239 | 0.20 | 0.47 | 1.1E-09 |
| 110 | CYP24A1 | rs4809957 | 0.20 | 0.47 | 1.0E-09 |
| 111 | CYP24A1 | rs4809958 | 0.15 | 0.13 | 0.12 |
| 112 | CYP24A1 | rs6068816 | 0.11 | 0.11 | 0.02 |
| 113 | CYP27B1 | rs1048691 | 0.24 | 0.36 | 0.01 |
| 114 | CYP2A7 | rs10419393 | 0.48 | 0.47 | 0.94 |
| 115 | CYP2A7 | rs12461727 | 0.17 | 0.13 | 0.39 |
| 116 | CYP2A7 | rs3869579 | 0.49 | 0.35 | 0.04 |
| 117 | CYP2A7 | rs4803397 | 0.27 | 0.23 | 0.60 |
| 118 | CYP2B6 | rs1042389 | 0.19 | 0.32 | 0.01 |
| 119 | CYP2B6 | rs2054675 | 0.22 | 0.34 | 0.01 |
| 120 | CYP2B6 | rs2099361 | 0.38 | 0.11 | 4.7E-08 |
| 121 | CYP2B6 | rs8100458 | 0.34 | 0.28 | 0.11 |
| 122 | CYP2B6 | rs8192719 | 0.22 | 0.35 | 0.002 |
| 123 | CYP2C19 | rs1042194 | 0.16 | 0.18 | 0.30 |
| 124 | CYP2C19 | rs2281891 | 0.16 | 0.16 | 0.12 |
| 125 | CYP2U1 | rs8727 | 0.26 | 0.14 | 0.02 |
| 126 | CYP4B1 | rs2297809 | 0.13 | 0.15 | 0.04 |
| 127 | CYP4B1 | rs4646487 | 0.12 | 0.21 | 0.03 |
| 128 | CYP4B1 | rs837398 | 0.19 | 0.41 | 1.1E-06 |
| 129 | CYP4F2 | rs1272 | 0.23 | 0.25 | 0.29 |
| 130 | CYP4F3 | rs1140855 | 0.18 | 0.09 | 0.04 |
| 131 | CYP8B1 | rs12494055 | 0.47 | 0.28 | 0.001 |
| 132 | CYP8B1 | rs2228468 | 0.37 | 0.40 | 0.79 |
| 133 | CYP8B1 | rs3732860 | 0.47 | 0.28 | 0.001 |
| 134 | DAP | rs5484 | 0.19 | 0.14 | 2.8E-04 |
| 135 | DAP | rs9857 | 0.23 | 0.49 | 3.5E-08 |
| 136 | DAPK1 | rs1056719 | 0.37 | 0.47 | 0.03 |
| 137 | DARC | rs12075 | 0.42 | 0.32 | 7.5E-06 |
| 138 | DLG5 | rs1248696 | 0.10 | 0.04 | 0.07 |
| 139 | DNAJA3 | rs4785963 | 0.28 | 0.39 | 0.06 |
| 140 | DOCK2 | rs3734099 | 0.12 | 0.24 | 0.002 |
| 141 | DPP4 | rs3788979 | 0.11 | 0.15 | 0.11 |
| 142 | DUSP10 | rs6670514 | 0.27 | 0.35 | 0.21 |
| 143 | DUSP2 | rs1724120 | 0.46 | 0.47 | 0.17 |
| 144 | DUSP6 | rs770087 | 0.18 | 0.28 | 0.02 |
| 145 | EDAR | rs5021634 | 0.14 | 0.30 | 3.9E-28 |
| 146 | EDARADD | rs966365 | 0.16 | 0.33 | 3.1E-04 |
| 147 | EGF | rs2237051 | 0.38 | 0.26 | 5.1E-13 |
| 148 | EGF | rs4698803 | 0.20 | 0.05 | 0.001 |
| 149 | EGFR | rs10228436 | 0.36 | 0.27 | 0.09 |
| 150 | EGFR | rs10277413 | 0.37 | 0.27 | 0.09 |
| 151 | EGFR | rs4947982^a^ | 0.45 | 0.39 | 0.02 |
| 152 | EGFR | rs887826 | 0.15 | 0.07 | 0.10 |
| 153 | ENTPD1 | rs3181115 | 0.10 | 0.03 | 0.06 |
| 154 | ESR1 | rs3020371 | 0.33 | 0.41 | 1.9E-06 |
| 155 | ESR1 | rs3798577 | 0.46 | 0.44 | 0.91 |
| 156 | ESR1 | rs6557171 | 0.36 | 0.49 | 0.01 |
| 157 | ESR1 | rs974276 | 0.12 | 0.41 | 1.4E-12 |
| 158 | ETS1 | rs4372467 | 0.39 | 0.47 | 0.25 |
| 159 | ETS1 | rs4937333 | 0.45 | 0.39 | 0.005 |
| 160 | ETS1 | rs8705 | 0.32 | 0.09 | 2.5E-08 |
| 161 | F11R | rs1062827 | 0.29 | 0.14 | 0.002 |
| 162 | F13A1 | rs1050783 | 0.17 | 0.13 | 0.58 |
| 163 | FAM3B | rs417708 | 0.14 | 0.10 | 0.74 |
| 164 | FAS | rs2234978 | 0.28 | 0.25 | 0.83 |
| 165 | FASLG | rs10458360 | 0.49 | 0.23 | 5.6E-10 |
| 166 | FCAR | rs16986050 | 0.16 | 0.19 | 0.11 |
| 167 | FCER1G | rs11421 | 0.17 | 0.13 | 0.35 |
| 168 | FCGR2B | rs1801274 | 0.46 | 0.44 | 0.86 |
| 169 | FGA | rs2070022 | 0.17 | 0.16 | 0.90 |
| 170 | FGA | rs6050 | 0.26 | 0.32 | 0.48 |
| 171 | FGB | rs4220 | 0.20 | 0.15 | 0.45 |
| 172 | FLT3 | rs1933437 | 0.41 | 0.47 | 0.08 |
| 173 | FOS | rs1569328 | 0.16 | 0.04 | 0.002 |
| 174 | FPR1 | rs2070745 | 0.35 | 0.39 | 0.14 |
| 175 | FPR1 | rs5030878 | 0.22 | 0.16 | 0.15 |
| 176 | FPR1 | rs867228 | 0.21 | 0.22 | 0.16 |
| 177 | FYB | rs358501 | 0.20 | 0.23 | 0.64 |
| 178 | FYB | rs379707 | 0.33 | 0.31 | 4.5E-13 |
| 179 | GATA3 | rs1058240 | 0.21 | 0.23 | 0.11 |
| 180 | GATA3 | rs3802604 | 0.36 | 0.35 | 1.6E-08 |
| 181 | GBP7 | rs676913 | 0.34 | 0.22 | 0.05 |
| 182 | GHR | rs6180 | 0.45 | 0.47 | 0.81 |
| 183 | GMIP | rs880090 | 0.30 | 0.39 | 0.19 |
| 184 | GPR15 | rs2230344 | 0.14 | 0.13 | 0.55 |
| 185 | GPX6 | rs406113 | 0.32 | 0.34 | 7.2E-11 |
| 186 | GRN | rs5848 | 0.29 | 0.49 | 1.7E-05 |
| 187 | GSTM3 | rs3814309 | 0.31 | 0.34 | 0.001 |
| 188 | GSTM3 | rs7483 | 0.30 | 0.34 | 2.4E-04 |
| 189 | GZMB | rs2236338 | 0.23 | 0.32 | 0.02 |
| 190 | GZMB | rs8192917 | 0.23 | 0.41 | 3.8E-04 |
| 191 | HAVCR1 | rs1553316 | 0.16 | 0.11 | 0.10 |
| 192 | HDAC4 | rs1962113 | 0.33 | 0.22 | 0.06 |
| 193 | HDAC4 | rs870790 | 0.34 | 0.38 | 0.65 |
| 194 | HLA-DMA | rs1063478 | 0.12 | 0.21 | 0.03 |
| 195 | HLA-DPA1 | rs1042190 | 0.18 | 0.48 | 2.2E-11 |
| 196 | HLA-DPA1 | rs3077 | 0.18 | 0.50 | 4.8E-12 |
| 197 | HLA-DRA | rs7192 | 0.38 | 0.37 | 0.79 |
| 198 | HLA-DRA | rs8084 | 0.41 | 0.40 | 0.95 |
| 199 | HSP90B1 | rs1882019 | 0.09 | 0.04 | 0.01 |
| 200 | HSP90B1 | rs2164747 | 0.13 | 0.35 | 1.0E-07 |
| 201 | IFI16 | rs1057027 | 0.18 | 0.46 | 1.7E-13 |
| 202 | IFI16 | rs6940 | 0.14 | 0.26 | 0.01 |
| 203 | IFI16 | rs866484 | 0.22 | 0.48 | 6.3E-09 |
| 204 | IFI35 | rs10840 | 0.08 | 0.16 | 0.01 |
| 205 | IFIH1 | rs3747517 | 0.26 | 0.39 | 0.03 |
| 206 | IFNAR1 | rs2834202 | 0.28 | 0.16 | 0.01 |
| 207 | IFNGR1 | rs3799488 | 0.12 | 0.11 | 0.12 |
| 208 | IFNGR2 | rs9808753 | 0.14 | 0.27 | 0.001 |
| 209 | IGF1 | rs6214 | 0.38 | 0.46 | 0.01 |
| 210 | IGF1R | rs8030950 | 0.28 | 0.40 | 0.04 |
| 211 | IGFBP3 | rs6670 | 0.20 | 0.09 | 0.02 |
| 212 | IGLL1 | rs131429 | 0.39 | 0.35 | 0.07 |
| 213 | IL10 | rs3024498 | 0.27 | 0.09 | 3.2E-05 |
| 214 | IL10RA | rs2512143 | 0.35 | 0.31 | 0.10 |
| 215 | IL10RB | rs2834167 | 0.23 | 0.28 | 0.24 |
| 216 | IL11 | rs1042506 | 0.13 | 0.06 | 0.004 |
| 217 | IL11RA | rs3808868 | 0.49 | 0.34 | 0.002 |
| 218 | IL12A | rs583911 | 0.46 | 0.29 | 0.001 |
| 219 | IL12B | rs2546890 | 0.48 | 0.35 | 0.001 |
| 220 | IL13 | rs20541 | 0.23 | 0.23 | 0.58 |
| 221 | IL15RA | rs2228059 | 0.47 | 0.29 | 1.7E-06 |
| 222 | IL15RA | rs2296135 | 0.50 | 0.29 | 4.6E-06 |
| 223 | IL15RA | rs3136618 | 0.46 | 0.34 | 3.8E-05 |
| 224 | IL16 | rs1131445 | 0.32 | 0.30 | 0.89 |
| 225 | IL16 | rs4072111 | 0.13 | 0.12 | 1.00 |
| 226 | IL17RA | rs5992628 | 0.41 | 0.49 | 0.15 |
| 227 | IL18R1 | rs3732127 | 0.15 | 0.34 | 1.6E-05 |
| 228 | IL18RAP | rs1420106 | 0.27 | 0.16 | 0.02 |
| 229 | IL19 | rs2243191 | 0.23 | 0.40 | 0.002 |
| 230 | IL1A | rs17561 | 0.27 | 0.18 | 0.09 |
| 231 | IL1B | rs1143627 | 0.31 | 0.43 | 1.9E-06 |
| 232 | IL1F10 | rs2100071 | 0.32 | 0.05 | 1.3E-09 |
| 233 | IL1RAPL2 | rs3764765 | 0.11 | 0.28 | 6.1E-06 |
| 234 | IL1RL1 | rs1041973 | 0.22 | 0.41 | 5.5E-05 |
| 235 | IL1RL1 | rs3771175 | 0.12 | 0.28 | 1.1E-04 |
| 236 | IL20RA | rs1342642 | 0.28 | 0.22 | 0.02 |
| 237 | IL20RB | rs108858 | 0.44 | 0.33 | 0.01 |
| 238 | IL22RA1 | rs16829204 | 0.18 | 0.16 | 0.80 |
| 239 | IL22RA1 | rs3795299 | 0.37 | 0.43 | 0.43 |
| 240 | IL22RA1 | rs3795300 | 0.40 | 0.21 | 1.5E-04 |
| 241 | IL22RA2 | rs202566 | 0.21 | 0.02 | 3.6E-07 |
| 242 | IL23R | rs1004819 | 0.32 | 0.30 | 0.82 |
| 243 | IL23R | rs1358748 | 0.14 | 0.27 | 0.002 |
| 244 | IL23R | rs1884444 | 0.48 | 0.49 | 0.76 |
| 245 | IL23R | rs7530511 | 0.14 | 0.23 | 0.02 |
| 246 | IL24 | rs1150253 | 0.44 | 0.31 | 0.02 |
| 247 | IL25 | rs8014568 | 0.31 | 0.41 | 1.7E-12 |
| 248 | IL28RA | rs10903034 | 0.45 | 0.44 | 0.08 |
| 249 | IL29 | rs30461 | 0.10 | 0.44 | 7.9E-13 |
| 250 | IL3 | rs40401 | 0.23 | 0.43 | 1.1E-11 |
| 251 | IL31RA | rs161704 | 0.30 | 0.45 | 0.01 |
| 252 | IL36A | rs2305152 | 0.31 | 0.12 | 9.9E-06 |
| 253 | IL36A | rs895497 | 0.29 | 0.19 | 0.07 |
| 254 | IL36G | rs7584409 | 0.30 | 0.11 | 6.3E-05 |
| 255 | IL36RN | rs2515402 | 0.39 | 0.30 | 0.22 |
| 256 | IL37 | rs3811047 | 0.30 | 0.46 | 6.3E-10 |
| 257 | IL4R | rs1805015 | 0.18 | 0.23 | 0.33 |
| 258 | IL4R | rs8832 | 0.44 | 0.41 | 0.03 |
| 259 | IL5RA | rs2290610 | 0.38 | 0.43 | 0.61 |
| 260 | IL7R | rs1494555 | 0.31 | 0.17 | 0.01 |
| 261 | IL7R | rs1494558 | 0.31 | 0.28 | 0.54 |
| 262 | IL7R | rs3194051 | 0.26 | 0.28 | 0.85 |
| 263 | IL7R | rs6897932 | 0.31 | 0.13 | 8.9E-05 |
| 264 | INHBA | rs2237432 | 0.28 | 0.25 | 0.25 |
| 265 | INPP5D | rs9247 | 0.15 | 0.24 | 0.03 |
| 266 | INSL3 | rs1003887 | 0.36 | 0.34 | 0.85 |
| 267 | IRF2 | rs3756103 | 0.46 | 0.36 | 0.13 |
| 268 | IRF2 | rs965225 | 0.11 | 0.03 | 0.005 |
| 269 | IRF3 | rs2304207 | 0.15 | 0.07 | 0.09 |
| 270 | IRF8 | rs10514611 | 0.24 | 0.24 | 0.64 |
| 271 | ITGAE | rs1716 | 0.33 | 0.14 | 2.1E-04 |
| 272 | ITGAE | rs2976230 | 0.21 | 0.42 | 2.3E-05 |
| 273 | ITGB2 | rs2070946 | 0.24 | 0.23 | 0.76 |
| 274 | ITGB3 | rs11079772 | 0.32 | 0.20 | 0.03 |
| 275 | KIR2DS2 | rs2242653 | 0.16 | 0.10 | 0.31 |
| 276 | KLRK1 | rs1841958 | 0.31 | 0.36 | 0.32 |
| 277 | KLRK1 | rs2617170 | 0.32 | 0.46 | 3.9E-05 |
| 278 | LAG3 | rs870849 | 0.35 | 0.45 | 0.03 |
| 279 | LECT2 | rs31517 | 0.38 | 0.35 | 0.86 |
| 280 | LGALS8 | rs1041935 | 0.34 | 0.41 | 5.0E-07 |
| 281 | LGALS8 | rs2243525 | 0.24 | 0.36 | 0.01 |
| 282 | LIF | rs737812 | 0.34 | 0.20 | 0.01 |
| 283 | LIFR | rs3110234 | 0.21 | 0.12 | 0.12 |
| 284 | LILRA4 | rs2241384 | 0.17 | 0.23 | 0.29 |
| 285 | LILRB1 | rs8101605 | 0.17 | 0.15 | 0.63 |
| 286 | LILRB3 | rs448461 | 0.26 | 0.28 | 0.82 |
| 287 | LILRB4 | rs731170 | 0.32 | 0.47 | 0.005 |
| 288 | LIPA | rs1727 | 0.24 | 0.06 | 1.5E-05 |
| 289 | LIPA | rs17468739 | 0.16 | 0.02 | 4.2E-05 |
| 290 | LIPA | rs2070845 | 0.25 | 0.20 | 0.25 |
| 291 | LITAF | rs4280262 | 0.22 | 0.09 | 0.01 |
| 292 | LPAR2 | rs1054685 | 0.37 | 0.34 | 0.39 |
| 293 | LST1 | rs3130062 | 0.08 | 0.01 | 0.01 |
| 294 | LTBR | rs12354 | 0.27 | 0.08 | 5.0E-05 |
| 295 | LTF | rs1126478 | 0.33 | 0.25 | 8.4E-15 |
| 296 | LY75 | rs12692566 | 0.18 | 0.11 | 0.26 |
| 297 | LY75 | rs2666986 | 0.25 | 0.32 | 0.28 |
| 298 | LY9 | rs509749 | 0.41 | 0.26 | 1.1E-10 |
| 299 | LY9 | rs574610 | 0.44 | 0.39 | 4.2E-04 |
| 300 | LYN | rs952784 | 0.21 | 0.19 | 0.57 |
| 301 | LYST | rs3768072 | 0.32 | 0.28 | 0.002 |
| 302 | MALT1 | rs1059442 | 0.10 | 0.34 | 1.3E-08 |
| 303 | MALT1 | rs7506506 | 0.14 | 0.09 | 0.07 |
| 304 | MAP3K6 | rs1138294 | 0.36 | 0.21 | 1.2E-04 |
| 305 | MAPK13 | rs2071863 | 0.17 | 0.23 | 0.11 |
| 306 | MAPK9 | rs9605 | 0.40 | 0.41 | 0.24 |
| 307 | MASP2 | rs1033638 | 0.19 | 0.50 | 6.8E-12 |
| 308 | MBL2 | rs2506 | 0.19 | 0.46 | 4.1E-13 |
| 309 | MBL2 | rs930507 | 0.17 | 0.31 | 0.002 |
| 310 | MERTK | rs13027171 | 0.29 | 0.07 | 3.0E-07 |
| 311 | METTL1 | rs703842 | 0.30 | 0.36 | 0.22 |
| 312 | MICA | rs1051792 | 0.29 | 0.44 | 0.01 |
| 313 | MLN | rs2281820 | 0.41 | 0.39 | 0.78 |
| 314 | MMD | rs2286806 | 0.45 | 0.41 | 0.01 |
| 315 | MMD | rs4476230 | 0.45 | 0.10 | 1.1E-12 |
| 316 | MMP1 | rs17293642 | 0.15 | 0.02 | 1.1E-04 |
| 317 | MMP1 | rs470215^a^ | 0.39 | 0.30 | 0.09 |
| 318 | MMP1 | rs4754880 | 0.18 | 0.22 | 0.49 |
| 319 | MMP1 | rs7112080 | 0.09 | 0.00 | 1.4E-04 |
| 320 | MMP10 | rs17293348 | 0.15 | 0.03 | 3.8E-04 |
| 321 | MMP10 | rs3819099 | 0.15 | 0.02 | 1.2E-04 |
| 322 | MMP12 | rs28381705 | 0.12 | 0.11 | 0.88 |
| 323 | MMP14 | rs3751488 | 0.23 | 0.16 | 0.11 |
| 324 | MMP16 | rs1467251 | 0.18 | 0.31 | 0.01 |
| 325 | MMP16 | rs2664347 | 0.26 | 0.15 | 0.02 |
| 326 | MMP16 | rs2664368 | 0.21 | 0.43 | 3.2E-05 |
| 327 | MMP2 | rs1132896 | 0.38 | 0.13 | 5.2E-06 |
| 328 | MMP2 | rs2241145 | 0.48 | 0.47 | 0.47 |
| 329 | MMP2 | rs243834 | 0.45 | 0.47 | 0.14 |
| 330 | MMP3 | rs679620 | 0.49 | 0.29 | 4.5E-04 |
| 331 | MMP7 | rs10502001 | 0.24 | 0.04 | 2.4E-06 |
| 332 | MMP7 | rs14983 | 0.24 | 0.05 | 1.1E-05 |
| 333 | MMP8 | rs1940475 | 0.49 | 0.32 | 2.6E-04 |
| 334 | MMP8 | rs2701993 | 0.46 | 0.41 | 0.47 |
| 335 | MMP9 | rs17576 | 0.33 | 0.44 | 0.05 |
| 336 | MMP9 | rs20544 | 0.41 | 0.19 | 1.1E-12 |
| 337 | MMP9 | rs2274755 | 0.12 | 0.16 | 0.18 |
| 338 | MMP9 | rs3918249 | 0.33 | 0.42 | 9.8E-06 |
| 339 | MMP9 | rs9509 | 0.03 | 0.28 | 8.0E-17 |
| 340 | MTHFR | rs1537514 | 0.10 | 0.13 | 0.01 |
| 341 | MTHFR | rs1801131 | 0.30 | 0.21 | 0.004 |
| 342 | MTHFR | rs1801133 | 0.37 | 0.08 | 6.6E-10 |
| 343 | MTHFR | rs4846047 | 0.29 | 0.34 | 0.10 |
| 344 | MTHFR | rs4846049 | 0.32 | 0.43 | 0.02 |
| 345 | MTHFR | rs6697244 | 0.38 | 0.48 | 0.004 |
| 346 | MTHFR | rs7538516 | 0.38 | 0.48 | 0.003 |
| 347 | MYD88 | rs7744 | 0.16 | 0.11 | 0.12 |
| 348 | NCF2 | rs2274064 | 0.47 | 0.42 | 0.53 |
| 349 | NFAT5 | rs1437134 | 0.40 | 0.13 | 1.9E-08 |
| 350 | NFATC1 | rs7236492 | 0.12 | 0.02 | 0.002 |
| 351 | NFATC1 | rs9518 | 0.19 | 0.39 | 4.5E-19 |
| 352 | NFATC3 | rs12598 | 0.08 | 0.01 | 0.004 |
| 353 | NFATC4 | rs2295298 | 0.36 | 0.17 | 3.8E-19 |
| 354 | NFKBIE | rs2282151 | 0.20 | 0.09 | 0.01 |
| 355 | NMI | rs1048135 | 0.43 | 0.22 | 2.5E-04 |
| 356 | NOD2 | rs3135499 | 0.42 | 0.46 | 0.03 |
| 357 | NOD2 | rs5743291 | 0.11 | 0.00 | 8.5E-05 |
| 358 | NONE | rs10229888 | 0.42 | 0.47 | 0.37 |
| 359 | NONE | rs11982525 | 0.20 | 0.27 | 0.07 |
| 360 | NONE | rs12029632 | 0.26 | 0.40 | 0.002 |
| 361 | NONE | rs13295258 | 0.23 | 0.35 | 0.01 |
| 362 | NONE | rs7582291 | 0.37 | 0.47 | 3.1E-05 |
| 363 | NONE | rs7881148 | 0.25 | 0.28 | 0.04 |
| 364 | NOS1 | rs816357 | 0.09 | 0.37 | 1.0E-10 |
| 365 | NR4A2 | rs12803 | 0.48 | 0.25 | 4.7E-06 |
| 366 | NR4A2 | rs834835 | 0.30 | 0.41 | 9.8E-08 |
| 367 | OAS1 | rs2660 | 0.37 | 0.11 | 2.7E-07 |
| 368 | OAS2 | rs1293764 | 0.33 | 0.13 | 6.9E-05 |
| 369 | OAS2 | rs13311 | 0.30 | 0.41 | 0.02 |
| 370 | OR2B11 | rs4925663 | 0.40 | 0.37 | 0.74 |
| 371 | PARP1 | rs1136410 | 0.15 | 0.16 | 0.92 |
| 372 | PARP1 | rs8679 | 0.20 | 0.05 | 0.001 |
| 373 | PAX5 | rs12552580 | 0.12 | 0.16 | 0.02 |
| 374 | PDCD1LG2 | rs7854413 | 0.09 | 0.38 | 4.6E-11 |
| 375 | PDCD5 | rs10500262 | 0.44 | 0.16 | 1.5E-13 |
| 376 | PDCD6IP | rs1127732 | 0.16 | 0.28 | 0.01 |
| 377 | PDF | rs1059519 | 0.32 | 0.23 | 0.23 |
| 378 | PDF | rs6512265 | 0.32 | 0.19 | 0.03 |
| 379 | PDGFRA | rs1547905 | 0.16 | 0.06 | 0.04 |
| 380 | PDGFRA | rs2228230 | 0.16 | 0.27 | 0.02 |
| 381 | PDGFRA | rs2229307 | 0.13 | 0.22 | 0.01 |
| 382 | PDGFRA | rs6554162 | 0.29 | 0.49 | 7.8E-05 |
| 383 | PECAM1 | rs1050382 | 0.46 | 0.41 | 0.05 |
| 384 | PECAM1 | rs1131012 | 0.46 | 0.26 | 2.5E-04 |
| 385 | PGLYRP2 | rs892145 | 0.39 | 0.41 | 0.29 |
| 386 | PGLYRP3 | rs843971 | 0.38 | 0.42 | 0.66 |
| 387 | PIAS4 | rs2289863 | 0.28 | 0.36 | 1.3E-15 |
| 388 | PIK3R1 | rs1043526 | 0.13 | 0.03 | 0.01 |
| 389 | PIK3R1 | rs3730089 | 0.14 | 0.28 | 0.001 |
| 390 | PILRB | rs705866 | 0.18 | 0.03 | 9.8E-07 |
| 391 | PIM1 | rs10507 | 0.31 | 0.33 | 0.81 |
| 392 | PLA2G2D | rs584367 | 0.43 | 0.12 | 2.3E-10 |
| 393 | PLA2G2D | rs617180 | 0.49 | 0.17 | 1.4E-09 |
| 394 | PLAU | rs2227551 | 0.28 | 0.41 | 0.01 |
| 395 | PLAU | rs2227564 | 0.23 | 0.16 | 0.15 |
| 396 | PLAU | rs4065 | 0.39 | 0.40 | 1.9E-04 |
| 397 | PLRG1 | rs12641958 | 0.31 | 0.26 | 0.41 |
| 398 | PLXNC1 | rs2291326 | 0.21 | 0.19 | 0.15 |
| 399 | PLXNC1 | rs2365736 | 0.37 | 0.36 | 0.98 |
| 400 | PNO1 | rs2044693 | 0.34 | 0.08 | 9.9E-09 |
| 401 | PPARA | rs6008259 | 0.21 | 0.47 | 1.3E-09 |
| 402 | PPARGC1B | rs7732671 | 0.09 | 0.17 | 0.01 |
| 403 | PPIC | rs451195 | 0.33 | 0.07 | 5.7E-09 |
| 404 | PPIE | rs562056 | 0.15 | 0.03 | 2.9E-04 |
| 405 | PPP1R15A | rs610308 | 0.31 | 0.43 | 4.9E-09 |
| 406 | PPP1R15A | rs611251 | 0.14 | 0.29 | 2.1E-04 |
| 407 | PPP2R2B | rs160974 | 0.39 | 0.27 | 6.2E-10 |
| 408 | PPP2R2B | rs161039 | 0.19 | 0.40 | 3.1E-05 |
| 409 | PPP2R2B | rs249907 | 0.26 | 0.44 | 2.7E-04 |
| 410 | PPP3R1 | rs2029091 | 0.34 | 0.36 | 0.78 |
| 411 | PRKCB | rs1015408 | 0.19 | 0.12 | 0.15 |
| 412 | PRKCB | rs2239339 | 0.16 | 0.09 | 0.19 |
| 413 | PRKCB | rs411103 | 0.39 | 0.37 | 3.9E-08 |
| 414 | PRKCE | rs6722418 | 0.16 | 0.17 | 0.50 |
| 415 | PRKCE | rs7582320 | 0.42 | 0.39 | 0.71 |
| 416 | PRKCQ | rs2236380 | 0.29 | 0.36 | 6.8E-13 |
| 417 | PRKCZ | rs3123592 | 0.11 | 0.09 | 0.90 |
| 418 | PTCRA | rs9471966 | 0.26 | 0.28 | 0.74 |
| 419 | PTEN | rs701848 | 0.41 | 0.29 | 0.03 |
| 420 | PTGER3 | rs5693 | 0.23 | 0.34 | 0.03 |
| 421 | PTGER3 | rs959 | 0.21 | 0.26 | 0.56 |
| 422 | PTGER4 | rs4957343 | 0.41 | 0.10 | 3.2E-10 |
| 423 | PTGIS | rs4647 | 0.25 | 0.15 | 0.03 |
| 424 | PTGIS | rs5600 | 0.26 | 0.16 | 0.04 |
| 425 | PTK2B | rs751019 | 0.44 | 0.29 | 0.004 |
| 426 | PTK2B | rs939269 | 0.26 | 0.43 | 1.4E-04 |
| 427 | PTPN12 | rs3750050 | 0.15 | 0.36 | 1.9E-06 |
| 428 | PTPN12 | rs9640663 | 0.41 | 0.16 | 1.3E-06 |
| 429 | PTPN13 | rs10033029 | 0.12 | 0.04 | 0.06 |
| 430 | PTPN13 | rs989902 | 0.44 | 0.27 | 4.5E-08 |
| 431 | PTPN22 | rs2476601 | 0.08 | 0.01 | 0.003 |
| 432 | PTPN22 | rs3811021 | 0.18 | 0.09 | 0.05 |
| 433 | PTPRB | rs2465811 | 0.28 | 0.33 | 0.53 |
| 434 | PTPRD | rs1353983 | 0.38 | 0.22 | 1.2E-12 |
| 435 | PTPRD | rs1836225 | 0.18 | 0.04 | 0.001 |
| 436 | PTPRD | rs2281747 | 0.15 | 0.20 | 0.18 |
| 437 | PTPRE | rs7081735 | 0.36 | 0.22 | 0.02 |
| 438 | PTPRE | rs7895103 | 0.26 | 0.22 | 0.71 |
| 439 | PTPRG | rs12629204 | 0.12 | 0.27 | 4.0E-04 |
| 440 | PTPRG | rs9808938 | 0.31 | 0.34 | 0.10 |
| 441 | PTPRJ | rs1503185 | 0.15 | 0.28 | 0.003 |
| 442 | PTPRJ | rs1566734 | 0.15 | 0.16 | 0.90 |
| 443 | PTPRJ | rs4752904 | 0.42 | 0.18 | 3.6E-14 |
| 444 | PTPRK | rs1417900 | 0.24 | 0.35 | 0.001 |
| 445 | PTPRR | rs10879183 | 0.21 | 0.15 | 0.11 |
| 446 | PTPRR | rs3803036 | 0.24 | 0.49 | 6.2E-11 |
| 447 | PTPRT | rs6065565 | 0.24 | 0.22 | 0.52 |
| 448 | PTPRU | rs2235937 | 0.24 | 0.34 | 0.07 |
| 449 | PTPRZ1 | rs1147504 | 0.47 | 0.21 | 1.5E-06 |
| 450 | PUS7L | rs1057190 | 0.09 | 0.36 | 1.1E-11 |
| 451 | PVRL2 | rs6859 | 0.41 | 0.41 | 0.80 |
| 452 | RAG1 | rs2227973 | 0.12 | 0.20 | 0.03 |
| 453 | RAG1 | rs3740955 | 0.35 | 0.28 | 8.9E-12 |
| 454 | S1PR2 | rs2116941 | 0.17 | 0.23 | 0.27 |
| 455 | S1PR3 | rs1867 | 0.21 | 0.05 | 2.2E-05 |
| 456 | SARM1 | rs2239907 | 0.44 | 0.41 | 0.75 |
| 457 | SARM1 | rs2239908 | 0.44 | 0.26 | 0.003 |
| 458 | SARM1 | rs739439 | 0.18 | 0.03 | 4.6E-05 |
| 459 | SELE | rs5361 | 0.10 | 0.05 | 0.32 |
| 460 | SELP | rs6131 | 0.18 | 0.25 | 0.24 |
| 461 | SELP | rs6133 | 0.13 | 0.47 | 2.9E-15 |
| 462 | SERPINB2 | rs9320030 | 0.19 | 0.37 | 1.1E-04 |
| 463 | SERPINE1 | rs2070682 | 0.41 | 0.47 | 0.47 |
| 464 | SH2B3 | rs3184504 | 0.49 | 0.05 | 7.0E-23 |
| 465 | SLAMF1 | rs2295612 | 0.18 | 0.03 | 3.6E-05 |
| 466 | SPINK5 | rs3777134 | 0.40 | 0.41 | 2.2E-05 |
| 467 | SPINK5 | rs6892205 | 0.49 | 0.46 | 0.74 |
| 468 | STAT2 | rs2066807 | 0.08 | 0.04 | 0.35 |
| 469 | STAT3 | rs1053023 | 0.18 | 0.39 | 1.0E-05 |
| 470 | STAT3 | rs3744483 | 0.18 | 0.39 | 1.0E-05 |
| 471 | STK17A | rs1044141 | 0.22 | 0.12 | 0.04 |
| 472 | SULT2A1 | rs2910393 | 0.25 | 0.15 | 0.09 |
| 473 | SYK | rs1049164 | 0.19 | 0.10 | 0.10 |
| 474 | TAP2 | rs13501 | 0.36 | 0.42 | 0.02 |
| 475 | TAP2 | rs241447 | 0.30 | 0.28 | 0.79 |
| 476 | TAPBP | rs2071888 | 0.47 | 0.40 | 0.32 |
| 477 | TBX21 | rs7502875 | 0.23 | 0.22 | 0.71 |
| 478 | TDGF1 | rs3737002 | 0.27 | 0.15 | 0.01 |
| 479 | TEC | rs3805184 | 0.25 | 0.25 | 0.98 |
| 480 | TESK1 | rs2275422 | 0.43 | 0.48 | 0.52 |
| 481 | TESK1 | rs7028425 | 0.38 | 0.34 | 0.57 |
| 482 | TGFB1 | rs1800471 | 0.06 | 0.06 | 0.13 |
| 483 | TGFB1 | rs2241718 | 0.18 | 0.15 | 0.56 |
| 484 | TGFB1 | rs2241720 | 0.18 | 0.17 | 0.85 |
| 485 | TGFB1 | rs6957 | 0.18 | 0.30 | 0.01 |
| 486 | TGFB1 | rs723877 | 0.30 | 0.44 | 0.02 |
| 487 | TGFB2 | rs2000220 | 0.44 | 0.18 | 9.6E-14 |
| 488 | TGFB2 | rs2796820 | 0.21 | 0.18 | 0.63 |
| 489 | TGFB3 | rs3917201 | 0.21 | 0.43 | 1.3E-07 |
| 490 | TGFBR1 | rs334349 | 0.26 | 0.41 | 0.005 |
| 491 | THPO | rs6141^a^ | 0.44 | 0.27 | 2.4E-07 |
| 492 | TICAM2 | rs256996 | 0.49 | 0.34 | 0.02 |
| 493 | TLR1 | rs4833095 | 0.27 | 0.29 | 3.6E-18 |
| 494 | TLR10 | rs11096955 | 0.34 | 0.46 | 2.2E-04 |
| 495 | TLR3 | rs3775291 | 0.28 | 0.19 | 0.05 |
| 496 | TLR5 | rs5744174 | 0.43 | 0.16 | 2.0E-07 |
| 497 | TLR6 | rs5743810 | 0.40 | 0.07 | 1.1E-12 |
| 498 | TLR7 | rs179008 | 0.21 | 0.08 | 0.02 |
| 499 | TLR8 | rs3764880 | 0.31 | 0.41 | 0.24 |
| 500 | TMCO6 | rs17208187 | 0.22 | 0.12 | 0.05 |
| 501 | TNFAIP6 | rs1046668 | 0.15 | 0.20 | 0.02 |
| 502 | TNFAIP6 | rs1046675 | 0.15 | 0.11 | 0.73 |
| 503 | TNFRSF10A | rs2230229 | 0.15 | 0.14 | 0.17 |
| 504 | TNFRSF10B | rs2889 | 0.34 | 0.17 | 0.001 |
| 505 | TNFRSF11A | rs1805034 | 0.46 | 0.36 | 0.02 |
| 506 | TNFRSF11B | rs3134054 | 0.36 | 0.46 | 4.0E-04 |
| 507 | TNFRSF11B | rs6469783 | 0.13 | 0.32 | 4.4E-06 |
| 508 | TNFRSF14 | rs2234167 | 0.12 | 0.04 | 0.03 |
| 509 | TNFRSF19 | rs3751364 | 0.21 | 0.39 | 0.001 |
| 510 | TRAF1 | rs4836834 | 0.42 | 0.23 | 3.1E-10 |
| 511 | TRAF3 | rs1131877 | 0.25 | 0.34 | 6.1E-17 |
| 512 | TRAF3IP2 | rs1043730 | 0.24 | 0.07 | 7.3E-05 |
| 513 | TRAP1 | rs13926 | 0.45 | 0.35 | 0.07 |
| 514 | TROVE2 | rs10737620 | 0.29 | 0.41 | 0.01 |
| 515 | TXK | rs11724347 | 0.07 | 0.01 | 0.01 |
| 516 | UNC84B | rs2072797 | 0.20 | 0.10 | 0.08 |
| 517 | VEGFA | rs3025000 | 0.30 | 0.16 | 4.4E-04 |
| 518 | VEGFA | rs3025010 | 0.41 | 0.35 | 0.56 |
| 519 | VEGFA | rs833068 | 0.31 | 0.47 | 0.01 |
| 520 | WDR92 | rs13009282 | 0.27 | 0.21 | 0.25 |
| 521 | ZAP70 | rs2276645 | 0.41 | 0.11 | 7.6E-09 |
| 522 | ZNF675 | rs11671053 | 0.36 | 0.30 | 6.7E-13 |
| ^a^Questionable genotype quality, site may have copy number variation | | | | | |

| **Supplementary Table 3: Odds Ratios (ORs), 95% Confidence Intervals (Cis) and p-values for the association between candidate SNPs and tumor mutation status in 279 GIST patients** | | | | | | | | | | |
| --- | --- | --- | --- | --- | --- | --- | --- | --- | --- | --- |
| **Gene Number** | **Gene** | **SNP** |  | **KIT exon 11 codon 557-558 deletion** | **KIT exon 11 insertion** | **KIT exon 11 other deletion** | **KIT exon 11 point mutation** | **Other KIT mutation** | **PDGFRA mutation** | **Wild type** |
| 1 | ABL1 | rs2987902 | OR (95% CI) | 0.79 (0.47, 1.34) | 0.56 (0.24, 1.29) | 1.16 (0.66, 2.03) | 1.34 (0.80, 2.25) | 1.08 (0.43, 2.70) | 1.09 (0.52, 2.25) | 0.98 (0.49, 1.95) |
|  |  |  | p-value | 0.4 | 0.2 | 0.6 | 0.3 | 0.9 | 0.8 | 1.0 |
| 2 | ABL1 | rs3824400 | OR (95% CI) | 0.84 (0.51, 1.39) | 0.94 (0.46, 1.92) | 1.07 (0.62, 1.85) | 1.33 (0.80, 2.22) | 0.71 (0.24, 2.05) | 0.82 (0.42, 1.63) | 0.71 (0.33, 1.54) |
|  |  |  | p-value | 0.5 | 0.9 | 0.8 | 0.3 | 0.5 | 0.6 | 0.4 |
| 3 | ACE | rs4343 | OR (95% CI) | 1.08 (0.73, 1.62) | 0.95 (0.54, 1.66) | 0.99 (0.62, 1.57) | 0.98 (0.64, 1.49) | 0.87 (0.44, 1.73) | 1.26 (0.72, 2.21) | 1.21 (0.71, 2.04) |
|  |  |  | p-value | 0.7 | 0.9 | 1.0 | 0.9 | 0.7 | 0.4 | 0.5 |
| 4 | ACTR8 | rs2241806 | OR (95% CI) | 1.11 (0.74, 1.66) | 0.72 (0.39, 1.31) | 1.04 (0.65, 1.67) | 1.19 (0.77, 1.82) | 1.12 (0.56, 2.26) | 1.29 (0.72, 2.31) | 0.97 (0.56, 1.69) |
|  |  |  | p-value | 0.6 | 0.3 | 0.9 | 0.4 | 0.7 | 0.4 | 0.9 |
| 5 | ADAR | rs2229857 | OR (95% CI) | 0.81 (0.52, 1.27) | 1.16 (0.64, 2.11) | 1.26 (0.77, 2.05) | 0.86 (0.54, 1.37) | 0.92 (0.42, 2.00) | 1.07 (0.58, 1.99) | 1.27 (0.72, 2.25) |
|  |  |  | p-value | 0.4 | 0.6 | 0.4 | 0.5 | 0.8 | 0.8 | 0.4 |
| 6 | ADAR | rs3738032 | OR (95% CI) | 0.78 (0.47, 1.31) | 0.79 (0.38, 1.65) | 1.26 (0.73, 2.17) | 0.68 (0.38, 1.20) | 0.97 (0.42, 2.27) | 0.86 (0.44, 1.68) | 2.00 (1.12, 3.58) |
|  |  |  | p-value | 0.4 | 0.5 | 0.4 | 0.2 | 0.9 | 0.7 | 0.02 |
| 7 | AGER | rs2288419 | OR (95% CI) | 1.02 (0.63, 1.65) | 0.91 (0.46, 1.82) | 0.88 (0.50, 1.56) | 1.17 (0.70, 1.93) | 1.98 (0.90, 4.38) | 1.23 (0.60, 2.54) | 0.78 (0.40, 1.51) |
|  |  |  | p-value | 1.0 | 0.8 | 0.7 | 0.5 | 0.09 | 0.6 | 0.5 |
| 8 | AHRR | rs2672725 | OR (95% CI) | 0.85 (0.49, 1.48) | 1.54 (0.77, 3.08) | 0.87 (0.46, 1.66) | 0.79 (0.43, 1.48) | 0.78 (0.28, 2.16) | 0.74 (0.37, 1.48) | 1.25 (0.65, 2.39) |
|  |  |  | p-value | 0.6 | 0.2 | 0.7 | 0.5 | 0.6 | 0.4 | 0.5 |
| 9 | AIF1 | rs2269475 | OR (95% CI) | 1.45 (0.81, 2.60) | 0.51 (0.17, 1.51) | 0.57 (0.25, 1.30) | 1.16 (0.61, 2.21) | 0.60 (0.18, 2.05) | 0.70 (0.32, 1.55) | 1.11 (0.53, 2.35) |
|  |  |  | p-value | 0.2 | 0.2 | 0.2 | 0.6 | 0.4 | 0.4 | 0.8 |
| 10 | AIFM2 | rs7893137 | OR (95% CI) | 1.07 (0.62, 1.84) | 2.29 (1.19, 4.39) | 0.67 (0.33, 1.35) | 0.97 (0.54, 1.74) | 0.43 (0.10, 1.79) | 1.46 (0.61, 3.51) | 1.17 (0.56, 2.45) |
|  |  |  | p-value | 0.8 | 0.01 | 0.3 | 0.9 | 0.2 | 0.4 | 0.7 |
| 11 | AKT3 | rs3006927 | OR (95% CI) | 0.63 (0.35, 1.14) | 1.44 (0.71, 2.90) | 0.66 (0.33, 1.33) | 1.33 (0.77, 2.28) | 1.04 (0.42, 2.62) | 0.80 (0.38, 1.68) | 1.19 (0.61, 2.30) |
|  |  |  | p-value | 0.1 | 0.3 | 0.3 | 0.3 | 0.9 | 0.6 | 0.6 |
| 12 | ALCAM | rs1044240 | OR (95% CI) | 1.08 (0.65, 1.81) | 1.21 (0.61, 2.42) | 1.09 (0.61, 1.97) | 1.34 (0.80, 2.26) | 0.73 (0.27, 1.94) | 1.07 (0.52, 2.21) | 0.53 (0.23, 1.21) |
|  |  |  | p-value | 0.8 | 0.6 | 0.8 | 0.3 | 0.5 | 0.9 | 0.1 |
| 13 | ALCAM | rs627925 | OR (95% CI) | 1.02 (0.68, 1.54) | 1.56 (0.91, 2.69) | 0.65 (0.38, 1.10) | 0.86 (0.54, 1.35) | 0.86 (0.41, 1.80) | 0.69 (0.41, 1.18) | 1.08 (0.64, 1.82) |
|  |  |  | p-value | 0.9 | 0.1 | 0.1 | 0.5 | 0.7 | 0.2 | 0.8 |
| 14 | ALCAM | rs7648171 | OR (95% CI) | 0.93 (0.60, 1.46) | 1.09 (0.58, 2.03) | 1.01 (0.61, 1.69) | 1.28 (0.81, 2.03) | 0.87 (0.39, 1.94) | 1.00 (0.53, 1.90) | 0.78 (0.43, 1.42) |
|  |  |  | p-value | 0.8 | 0.8 | 1.0 | 0.3 | 0.7 | 1.0 | 0.4 |
| 15 | ALDH3B1 | rs15518 | OR (95% CI) | 0.74 (0.48, 1.16) | 1.33 (0.75, 2.35) | 1.21 (0.75, 1.93) | 0.81 (0.51, 1.29) | 1.15 (0.56, 2.35) | 0.94 (0.52, 1.68) | 1.24 (0.73, 2.11) |
|  |  |  | p-value | 0.2 | 0.3 | 0.4 | 0.4 | 0.7 | 0.8 | 0.4 |
| 16 | ALDH3B1 | rs2286169 | OR (95% CI) | 0.71 (0.45, 1.12) | 1.39 (0.78, 2.48) | 1.20 (0.74, 1.94) | 0.75 (0.46, 1.22) | 1.15 (0.56, 2.39) | 0.75 (0.42, 1.33) | 1.16 (0.67, 2.02) |
|  |  |  | p-value | 0.1 | 0.3 | 0.5 | 0.2 | 0.7 | 0.3 | 0.6 |
| 17 | ALDH3B1 | rs581105 | OR (95% CI) | 1.00 (0.68, 1.47) | 0.65 (0.37, 1.13) | 0.93 (0.59, 1.45) | 1.28 (0.85, 1.93) | 0.76 (0.39, 1.48) | 0.83 (0.47, 1.44) | 1.00 (0.61, 1.65) |
|  |  |  | p-value | 1.0 | 0.1 | 0.7 | 0.2 | 0.4 | 0.5 | 1.0 |
| 18 | ALDH3B2 | rs1551886 | OR (95% CI) | 0.54 (0.28, 1.06) | 1.24 (0.55, 2.80) | 0.83 (0.41, 1.67) | 1.63 (0.91, 2.91) | 1.49 (0.56, 3.99) | 1.27 (0.52, 3.10) | 1.25 (0.58, 2.69) |
|  |  |  | p-value | 0.07 | 0.6 | 0.6 | 0.10 | 0.4 | 0.6 | 0.6 |
| 19 | APOL3 | rs132653 | OR (95% CI) | 1.06 (0.67, 1.67) | 1.11 (0.57, 2.14) | 1.43 (0.86, 2.40) | 0.93 (0.57, 1.54) | 0.95 (0.40, 2.26) | 1.20 (0.59, 2.41) | 0.59 (0.29, 1.20) |
|  |  |  | p-value | 0.8 | 0.8 | 0.2 | 0.8 | 0.9 | 0.6 | 0.1 |
| 20 | ATP6V1G2 | rs2071594 | OR (95% CI) | 1.05 (0.69, 1.60) | 1.43 (0.80, 2.56) | 1.08 (0.66, 1.75) | 0.90 (0.57, 1.41) | 0.65 (0.29, 1.43) | 1.25 (0.69, 2.27) | 1.05 (0.61, 1.82) |
|  |  |  | p-value | 0.8 | 0.2 | 0.8 | 0.6 | 0.3 | 0.5 | 0.9 |
| 21 | B3GNT3 | rs36686 | OR (95% CI) | 1.05 (0.66, 1.67) | 1.04 (0.54, 2.00) | 0.78 (0.44, 1.38) | 0.87 (0.52, 1.45) | 0.87 (0.37, 2.06) | 0.97 (0.50, 1.88) | 1.37 (0.75, 2.51) |
|  |  |  | p-value | 0.8 | 0.9 | 0.4 | 0.6 | 0.8 | 0.9 | 0.3 |
| 22 | BAG1 | rs706115 | OR (95% CI) | 1.14 (0.72, 1.81) | 1.27 (0.68, 2.38) | 0.65 (0.36, 1.17) | 1.31 (0.80, 2.13) | 0.70 (0.27, 1.85) | 1.32 (0.65, 2.65) | 0.90 (0.45, 1.80) |
|  |  |  | p-value | 0.6 | 0.5 | 0.1 | 0.3 | 0.5 | 0.4 | 0.8 |
| 23 | BAG3 | rs8946 | OR (95% CI) | 0.78 (0.51, 1.19) | 1.27 (0.72, 2.25) | 1.45 (0.91, 2.32) | 0.96 (0.62, 1.49) | 0.76 (0.36, 1.60) | 0.94 (0.53, 1.68) | 0.87 (0.51, 1.49) |
|  |  |  | p-value | 0.2 | 0.4 | 0.1 | 0.8 | 0.5 | 0.8 | 0.6 |
| 24 | BAG6 | rs1046089 | OR (95% CI) | 1.46 (0.97, 2.20) | 0.62 (0.33, 1.18) | 1.14 (0.71, 1.83) | 0.67 (0.42, 1.07) | 0.89 (0.42, 1.86) | 1.20 (0.67, 2.14) | 1.24 (0.72, 2.13) |
|  |  |  | p-value | 0.07 | 0.1 | 0.6 | 0.09 | 0.7 | 0.5 | 0.4 |
| 25 | BCL10 | rs2735592 | OR (95% CI) | 0.75 (0.49, 1.15) | 0.85 (0.47, 1.55) | 1.14 (0.72, 1.82) | 1.19 (0.78, 1.82) | 1.23 (0.62, 2.45) | 1.15 (0.63, 2.10) | 1.15 (0.68, 1.94) |
|  |  |  | p-value | 0.2 | 0.6 | 0.6 | 0.4 | 0.6 | 0.7 | 0.6 |
| 26 | BCL10 | rs962409 | OR (95% CI) | 1.17 (0.80, 1.71) | 0.97 (0.56, 1.66) | 1.32 (0.85, 2.06) | 0.90 (0.60, 1.36) | 1.04 (0.53, 2.05) | 1.68 (0.95, 2.98) | 0.92 (0.55, 1.54) |
|  |  |  | p-value | 0.4 | 0.9 | 0.2 | 0.6 | 0.9 | 0.08 | 0.8 |
| 27 | BCL2A1 | rs1138357 | OR (95% CI) | 1.19 (0.76, 1.87) | 0.96 (0.50, 1.85) | 1.48 (0.89, 2.46) | 0.77 (0.46, 1.28) | 0.70 (0.30, 1.65) | 1.51 (0.75, 3.05) | 1.07 (0.60, 1.92) |
|  |  |  | p-value | 0.4 | 0.9 | 0.1 | 0.3 | 0.4 | 0.3 | 0.8 |
| 28 | BCL2A1 | rs1138358 | OR (95% CI) | 0.97 (0.62, 1.49) | 1.23 (0.66, 2.27) | 1.65 (1.01, 2.69) | 0.75 (0.46, 1.22) | 0.73 (0.31, 1.75) | 1.48 (0.76, 2.90) | 1.09 (0.62, 1.91) |
|  |  |  | p-value | 0.9 | 0.5 | 0.05 | 0.2 | 0.5 | 0.3 | 0.8 |
| 29 | BCL2L13 | rs2535704 | OR (95% CI) | 0.79 (0.41, 1.51) | 1.31 (0.59, 2.87) | 1.01 (0.50, 2.05) | 1.61 (0.89, 2.93) | 1.17 (0.45, 3.02) | 2.84 (0.86, 9.37) | 0.98 (0.45, 2.13) |
|  |  |  | p-value | 0.5 | 0.5 | 1.0 | 0.1 | 0.7 | 0.09 | 1.0 |
| 30 | BCL2L13 | rs9306198 | OR (95% CI) | 0.63 (0.28, 1.42) | 1.43 (0.60, 3.44) | 1.22 (0.56, 2.65) | 1.75 (0.90, 3.42) | 1.24 (0.44, 3.51) | 2.84 (0.67, 11.99) | 0.74 (0.28, 1.93) |
|  |  |  | p-value | 0.3 | 0.4 | 0.6 | 0.1 | 0.7 | 0.2 | 0.5 |
| 31 | BCL2L14 | rs1797647 | OR (95% CI) | 0.85 (0.52, 1.39) | 0.85 (0.42, 1.70) | 1.37 (0.79, 2.36) | 1.50 (0.91, 2.47) | 0.91 (0.40, 2.07) | 0.93 (0.48, 1.82) | 0.55 (0.28, 1.09) |
|  |  |  | p-value | 0.5 | 0.6 | 0.3 | 0.1 | 0.8 | 0.8 | 0.09 |
| 32 | BCL2L14 | rs885720 | OR (95% CI) | 1.08 (0.70, 1.69) | 0.62 (0.31, 1.24) | 0.62 (0.36, 1.08) | 1.17 (0.73, 1.87) | 1.13 (0.50, 2.52) | 0.75 (0.41, 1.40) | 1.34 (0.74, 2.44) |
|  |  |  | p-value | 0.7 | 0.2 | 0.09 | 0.5 | 0.8 | 0.4 | 0.3 |
| 33 | BIRC5 | rs1042489 | OR (95% CI) | 1.15 (0.77, 1.72) | 1.88 (1.06, 3.32) | 0.92 (0.57, 1.47) | 0.84 (0.55, 1.31) | 0.51 (0.23, 1.12) | 1.64 (0.90, 3.01) | 1.39 (0.82, 2.36) |
|  |  |  | p-value | 0.5 | 0.03 | 0.7 | 0.4 | 0.09 | 0.1 | 0.2 |
| 34 | BIRC8 | rs8109165 | OR (95% CI) | 0.92 (0.60, 1.42) | 1.10 (0.61, 2.01) | 1.26 (0.77, 2.07) | 1.05 (0.67, 1.65) | 1.12 (0.54, 2.33) | 1.05 (0.57, 1.94) | 0.69 (0.39, 1.21) |
|  |  |  | p-value | 0.7 | 0.7 | 0.4 | 0.8 | 0.8 | 0.9 | 0.2 |
| 35 | BPI | rs1341022 | OR (95% CI) | 0.83 (0.54, 1.27) | 0.93 (0.51, 1.71) | 0.90 (0.55, 1.47) | 0.94 (0.60, 1.47) | 1.71 (0.82, 3.55) | 0.57 (0.31, 1.04) | 0.82 (0.47, 1.42) |
|  |  |  | p-value | 0.4 | 0.8 | 0.7 | 0.8 | 0.2 | 0.07 | 0.5 |
| 36 | BPI | rs4358188 | OR (95% CI) | 0.66 (0.43, 1.01) | 1.23 (0.69, 2.19) | 1.08 (0.67, 1.75) | 1.10 (0.71, 1.71) | 1.58 (0.76, 3.26) | 0.75 (0.42, 1.34) | 0.72 (0.41, 1.25) |
|  |  |  | p-value | 0.06 | 0.5 | 0.7 | 0.7 | 0.2 | 0.3 | 0.2 |
| 37 | BRSK1 | rs2286721 | OR (95% CI) | 1.21 (0.74, 1.98) | 0.76 (0.36, 1.65) | 1.07 (0.60, 1.90) | 0.76 (0.43, 1.36) | 1.00 (0.42, 2.40) | 0.78 (0.41, 1.49) | 0.96 (0.48, 1.89) |
|  |  |  | p-value | 0.5 | 0.5 | 0.8 | 0.4 | 1.0 | 0.5 | 0.9 |
| 38 | BRSK1 | rs2288523 | OR (95% CI) | 1.07 (0.62, 1.86) | 0.85 (0.37, 1.94) | 0.62 (0.30, 1.26) | 1.14 (0.63, 2.04) | 2.68 (1.12, 6.42) | 2.94 (1.00, 8.68) | 1.65 (0.82, 3.32) |
|  |  |  | p-value | 0.8 | 0.7 | 0.2 | 0.7 | 0.03 | 0.05 | 0.2 |
| 39 | BRSK1 | rs2532500 | OR (95% CI) | 0.82 (0.54, 1.24) | 1.42 (0.82, 2.47) | 1.05 (0.66, 1.67) | 0.95 (0.62, 1.46) | 1.93 (0.99, 3.75) | 1.26 (0.70, 2.24) | 0.88 (0.52, 1.50) |
|  |  |  | p-value | 0.3 | 0.2 | 0.8 | 0.8 | 0.05 | 0.4 | 0.6 |
| 40 | BST1 | rs2302465 | OR (95% CI) | 1.18 (0.67, 2.09) | 0.58 (0.21, 1.64) | 1.10 (0.56, 2.14) | 1.17 (0.63, 2.18) | 0.82 (0.29, 2.31) | 1.27 (0.54, 2.96) | 0.95 (0.45, 2.02) |
|  |  |  | p-value | 0.6 | 0.3 | 0.8 | 0.6 | 0.7 | 0.6 | 0.9 |
| 41 | C4BPA | rs4844573 | OR (95% CI) | 1.45 (1.00, 2.11) | 1.07 (0.63, 1.82) | 1.06 (0.69, 1.63) | 0.95 (0.64, 1.42) | 0.70 (0.35, 1.40) | 0.95 (0.57, 1.58) | 0.57 (0.34, 0.98) |
|  |  |  | p-value | 0.05 | 0.8 | 0.8 | 0.8 | 0.3 | 0.8 | 0.04 |
| 42 | C4BPB | rs6690037 | OR (95% CI) | 1.09 (0.74, 1.59) | 1.32 (0.77, 2.26) | 1.07 (0.69, 1.66) | 1.20 (0.80, 1.80) | 0.67 (0.34, 1.34) | 1.15 (0.67, 1.98) | 0.71 (0.43, 1.19) |
|  |  |  | p-value | 0.7 | 0.3 | 0.8 | 0.4 | 0.3 | 0.6 | 0.2 |
| 43 | C5 | rs17611 | OR (95% CI) | 0.75 (0.49, 1.13) | 1.18 (0.67, 2.08) | 0.65 (0.40, 1.06) | 1.39 (0.90, 2.14) | 0.93 (0.47, 1.83) | 1.02 (0.59, 1.77) | 1.49 (0.89, 2.48) |
|  |  |  | p-value | 0.2 | 0.6 | 0.08 | 0.1 | 0.8 | 0.9 | 0.1 |
| 44 | C6 | rs1801033 | OR (95% CI) | 0.79 (0.53, 1.17) | 0.75 (0.42, 1.33) | 1.35 (0.87, 2.09) | 1.15 (0.76, 1.73) | 1.61 (0.84, 3.08) | 1.62 (0.89, 2.93) | 1.21 (0.73, 1.98) |
|  |  |  | p-value | 0.2 | 0.3 | 0.2 | 0.5 | 0.1 | 0.1 | 0.5 |
| 45 | CAPN2 | rs10961 | OR (95% CI) | 0.81 (0.54, 1.23) | 1.30 (0.73, 2.33) | 0.81 (0.50, 1.32) | 1.38 (0.89, 2.15) | 2.00 (0.98, 4.10) | 1.38 (0.76, 2.50) | 0.87 (0.51, 1.49) |
|  |  |  | p-value | 0.3 | 0.4 | 0.4 | 0.1 | 0.06 | 0.3 | 0.6 |
| 46 | CARD14 | rs755340 | OR (95% CI) | 0.78 (0.48, 1.26) | 0.69 (0.33, 1.46) | 1.17 (0.69, 1.97) | 1.39 (0.86, 2.23) | 1.62 (0.76, 3.42) | 1.10 (0.55, 2.18) | 0.79 (0.42, 1.50) |
|  |  |  | p-value | 0.3 | 0.3 | 0.6 | 0.2 | 0.2 | 0.8 | 0.5 |
| 47 | CARD6 | rs10512747 | OR (95% CI) | 0.82 (0.43, 1.56) | 0.89 (0.37, 2.14) | 0.65 (0.29, 1.48) | 0.85 (0.43, 1.66) | 1.34 (0.53, 3.36) | 0.78 (0.35, 1.73) | 1.83 (0.92, 3.68) |
|  |  |  | p-value | 0.5 | 0.8 | 0.3 | 0.6 | 0.5 | 0.5 | 0.09 |
| 48 | CASP1 | rs2282659 | OR (95% CI) | 1.22 (0.79, 1.89) | 0.73 (0.37, 1.46) | 0.93 (0.55, 1.58) | 1.10 (0.69, 1.76) | 0.67 (0.29, 1.54) | 0.68 (0.37, 1.23) | 0.80 (0.45, 1.44) |
|  |  |  | p-value | 0.4 | 0.4 | 0.8 | 0.7 | 0.3 | 0.2 | 0.5 |
| 49 | CASP10 | rs13006529 | OR (95% CI) | 0.84 (0.56, 1.25) | 1.47 (0.83, 2.60) | 0.97 (0.61, 1.53) | 1.13 (0.74, 1.74) | 0.39 (0.18, 0.84) | 0.86 (0.49, 1.51) | 1.19 (0.70, 2.02) |
|  |  |  | p-value | 0.4 | 0.2 | 0.9 | 0.6 | 0.02 | 0.6 | 0.5 |
| 50 | CASP2 | rs3181166 | OR (95% CI) | 1.11 (0.63, 1.96) | 0.72 (0.29, 1.77) | 1.49 (0.80, 2.77) | 0.72 (0.37, 1.40) | 0.53 (0.16, 1.78) | 0.56 (0.28, 1.13) | 0.81 (0.37, 1.76) |
|  |  |  | p-value | 0.7 | 0.5 | 0.2 | 0.3 | 0.3 | 0.1 | 0.6 |
| 51 | CASP5 | rs507879 | OR (95% CI) | 0.96 (0.65, 1.40) | 0.87 (0.50, 1.49) | 1.00 (0.64, 1.55) | 1.23 (0.82, 1.84) | 0.66 (0.33, 1.32) | 1.03 (0.61, 1.76) | 1.17 (0.71, 1.95) |
|  |  |  | p-value | 0.8 | 0.6 | 1.0 | 0.3 | 0.2 | 0.9 | 0.5 |
| 52 | CASP5 | rs523104 | OR (95% CI) | 0.99 (0.67, 1.46) | 0.55 (0.31, 0.99) | 0.64 (0.40, 1.02) | 1.36 (0.90, 2.07) | 0.90 (0.45, 1.78) | 0.75 (0.43, 1.31) | 1.55 (0.92, 2.60) |
|  |  |  | p-value | 0.9 | 0.05 | 0.06 | 0.1 | 0.8 | 0.3 | 0.10 |
| 53 | CASP7 | rs4353229 | OR (95% CI) | 1.14 (0.74, 1.76) | 1.49 (0.84, 2.64) | 0.64 (0.36, 1.13) | 0.80 (0.50, 1.29) | 0.91 (0.43, 1.95) | 0.66 (0.37, 1.18) | 0.99 (0.56, 1.76) |
|  |  |  | p-value | 0.6 | 0.2 | 0.1 | 0.4 | 0.8 | 0.2 | 1.0 |
| 54 | CASP8 | rs3769823 | OR (95% CI) | 0.85 (0.55, 1.32) | 1.10 (0.59, 2.03) | 1.28 (0.78, 2.10) | 0.96 (0.60, 1.53) | 1.17 (0.54, 2.52) | 1.58 (0.81, 3.06) | 1.31 (0.73, 2.32) |
|  |  |  | p-value | 0.5 | 0.8 | 0.3 | 0.9 | 0.7 | 0.2 | 0.4 |
| 55 | CASP9 | rs2020902 | OR (95% CI) | 1.14 (0.63, 2.05) | 0.89 (0.38, 2.07) | 0.71 (0.33, 1.52) | 1.05 (0.56, 1.96) | 1.34 (0.52, 3.45) | 1.19 (0.50, 2.81) | 1.15 (0.53, 2.50) |
|  |  |  | p-value | 0.7 | 0.8 | 0.4 | 0.9 | 0.5 | 0.7 | 0.7 |
| 56 | CBLB | rs7649466 | OR (95% CI) | 0.54 (0.30, 0.99) | 1.15 (0.56, 2.36) | 1.36 (0.76, 2.41) | 1.33 (0.77, 2.30) | 0.26 (0.06, 1.14) | 0.70 (0.36, 1.39) | 0.99 (0.47, 2.06) |
|  |  |  | p-value | 0.05 | 0.7 | 0.3 | 0.3 | 0.07 | 0.3 | 1.0 |
| 57 | CBLB | rs894541 | OR (95% CI) | 0.96 (0.53, 1.74) | 1.13 (0.50, 2.52) | 1.10 (0.57, 2.14) | 1.27 (0.69, 2.32) | 0.65 (0.19, 2.24) | 1.34 (0.54, 3.33) | 0.80 (0.34, 1.91) |
|  |  |  | p-value | 0.9 | 0.8 | 0.8 | 0.4 | 0.5 | 0.5 | 0.6 |
| 58 | CCL16 | rs2063979 | OR (95% CI) | 1.02 (0.67, 1.55) | 1.70 (0.96, 3.03) | 1.13 (0.70, 1.83) | 0.94 (0.60, 1.46) | 1.01 (0.49, 2.07) | 1.33 (0.72, 2.45) | 0.71 (0.40, 1.26) |
|  |  |  | p-value | 0.9 | 0.07 | 0.6 | 0.8 | 1.0 | 0.4 | 0.2 |
| 59 | CCL26 | rs2302009 | OR (95% CI) | 1.11 (0.73, 1.69) | 0.82 (0.43, 1.55) | 0.85 (0.51, 1.41) | 0.79 (0.49, 1.27) | 0.58 (0.24, 1.40) | 0.52 (0.30, 0.90) | 1.14 (0.66, 2.00) |
|  |  |  | p-value | 0.6 | 0.5 | 0.5 | 0.3 | 0.2 | 0.02 | 0.6 |
| 60 | CCNE1 | rs1406 | OR (95% CI) | 1.23 (0.78, 1.95) | 0.72 (0.35, 1.50) | 1.03 (0.60, 1.77) | 0.78 (0.46, 1.34) | 0.44 (0.15, 1.29) | 0.66 (0.35, 1.24) | 1.27 (0.69, 2.31) |
|  |  |  | p-value | 0.4 | 0.4 | 0.9 | 0.4 | 0.1 | 0.2 | 0.4 |
| 61 | CCR8 | rs2853699 | OR (95% CI) | 1.06 (0.69, 1.62) | 1.01 (0.55, 1.86) | 0.99 (0.60, 1.64) | 0.56 (0.33, 0.94) | 1.00 (0.49, 2.06) | 0.75 (0.42, 1.31) | 1.42 (0.83, 2.42) |
|  |  |  | p-value | 0.8 | 1.0 | 1.0 | 0.03 | 1.0 | 0.3 | 0.2 |
| 62 | CD101 | rs3754112 | OR (95% CI) | 0.89 (0.57, 1.41) | 0.62 (0.31, 1.24) | 1.01 (0.60, 1.71) | 1.77 (1.11, 2.81) | 1.16 (0.57, 2.38) | 1.26 (0.66, 2.40) | 0.76 (0.42, 1.36) |
|  |  |  | p-value | 0.6 | 0.2 | 1.0 | 0.02 | 0.7 | 0.5 | 0.4 |
| 63 | CD109 | rs5023688 | OR (95% CI) | 0.93 (0.61, 1.41) | 0.97 (0.54, 1.76) | 1.15 (0.72, 1.85) | 1.52 (0.99, 2.35) | 0.79 (0.37, 1.67) | 1.45 (0.78, 2.73) | 0.81 (0.46, 1.41) |
|  |  |  | p-value | 0.7 | 0.9 | 0.6 | 0.06 | 0.5 | 0.2 | 0.5 |
| 64 | CD207 | rs17718987 | OR (95% CI) | 0.95 (0.57, 1.58) | 0.90 (0.44, 1.88) | 0.78 (0.42, 1.44) | 1.15 (0.67, 1.96) | 1.93 (0.86, 4.37) | 0.95 (0.47, 1.92) | 0.80 (0.42, 1.51) |
|  |  |  | p-value | 0.8 | 0.8 | 0.4 | 0.6 | 0.1 | 0.9 | 0.5 |
| 65 | CD207 | rs741326 | OR (95% CI) | 1.15 (0.77, 1.71) | 1.06 (0.60, 1.87) | 0.70 (0.43, 1.12) | 0.91 (0.60, 1.39) | 0.87 (0.44, 1.74) | 0.66 (0.37, 1.17) | 1.01 (0.61, 1.68) |
|  |  |  | p-value | 0.5 | 0.8 | 0.1 | 0.7 | 0.7 | 0.2 | 1.0 |
| 66 | CD209 | rs8105483 | OR (95% CI) | 1.41 (0.84, 2.35) | 1.04 (0.48, 2.22) | 0.49 (0.23, 1.06) | 1.05 (0.59, 1.86) | 1.04 (0.42, 2.59) | 0.89 (0.43, 1.85) | 0.97 (0.48, 1.93) |
|  |  |  | p-value | 0.2 | 0.9 | 0.07 | 0.9 | 0.9 | 0.8 | 0.9 |
| 67 | CD226 | rs763361 | OR (95% CI) | 1.13 (0.78, 1.65) | 1.22 (0.71, 2.08) | 0.68 (0.44, 1.07) | 1.02 (0.69, 1.52) | 1.00 (0.52, 1.93) | 0.86 (0.50, 1.47) | 0.94 (0.57, 1.53) |
|  |  |  | p-value | 0.5 | 0.5 | 0.10 | 0.9 | 1.0 | 0.6 | 0.8 |
| 68 | CD247 | rs1052231 | OR (95% CI) | 1.43 (0.89, 2.28) | 1.14 (0.58, 2.22) | 0.86 (0.48, 1.54) | 0.62 (0.35, 1.12) | 1.27 (0.57, 2.85) | 1.31 (0.62, 2.74) | 1.06 (0.56, 2.01) |
|  |  |  | p-value | 0.1 | 0.7 | 0.6 | 0.1 | 0.6 | 0.5 | 0.9 |
| 69 | CD27 | rs25680 | OR (95% CI) | 0.77 (0.48, 1.23) | 0.89 (0.46, 1.72) | 0.90 (0.52, 1.54) | 0.91 (0.56, 1.49) | 1.18 (0.56, 2.50) | 1.12 (0.57, 2.18) | 1.61 (0.93, 2.79) |
|  |  |  | p-value | 0.3 | 0.7 | 0.7 | 0.7 | 0.7 | 0.7 | 0.09 |
| 70 | CD274 | rs2297136 | OR (95% CI) | 1.08 (0.74, 1.57) | 0.86 (0.50, 1.50) | 0.89 (0.57, 1.39) | 1.12 (0.75, 1.68) | 1.18 (0.62, 2.26) | 0.78 (0.46, 1.33) | 0.71 (0.43, 1.18) |
|  |  |  | p-value | 0.7 | 0.6 | 0.6 | 0.6 | 0.6 | 0.4 | 0.2 |
| 71 | CD36 | rs7755 | OR (95% CI) | 1.03 (0.70, 1.52) | 0.93 (0.53, 1.61) | 1.04 (0.66, 1.64) | 1.04 (0.69, 1.57) | 0.88 (0.45, 1.73) | 0.81 (0.47, 1.41) | 0.87 (0.52, 1.46) |
|  |  |  | p-value | 0.9 | 0.8 | 0.9 | 0.9 | 0.7 | 0.5 | 0.6 |
| 72 | CD40 | rs1569723 | OR (95% CI) | 1.22 (0.79, 1.87) | 1.29 (0.71, 2.34) | 1.36 (0.84, 2.21) | 1.08 (0.68, 1.72) | 0.24 (0.07, 0.82) | 1.83 (0.88, 3.80) | 0.74 (0.39, 1.38) |
|  |  |  | p-value | 0.4 | 0.4 | 0.2 | 0.7 | 0.02 | 0.1 | 0.3 |
| 73 | CD44 | rs1467558 | OR (95% CI) | 1.86 (1.07, 3.25) | 1.10 (0.49, 2.44) | 0.37 (0.15, 0.92) | 1.11 (0.60, 2.03) | 0.40 (0.12, 1.39) | 0.67 (0.31, 1.43) | 0.78 (0.36, 1.67) |
|  |  |  | p-value | 0.03 | 0.8 | 0.03 | 0.7 | 0.2 | 0.3 | 0.5 |
| 74 | CD44 | rs353612 | OR (95% CI) | 1.28 (0.82, 1.98) | 0.99 (0.52, 1.90) | 1.36 (0.82, 2.26) | 0.62 (0.36, 1.06) | 0.42 (0.14, 1.20) | 0.49 (0.27, 0.89) | 0.52 (0.26, 1.06) |
|  |  |  | p-value | 0.3 | 1.0 | 0.2 | 0.08 | 0.1 | 0.02 | 0.07 |
| 75 | CD74 | rs15251 | OR (95% CI) | 0.78 (0.48, 1.27) | 1.23 (0.66, 2.30) | 1.16 (0.69, 1.97) | 0.66 (0.39, 1.14) | 0.98 (0.45, 2.11) | 0.67 (0.38, 1.21) | 1.25 (0.71, 2.22) |
|  |  |  | p-value | 0.3 | 0.5 | 0.6 | 0.1 | 1.0 | 0.2 | 0.4 |
| 76 | CD84 | rs3733264 | OR (95% CI) | 1.05 (0.67, 1.66) | 0.51 (0.24, 1.06) | 1.51 (0.90, 2.54) | 0.81 (0.49, 1.32) | 1.22 (0.57, 2.59) | 0.90 (0.48, 1.71) | 1.07 (0.59, 1.92) |
|  |  |  | p-value | 0.8 | 0.07 | 0.1 | 0.4 | 0.6 | 0.7 | 0.8 |
| 77 | CD8A | rs3020729 | OR (95% CI) | 0.81 (0.48, 1.35) | 0.87 (0.42, 1.79) | 1.30 (0.76, 2.23) | 0.90 (0.53, 1.54) | 1.30 (0.60, 2.84) | 0.78 (0.41, 1.51) | 0.94 (0.49, 1.80) |
|  |  |  | p-value | 0.4 | 0.7 | 0.3 | 0.7 | 0.5 | 0.5 | 0.9 |
| 78 | CD97 | rs2230748 | OR (95% CI) | 1.00 (0.57, 1.76) | 0.78 (0.32, 1.88) | 1.18 (0.63, 2.20) | 1.04 (0.57, 1.92) | 2.03 (0.86, 4.77) | 1.19 (0.52, 2.70) | 0.60 (0.26, 1.40) |
|  |  |  | p-value | 1.0 | 0.6 | 0.6 | 0.9 | 0.1 | 0.7 | 0.2 |
| 79 | CDK2 | rs2069391 | OR (95% CI) | 1.31 (0.60, 2.86) | 0.27 (0.04, 2.08) | 2.22 (0.99, 4.97) | 1.40 (0.60, 3.26) | 0.46 (0.06, 3.57) | 4.48 (0.59, 33.91) | 0.43 (0.10, 1.87) |
|  |  |  | p-value | 0.5 | 0.2 | 0.05 | 0.4 | 0.5 | 0.1 | 0.3 |
| 80 | CDK2 | rs2069398 | OR (95% CI) | 0.88 (0.45, 1.73) | 0.19 (0.03, 1.39) | 2.62 (1.34, 5.13) | 1.37 (0.68, 2.74) | 0.38 (0.05, 2.89) | 6.21 (0.84, 45.87) | 0.95 (0.35, 2.56) |
|  |  |  | p-value | 0.7 | 0.1 | 0.005 | 0.4 | 0.4 | 0.07 | 0.9 |
| 81 | CDKN2D | rs12984043 | OR (95% CI) | 0.91 (0.61, 1.36) | 0.97 (0.55, 1.73) | 1.09 (0.69, 1.73) | 1.01 (0.65, 1.55) | 1.26 (0.65, 2.42) | 0.85 (0.49, 1.46) | 0.82 (0.49, 1.37) |
|  |  |  | p-value | 0.6 | 0.9 | 0.7 | 1.0 | 0.5 | 0.6 | 0.4 |
| 82 | CFH | rs1065489 | OR (95% CI) | 1.28 (0.75, 2.16) | 0.67 (0.28, 1.63) | 1.27 (0.70, 2.33) | 0.91 (0.50, 1.66) | 0.47 (0.14, 1.58) | 1.22 (0.55, 2.71) | 1.13 (0.57, 2.23) |
|  |  |  | p-value | 0.4 | 0.4 | 0.4 | 0.8 | 0.2 | 0.6 | 0.7 |
| 83 | CFH | rs800292 | OR (95% CI) | 0.94 (0.61, 1.44) | 0.83 (0.45, 1.55) | 0.87 (0.53, 1.43) | 1.11 (0.71, 1.74) | 1.01 (0.47, 2.15) | 0.81 (0.44, 1.46) | 1.23 (0.71, 2.14) |
|  |  |  | p-value | 0.8 | 0.6 | 0.6 | 0.6 | 1.0 | 0.5 | 0.5 |
| 84 | CHIA | rs2256721 | OR (95% CI) | 0.70 (0.44, 1.11) | 1.30 (0.71, 2.40) | 1.23 (0.74, 2.05) | 1.05 (0.66, 1.69) | 0.81 (0.37, 1.80) | 0.91 (0.49, 1.68) | 0.92 (0.51, 1.64) |
|  |  |  | p-value | 0.1 | 0.4 | 0.4 | 0.8 | 0.6 | 0.8 | 0.8 |
| 85 | CHIA | rs2275254 | OR (95% CI) | 0.70 (0.46, 1.06) | 1.10 (0.63, 1.92) | 1.21 (0.76, 1.92) | 1.46 (0.95, 2.22) | 1.12 (0.57, 2.21) | 1.17 (0.66, 2.07) | 0.73 (0.43, 1.24) |
|  |  |  | p-value | 0.09 | 0.7 | 0.4 | 0.08 | 0.7 | 0.6 | 0.2 |
| 86 | CHIA | rs3818822 | OR (95% CI) | 0.83 (0.42, 1.62) | 0.83 (0.31, 2.24) | 1.01 (0.48, 2.13) | 1.84 (0.97, 3.49) | 1.79 (0.68, 4.70) | 1.38 (0.52, 3.69) | 0.50 (0.18, 1.36) |
|  |  |  | p-value | 0.6 | 0.7 | 1.0 | 0.06 | 0.2 | 0.5 | 0.2 |
| 87 | CHRNA2 | rs2280375 | OR (95% CI) | 0.72 (0.41, 1.29) | 0.91 (0.42, 2.01) | 0.76 (0.39, 1.48) | 1.91 (1.12, 3.26) | 0.66 (0.23, 1.92) | 0.91 (0.43, 1.90) | 0.94 (0.46, 1.91) |
|  |  |  | p-value | 0.3 | 0.8 | 0.4 | 0.02 | 0.4 | 0.8 | 0.9 |
| 88 | CIITA | rs3087519 | OR (95% CI) | 0.92 (0.60, 1.39) | 1.00 (0.56, 1.79) | 1.37 (0.85, 2.23) | 1.01 (0.65, 1.57) | 1.11 (0.54, 2.28) | 1.37 (0.76, 2.46) | 0.87 (0.50, 1.51) |
|  |  |  | p-value | 0.7 | 1.0 | 0.2 | 1.0 | 0.8 | 0.3 | 0.6 |
| 89 | CLCF1 | rs17608 | OR (95% CI) | 0.69 (0.44, 1.08) | 2.55 (1.38, 4.72) | 1.35 (0.83, 2.21) | 1.08 (0.68, 1.70) | 0.74 (0.34, 1.59) | 1.52 (0.79, 2.90) | 0.86 (0.50, 1.50) |
|  |  |  | p-value | 0.1 | 0.003 | 0.2 | 0.7 | 0.4 | 0.2 | 0.6 |
| 90 | CLEC4M | rs475896 | OR (95% CI) | 0.84 (0.57, 1.25) | 1.01 (0.57, 1.78) | 0.84 (0.53, 1.33) | 1.09 (0.72, 1.67) | 0.77 (0.39, 1.55) | 0.68 (0.39, 1.18) | 1.23 (0.74, 2.04) |
|  |  |  | p-value | 0.4 | 1.0 | 0.5 | 0.7 | 0.5 | 0.2 | 0.4 |
| 91 | CMTM6 | rs4796 | OR (95% CI) | 0.71 (0.46, 1.10) | 1.71 (0.95, 3.10) | 1.12 (0.68, 1.82) | 1.08 (0.69, 1.69) | 0.57 (0.26, 1.25) | 1.11 (0.61, 2.02) | 1.30 (0.75, 2.24) |
|  |  |  | p-value | 0.1 | 0.08 | 0.7 | 0.8 | 0.2 | 0.7 | 0.4 |
| 92 | CR2 | rs17615 | OR (95% CI) | 0.63 (0.40, 0.98) | 0.98 (0.54, 1.79) | 1.43 (0.90, 2.29) | 1.04 (0.66, 1.62) | 0.73 (0.33, 1.61) | 0.71 (0.40, 1.25) | 1.00 (0.58, 1.74) |
|  |  |  | p-value | 0.04 | 0.9 | 0.1 | 0.9 | 0.4 | 0.2 | 1.0 |
| 93 | CR2 | rs4308977 | OR (95% CI) | 0.67 (0.43, 1.02) | 0.91 (0.50, 1.67) | 1.50 (0.94, 2.38) | 0.96 (0.61, 1.49) | 0.82 (0.39, 1.75) | 0.72 (0.41, 1.26) | 1.00 (0.58, 1.72) |
|  |  |  | p-value | 0.06 | 0.8 | 0.09 | 0.8 | 0.6 | 0.2 | 1.0 |
| 94 | CR2 | rs6540433 | OR (95% CI) | 0.42 (0.20, 0.90) | 1.19 (0.50, 2.80) | 1.04 (0.50, 2.19) | 1.40 (0.74, 2.67) | 1.25 (0.47, 3.35) | 0.79 (0.34, 1.82) | 1.20 (0.55, 2.58) |
|  |  |  | p-value | 0.03 | 0.7 | 0.9 | 0.3 | 0.7 | 0.6 | 0.6 |
| 95 | CSF1 | rs333947 | OR (95% CI) | 1.01 (0.58, 1.73) | 1.18 (0.58, 2.40) | 0.89 (0.47, 1.70) | 0.87 (0.48, 1.58) | 1.49 (0.67, 3.35) | 2.24 (0.87, 5.72) | 1.67 (0.88, 3.17) |
|  |  |  | p-value | 1.0 | 0.6 | 0.7 | 0.7 | 0.3 | 0.09 | 0.1 |
| 96 | CUL5 | rs15677 | OR (95% CI) | 0.82 (0.52, 1.29) | 0.95 (0.50, 1.80) | 1.02 (0.61, 1.69) | 1.10 (0.69, 1.75) | 1.87 (0.93, 3.78) | 1.03 (0.54, 1.96) | 0.75 (0.40, 1.38) |
|  |  |  | p-value | 0.4 | 0.9 | 0.9 | 0.7 | 0.08 | 0.9 | 0.3 |
| 97 | CX3CR1 | rs3732378 | OR (95% CI) | 1.35 (0.78, 2.34) | 0.86 (0.38, 1.95) | 1.15 (0.60, 2.19) | 1.12 (0.62, 2.01) | 0.59 (0.20, 1.77) | 2.00 (0.76, 5.28) | 0.96 (0.46, 1.99) |
|  |  |  | p-value | 0.3 | 0.7 | 0.7 | 0.7 | 0.3 | 0.2 | 0.9 |
| 98 | CXCL10 | rs1063499 | OR (95% CI) | 1.43 (0.93, 2.21) | 1.42 (0.77, 2.62) | 1.06 (0.64, 1.74) | 0.69 (0.43, 1.10) | 1.20 (0.57, 2.53) | 1.70 (0.91, 3.20) | 0.91 (0.52, 1.61) |
|  |  |  | p-value | 0.1 | 0.3 | 0.8 | 0.1 | 0.6 | 0.10 | 0.8 |
| 99 | CXCL10 | rs13157656 | OR (95% CI) | 1.13 (0.69, 1.87) | 0.53 (0.23, 1.24) | 0.83 (0.45, 1.54) | 1.09 (0.64, 1.88) | 1.09 (0.47, 2.55) | 0.94 (0.47, 1.90) | 1.22 (0.65, 2.29) |
|  |  |  | p-value | 0.6 | 0.1 | 0.6 | 0.7 | 0.8 | 0.9 | 0.5 |
| 100 | CXCL16 | rs1050998 | OR (95% CI) | 0.87 (0.56, 1.35) | 0.81 (0.43, 1.52) | 1.20 (0.72, 1.98) | 1.03 (0.65, 1.64) | 1.07 (0.51, 2.25) | 0.95 (0.51, 1.75) | 1.01 (0.58, 1.76) |
|  |  |  | p-value | 0.5 | 0.5 | 0.5 | 0.9 | 0.9 | 0.9 | 1.0 |
| 101 | CXCL9 | rs3733236 | OR (95% CI) | 0.65 (0.32, 1.32) | 1.85 (0.79, 4.33) | 0.87 (0.40, 1.86) | 1.74 (0.90, 3.37) | 0.96 (0.27, 3.47) | 1.29 (0.47, 3.57) | 0.71 (0.26, 1.97) |
|  |  |  | p-value | 0.2 | 0.2 | 0.7 | 0.1 | 0.9 | 0.6 | 0.5 |
| 102 | CYP11A1 | rs11635047 | OR (95% CI) | 0.79 (0.52, 1.20) | 0.89 (0.50, 1.60) | 1.17 (0.73, 1.90) | 0.96 (0.61, 1.49) | 1.44 (0.71, 2.93) | 1.14 (0.65, 2.01) | 1.14 (0.68, 1.93) |
|  |  |  | p-value | 0.3 | 0.7 | 0.5 | 0.8 | 0.3 | 0.6 | 0.6 |
| 103 | CYP17A1 | rs6162 | OR (95% CI) | 1.05 (0.72, 1.53) | 0.80 (0.46, 1.38) | 1.31 (0.85, 2.03) | 0.69 (0.45, 1.04) | 1.11 (0.57, 2.16) | 0.77 (0.45, 1.31) | 1.08 (0.66, 1.76) |
|  |  |  | p-value | 0.8 | 0.4 | 0.2 | 0.08 | 0.7 | 0.3 | 0.8 |
| 104 | CYP19A1 | rs12148604 | OR (95% CI) | 1.16 (0.77, 1.74) | 1.04 (0.59, 1.83) | 0.84 (0.53, 1.35) | 0.89 (0.58, 1.37) | 0.52 (0.25, 1.09) | 0.70 (0.39, 1.25) | 1.13 (0.66, 1.92) |
|  |  |  | p-value | 0.5 | 0.9 | 0.5 | 0.6 | 0.08 | 0.2 | 0.7 |
| 105 | CYP19A1 | rs12591359 | OR (95% CI) | 0.86 (0.57, 1.28) | 0.92 (0.51, 1.64) | 0.97 (0.61, 1.56) | 1.17 (0.76, 1.79) | 1.38 (0.69, 2.74) | 1.36 (0.76, 2.44) | 1.17 (0.70, 1.95) |
|  |  |  | p-value | 0.5 | 0.8 | 0.9 | 0.5 | 0.4 | 0.3 | 0.6 |
| 106 | CYP19A1 | rs6493487 | OR (95% CI) | 0.82 (0.50, 1.34) | 1.09 (0.55, 2.18) | 0.83 (0.47, 1.49) | 1.38 (0.83, 2.28) | 1.71 (0.81, 3.64) | 1.10 (0.56, 2.18) | 0.74 (0.40, 1.38) |
|  |  |  | p-value | 0.4 | 0.8 | 0.5 | 0.2 | 0.2 | 0.8 | 0.3 |
| 107 | CYP19A1 | rs700518 | OR (95% CI) | 0.99 (0.66, 1.49) | 1.19 (0.67, 2.11) | 0.77 (0.47, 1.25) | 0.93 (0.60, 1.44) | 0.51 (0.24, 1.10) | 0.62 (0.35, 1.10) | 1.23 (0.72, 2.09) |
|  |  |  | p-value | 1.0 | 0.6 | 0.3 | 0.8 | 0.08 | 0.1 | 0.5 |
| 108 | CYP19A1 | rs934634 | OR (95% CI) | 1.02 (0.65, 1.59) | 1.12 (0.58, 2.13) | 0.73 (0.42, 1.27) | 1.32 (0.82, 2.12) | 1.43 (0.68, 2.99) | 0.94 (0.50, 1.77) | 0.64 (0.34, 1.21) |
|  |  |  | p-value | 0.9 | 0.7 | 0.3 | 0.2 | 0.3 | 0.9 | 0.2 |
| 109 | CYP24A1 | rs2296239 | OR (95% CI) | 0.93 (0.57, 1.51) | 0.81 (0.40, 1.65) | 0.80 (0.45, 1.43) | 0.84 (0.50, 1.43) | 2.09 (0.94, 4.61) | 0.73 (0.37, 1.41) | 1.07 (0.56, 2.04) |
|  |  |  | p-value | 0.8 | 0.6 | 0.5 | 0.5 | 0.07 | 0.3 | 0.8 |
| 110 | CYP24A1 | rs4809957 | OR (95% CI) | 0.94 (0.57, 1.52) | 0.82 (0.40, 1.66) | 0.81 (0.45, 1.44) | 0.81 (0.47, 1.38) | 2.11 (0.96, 4.66) | 0.72 (0.37, 1.40) | 1.09 (0.57, 2.09) |
|  |  |  | p-value | 0.8 | 0.6 | 0.5 | 0.4 | 0.06 | 0.3 | 0.8 |
| 111 | CYP24A1 | rs4809958 | OR (95% CI) | 0.76 (0.42, 1.40) | 1.27 (0.60, 2.72) | 1.05 (0.54, 2.05) | 1.13 (0.62, 2.04) | 0.85 (0.31, 2.38) | 1.63 (0.65, 4.08) | 1.36 (0.66, 2.80) |
|  |  |  | p-value | 0.4 | 0.5 | 0.9 | 0.7 | 0.8 | 0.3 | 0.4 |
| 112 | CYP24A1 | rs6068816 | OR (95% CI) | 0.86 (0.43, 1.72) | 1.08 (0.42, 2.73) | 1.01 (0.46, 2.19) | 1.39 (0.70, 2.74) | 0.70 (0.20, 2.53) | 2.33 (0.68, 7.96) | 1.47 (0.65, 3.36) |
|  |  |  | p-value | 0.7 | 0.9 | 1.0 | 0.3 | 0.6 | 0.2 | 0.4 |
| 113 | CYP27B1 | rs1048691 | OR (95% CI) | 0.98 (0.63, 1.53) | 0.80 (0.41, 1.58) | 0.86 (0.50, 1.46) | 1.52 (0.96, 2.40) | 1.17 (0.56, 2.43) | 1.34 (0.68, 2.64) | 0.83 (0.47, 1.48) |
|  |  |  | p-value | 0.9 | 0.5 | 0.6 | 0.08 | 0.7 | 0.4 | 0.5 |
| 114 | CYP2A7 | rs10419393 | OR (95% CI) | 0.81 (0.54, 1.19) | 1.60 (0.91, 2.82) | 1.17 (0.74, 1.83) | 1.04 (0.69, 1.58) | 0.91 (0.46, 1.79) | 0.95 (0.55, 1.62) | 0.76 (0.45, 1.27) |
|  |  |  | p-value | 0.3 | 0.1 | 0.5 | 0.8 | 0.8 | 0.8 | 0.3 |
| 115 | CYP2A7 | rs12461727 | OR (95% CI) | 1.08 (0.64, 1.79) | 0.99 (0.48, 2.05) | 0.88 (0.47, 1.63) | 1.28 (0.76, 2.17) | 0.80 (0.31, 2.07) | 1.29 (0.60, 2.78) | 1.00 (0.51, 1.96) |
|  |  |  | p-value | 0.8 | 1.0 | 0.7 | 0.4 | 0.6 | 0.5 | 1.0 |
| 116 | CYP2A7 | rs3869579 | OR (95% CI) | 0.70 (0.47, 1.05) | 1.47 (0.84, 2.57) | 1.23 (0.78, 1.93) | 1.14 (0.75, 1.74) | 1.15 (0.59, 2.23) | 0.91 (0.53, 1.56) | 0.71 (0.43, 1.19) |
|  |  |  | p-value | 0.08 | 0.2 | 0.4 | 0.5 | 0.7 | 0.7 | 0.2 |
| 117 | CYP2A7 | rs4803397 | OR (95% CI) | 1.56 (1.02, 2.40) | 0.66 (0.33, 1.30) | 0.74 (0.43, 1.27) | 0.76 (0.47, 1.24) | 1.00 (0.47, 2.09) | 0.95 (0.51, 1.77) | 1.22 (0.71, 2.09) |
|  |  |  | p-value | 0.04 | 0.2 | 0.3 | 0.3 | 1.0 | 0.9 | 0.5 |
| 118 | CYP2B6 | rs1042389 | OR (95% CI) | 0.97 (0.60, 1.57) | 1.90 (1.02, 3.55) | 1.08 (0.63, 1.85) | 0.64 (0.37, 1.12) | 1.09 (0.48, 2.49) | 1.69 (0.78, 3.66) | 1.26 (0.69, 2.31) |
|  |  |  | p-value | 0.9 | 0.04 | 0.8 | 0.1 | 0.8 | 0.2 | 0.4 |
| 119 | CYP2B6 | rs2054675 | OR (95% CI) | 0.81 (0.51, 1.30) | 1.57 (0.86, 2.86) | 1.00 (0.59, 1.69) | 1.49 (0.93, 2.39) | 0.72 (0.30, 1.72) | 0.81 (0.45, 1.48) | 0.47 (0.22, 0.99) |
|  |  |  | p-value | 0.4 | 0.1 | 1.0 | 0.09 | 0.5 | 0.5 | 0.05 |
| 120 | CYP2B6 | rs2099361 | OR (95% CI) | 0.97 (0.63, 1.48) | 0.65 (0.34, 1.24) | 0.84 (0.50, 1.39) | 1.26 (0.81, 1.97) | 1.26 (0.64, 2.51) | 1.01 (0.56, 1.82) | 1.11 (0.66, 1.88) |
|  |  |  | p-value | 0.9 | 0.2 | 0.5 | 0.3 | 0.5 | 1.0 | 0.7 |
| 121 | CYP2B6 | rs8100458 | OR (95% CI) | 1.33 (0.88, 2.02) | 0.85 (0.46, 1.57) | 1.00 (0.61, 1.64) | 0.55 (0.34, 0.91) | 0.99 (0.48, 2.01) | 0.99 (0.54, 1.79) | 1.39 (0.82, 2.34) |
|  |  |  | p-value | 0.2 | 0.6 | 1.0 | 0.02 | 1.0 | 1.0 | 0.2 |
| 122 | CYP2B6 | rs8192719 | OR (95% CI) | 0.84 (0.52, 1.34) | 1.71 (0.93, 3.13) | 0.91 (0.53, 1.56) | 1.55 (0.97, 2.50) | 0.70 (0.29, 1.71) | 0.81 (0.44, 1.49) | 0.43 (0.20, 0.93) |
|  |  |  | p-value | 0.5 | 0.08 | 0.7 | 0.07 | 0.4 | 0.5 | 0.03 |
| 123 | CYP2C19 | rs1042194 | OR (95% CI) | 1.51 (0.92, 2.47) | 1.25 (0.64, 2.43) | 0.80 (0.42, 1.51) | 0.69 (0.38, 1.26) | 0.72 (0.26, 2.03) | 0.59 (0.31, 1.13) | 0.50 (0.21, 1.22) |
|  |  |  | p-value | 0.1 | 0.5 | 0.5 | 0.2 | 0.5 | 0.1 | 0.1 |
| 124 | CYP2C19 | rs2281891 | OR (95% CI) | 1.49 (0.89, 2.49) | 1.34 (0.67, 2.66) | 0.77 (0.39, 1.51) | 0.74 (0.40, 1.36) | 0.76 (0.27, 2.17) | 0.67 (0.33, 1.35) | 0.54 (0.22, 1.32) |
|  |  |  | p-value | 0.1 | 0.4 | 0.4 | 0.3 | 0.6 | 0.3 | 0.2 |
| 125 | CYP2U1 | rs8727 | OR (95% CI) | 0.59 (0.35, 1.00) | 1.42 (0.75, 2.71) | 0.77 (0.43, 1.39) | 0.80 (0.47, 1.35) | 1.38 (0.64, 2.97) | 0.64 (0.34, 1.22) | 1.57 (0.88, 2.81) |
|  |  |  | p-value | 0.05 | 0.3 | 0.4 | 0.4 | 0.4 | 0.2 | 0.1 |
| 126 | CYP4B1 | rs2297809 | OR (95% CI) | 0.55 (0.28, 1.06) | 1.83 (0.93, 3.58) | 1.17 (0.63, 2.15) | 1.02 (0.57, 1.84) | 1.16 (0.43, 3.15) | 1.75 (0.68, 4.48) | 1.24 (0.57, 2.69) |
|  |  |  | p-value | 0.07 | 0.08 | 0.6 | 0.9 | 0.8 | 0.2 | 0.6 |
| 127 | CYP4B1 | rs4646487 | OR (95% CI) | 0.88 (0.48, 1.62) | 1.18 (0.52, 2.67) | 1.11 (0.57, 2.16) | 1.00 (0.52, 1.89) | 1.62 (0.62, 4.18) | 0.76 (0.34, 1.69) | 0.36 (0.12, 1.08) |
|  |  |  | p-value | 0.7 | 0.7 | 0.8 | 1.0 | 0.3 | 0.5 | 0.07 |
| 128 | CYP4B1 | rs837398 | OR (95% CI) | 0.74 (0.44, 1.22) | 0.80 (0.39, 1.65) | 1.78 (1.04, 3.02) | 1.05 (0.63, 1.75) | 1.47 (0.64, 3.37) | 1.01 (0.51, 2.01) | 0.53 (0.25, 1.15) |
|  |  |  | p-value | 0.2 | 0.6 | 0.03 | 0.9 | 0.4 | 1.0 | 0.1 |
| 129 | CYP4F2 | rs1272 | OR (95% CI) | 0.70 (0.42, 1.15) | 1.73 (0.94, 3.19) | 0.82 (0.46, 1.44) | 0.64 (0.37, 1.10) | 0.84 (0.35, 1.99) | 0.66 (0.35, 1.23) | 1.63 (0.91, 2.91) |
|  |  |  | p-value | 0.2 | 0.08 | 0.5 | 0.1 | 0.7 | 0.2 | 0.1 |
| 130 | CYP4F3 | rs1140855 | OR (95% CI) | 1.17 (0.66, 2.06) | 0.99 (0.44, 2.23) | 0.72 (0.35, 1.47) | 0.75 (0.39, 1.44) | 0.56 (0.18, 1.72) | 0.77 (0.36, 1.65) | 1.51 (0.75, 3.06) |
|  |  |  | p-value | 0.6 | 1.0 | 0.4 | 0.4 | 0.3 | 0.5 | 0.2 |
| 131 | CYP8B1 | rs12494055 | OR (95% CI) | 1.01 (0.67, 1.52) | 0.89 (0.49, 1.60) | 1.30 (0.81, 2.09) | 0.80 (0.51, 1.24) | 1.59 (0.79, 3.20) | 1.71 (0.94, 3.11) | 1.30 (0.77, 2.20) |
|  |  |  | p-value | 1.0 | 0.7 | 0.3 | 0.3 | 0.2 | 0.08 | 0.3 |
| 132 | CYP8B1 | rs2228468 | OR (95% CI) | 1.01 (0.68, 1.50) | 1.37 (0.78, 2.41) | 0.92 (0.58, 1.46) | 1.08 (0.70, 1.65) | 0.54 (0.25, 1.17) | 0.87 (0.50, 1.50) | 0.94 (0.56, 1.59) |
|  |  |  | p-value | 1.0 | 0.3 | 0.7 | 0.7 | 0.1 | 0.6 | 0.8 |
| 133 | CYP8B1 | rs3732860 | OR (95% CI) | 1.01 (0.67, 1.52) | 0.89 (0.49, 1.60) | 1.30 (0.81, 2.09) | 0.80 (0.51, 1.24) | 1.59 (0.79, 3.20) | 1.71 (0.94, 3.11) | 1.30 (0.77, 2.20) |
|  |  |  | p-value | 1.0 | 0.7 | 0.3 | 0.3 | 0.2 | 0.08 | 0.3 |
| 134 | DAP | rs5484 | OR (95% CI) | 1.09 (0.65, 1.82) | 1.37 (0.69, 2.74) | 0.90 (0.48, 1.66) | 0.70 (0.39, 1.27) | 0.48 (0.16, 1.49) | 0.79 (0.38, 1.61) | 1.49 (0.76, 2.94) |
|  |  |  | p-value | 0.8 | 0.4 | 0.7 | 0.2 | 0.2 | 0.5 | 0.2 |
| 135 | DAP | rs9857 | OR (95% CI) | 0.90 (0.57, 1.40) | 0.52 (0.25, 1.07) | 1.56 (0.95, 2.53) | 1.38 (0.88, 2.18) | 1.16 (0.54, 2.47) | 1.07 (0.56, 2.02) | 0.62 (0.33, 1.18) |
|  |  |  | p-value | 0.6 | 0.08 | 0.08 | 0.2 | 0.7 | 0.8 | 0.1 |
| 136 | DAPK1 | rs1056719 | OR (95% CI) | 1.32 (0.87, 2.00) | 0.96 (0.53, 1.76) | 1.06 (0.66, 1.72) | 0.97 (0.61, 1.52) | 0.44 (0.19, 1.02) | 1.76 (0.94, 3.30) | 1.39 (0.80, 2.42) |
|  |  |  | p-value | 0.2 | 0.9 | 0.8 | 0.9 | 0.06 | 0.08 | 0.2 |
| 137 | DARC | rs12075 | OR (95% CI) | 1.05 (0.72, 1.53) | 0.86 (0.51, 1.48) | 0.94 (0.61, 1.46) | 1.26 (0.85, 1.87) | 1.42 (0.73, 2.75) | 1.18 (0.69, 2.02) | 0.69 (0.40, 1.18) |
|  |  |  | p-value | 0.8 | 0.6 | 0.8 | 0.3 | 0.3 | 0.5 | 0.2 |
| 138 | DLG5 | rs1248696 | OR (95% CI) | 1.67 (0.91, 3.06) | 0.00 (0.00, 0.00) | 1.21 (0.59, 2.48) | 0.80 (0.38, 1.70) | 0.60 (0.15, 2.50) | 0.63 (0.29, 1.37) | 0.84 (0.32, 2.19) |
|  |  |  | p-value | 0.10 | 0.9 | 0.6 | 0.6 | 0.5 | 0.2 | 0.7 |
| 139 | DNAJA3 | rs4785963 | OR (95% CI) | 0.61 (0.38, 0.97) | 0.70 (0.36, 1.34) | 1.64 (1.02, 2.65) | 1.26 (0.80, 1.99) | 1.09 (0.52, 2.28) | 0.73 (0.41, 1.31) | 0.81 (0.45, 1.44) |
|  |  |  | p-value | 0.04 | 0.3 | 0.04 | 0.3 | 0.8 | 0.3 | 0.5 |
| 140 | DOCK2 | rs3734099 | OR (95% CI) | 0.83 (0.48, 1.45) | 0.55 (0.22, 1.40) | 1.18 (0.65, 2.12) | 0.58 (0.30, 1.12) | 0.80 (0.28, 2.33) | 0.49 (0.25, 0.96) | 1.89 (0.98, 3.63) |
|  |  |  | p-value | 0.5 | 0.2 | 0.6 | 0.1 | 0.7 | 0.04 | 0.06 |
| 141 | DPP4 | rs3788979 | OR (95% CI) | 0.72 (0.39, 1.33) | 0.73 (0.29, 1.80) | 1.02 (0.54, 1.93) | 1.05 (0.58, 1.89) | 0.71 (0.22, 2.26) | 0.62 (0.31, 1.25) | 1.27 (0.64, 2.52) |
|  |  |  | p-value | 0.3 | 0.5 | 0.9 | 0.9 | 0.6 | 0.2 | 0.5 |
| 142 | DUSP10 | rs6670514 | OR (95% CI) | 1.37 (0.91, 2.06) | 0.77 (0.41, 1.47) | 1.25 (0.78, 2.00) | 0.80 (0.50, 1.27) | 0.62 (0.27, 1.40) | 0.77 (0.44, 1.36) | 0.71 (0.41, 1.25) |
|  |  |  | p-value | 0.1 | 0.4 | 0.3 | 0.3 | 0.2 | 0.4 | 0.2 |
| 143 | DUSP2 | rs1724120 | OR (95% CI) | 1.09 (0.74, 1.61) | 0.52 (0.29, 0.94) | 1.01 (0.65, 1.58) | 1.24 (0.82, 1.89) | 0.72 (0.36, 1.44) | 0.94 (0.55, 1.63) | 1.24 (0.74, 2.07) |
|  |  |  | p-value | 0.7 | 0.03 | 1.0 | 0.3 | 0.3 | 0.8 | 0.4 |
| 144 | DUSP6 | rs770087 | OR (95% CI) | 1.08 (0.68, 1.73) | 0.79 (0.38, 1.66) | 1.03 (0.59, 1.77) | 1.12 (0.68, 1.85) | 0.52 (0.18, 1.49) | 1.35 (0.64, 2.84) | 1.37 (0.77, 2.46) |
|  |  |  | p-value | 0.7 | 0.5 | 0.9 | 0.7 | 0.2 | 0.4 | 0.3 |
| 145 | EDAR | rs5021634 | OR (95% CI) | 0.98 (0.59, 1.64) | 1.51 (0.72, 3.16) | 0.94 (0.52, 1.70) | 1.00 (0.58, 1.75) | 0.89 (0.33, 2.38) | 1.72 (0.77, 3.86) | 1.28 (0.64, 2.56) |
|  |  |  | p-value | 0.9 | 0.3 | 0.8 | 1.0 | 0.8 | 0.2 | 0.5 |
| 146 | EDARADD | rs966365 | OR (95% CI) | 1.08 (0.68, 1.70) | 1.10 (0.57, 2.12) | 1.29 (0.78, 2.15) | 0.73 (0.42, 1.25) | 0.94 (0.40, 2.25) | 1.07 (0.55, 2.09) | 1.04 (0.55, 1.96) |
|  |  |  | p-value | 0.8 | 0.8 | 0.3 | 0.2 | 0.9 | 0.8 | 0.9 |
| 147 | EGF | rs2237051 | OR (95% CI) | 0.87 (0.59, 1.29) | 1.10 (0.63, 1.93) | 0.69 (0.43, 1.09) | 1.53 (0.99, 2.35) | 1.41 (0.72, 2.78) | 1.72 (0.97, 3.05) | 1.35 (0.80, 2.28) |
|  |  |  | p-value | 0.5 | 0.7 | 0.1 | 0.06 | 0.3 | 0.06 | 0.3 |
| 148 | EGF | rs4698803 | OR (95% CI) | 0.99 (0.59, 1.67) | 0.84 (0.39, 1.83) | 1.19 (0.66, 2.12) | 0.99 (0.57, 1.74) | 0.54 (0.19, 1.55) | 0.80 (0.41, 1.54) | 0.98 (0.51, 1.88) |
|  |  |  | p-value | 1.0 | 0.7 | 0.6 | 1.0 | 0.3 | 0.5 | 0.9 |
| 149 | EGFR | rs10228436 | OR (95% CI) | 1.05 (0.69, 1.60) | 0.80 (0.44, 1.46) | 0.86 (0.52, 1.40) | 0.95 (0.61, 1.48) | 1.16 (0.56, 2.40) | 0.86 (0.48, 1.54) | 1.14 (0.65, 2.01) |
|  |  |  | p-value | 0.8 | 0.5 | 0.5 | 0.8 | 0.7 | 0.6 | 0.6 |
| 150 | EGFR | rs10277413 | OR (95% CI) | 1.03 (0.68, 1.56) | 0.79 (0.44, 1.43) | 0.90 (0.55, 1.46) | 0.93 (0.60, 1.44) | 1.13 (0.55, 2.32) | 0.87 (0.49, 1.56) | 1.21 (0.70, 2.09) |
|  |  |  | p-value | 0.9 | 0.4 | 0.7 | 0.7 | 0.7 | 0.6 | 0.5 |
| 151 | EGFR | rs4947982^a^ | OR (95% CI) | 1.05 (0.71, 1.56) | 1.27 (0.73, 2.22) | 1.10 (0.70, 1.74) | 0.99 (0.65, 1.51) | 0.73 (0.35, 1.50) | 0.78 (0.45, 1.37) | 0.61 (0.35, 1.07) |
|  |  |  | p-value | 0.8 | 0.4 | 0.7 | 1.0 | 0.4 | 0.4 | 0.08 |
| 152 | EGFR | rs887826 | OR (95% CI) | 0.98 (0.55, 1.76) | 0.65 (0.25, 1.69) | 1.70 (0.93, 3.11) | 0.72 (0.36, 1.42) | 0.60 (0.18, 1.96) | 0.78 (0.37, 1.65) | 0.97 (0.47, 2.03) |
|  |  |  | p-value | 1.0 | 0.4 | 0.09 | 0.3 | 0.4 | 0.5 | 0.9 |
| 153 | ENTPD1 | rs3181115 | OR (95% CI) | 0.83 (0.40, 1.73) | 1.03 (0.38, 2.79) | 0.89 (0.38, 2.08) | 1.79 (0.90, 3.56) | 1.51 (0.55, 4.18) | 3.20 (0.75, 13.70) | 0.88 (0.37, 2.11) |
|  |  |  | p-value | 0.6 | 1.0 | 0.8 | 0.10 | 0.4 | 0.1 | 0.8 |
| 154 | ESR1 | rs3020371 | OR (95% CI) | 1.04 (0.69, 1.57) | 1.07 (0.60, 1.89) | 1.35 (0.85, 2.16) | 1.13 (0.73, 1.74) | 0.51 (0.23, 1.16) | 1.11 (0.62, 1.98) | 0.82 (0.47, 1.43) |
|  |  |  | p-value | 0.8 | 0.8 | 0.2 | 0.6 | 0.1 | 0.7 | 0.5 |
| 155 | ESR1 | rs3798577 | OR (95% CI) | 1.24 (0.84, 1.84) | 0.50 (0.27, 0.92) | 0.91 (0.58, 1.44) | 1.33 (0.87, 2.04) | 0.73 (0.37, 1.46) | 0.96 (0.55, 1.66) | 1.01 (0.61, 1.67) |
|  |  |  | p-value | 0.3 | 0.03 | 0.7 | 0.2 | 0.4 | 0.9 | 1.0 |
| 156 | ESR1 | rs6557171 | OR (95% CI) | 0.83 (0.56, 1.23) | 1.42 (0.82, 2.46) | 0.94 (0.60, 1.48) | 0.72 (0.47, 1.12) | 0.55 (0.26, 1.17) | 1.03 (0.60, 1.77) | 2.26 (1.35, 3.79) |
|  |  |  | p-value | 0.3 | 0.2 | 0.8 | 0.1 | 0.1 | 0.9 | 0.002 |
| 157 | ESR1 | rs974276 | OR (95% CI) | 1.61 (0.98, 2.64) | 0.53 (0.21, 1.32) | 1.27 (0.71, 2.26) | 1.08 (0.62, 1.88) | 0.56 (0.17, 1.86) | 1.75 (0.71, 4.33) | 0.82 (0.39, 1.74) |
|  |  |  | p-value | 0.06 | 0.2 | 0.4 | 0.8 | 0.3 | 0.2 | 0.6 |
| 158 | ETS1 | rs4372467 | OR (95% CI) | 1.14 (0.77, 1.68) | 0.79 (0.45, 1.40) | 1.08 (0.69, 1.69) | 0.75 (0.49, 1.15) | 1.32 (0.68, 2.55) | 1.13 (0.65, 1.96) | 1.37 (0.83, 2.26) |
|  |  |  | p-value | 0.5 | 0.4 | 0.7 | 0.2 | 0.4 | 0.7 | 0.2 |
| 159 | ETS1 | rs4937333 | OR (95% CI) | 1.20 (0.80, 1.80) | 0.93 (0.52, 1.67) | 0.94 (0.58, 1.51) | 1.24 (0.80, 1.92) | 1.12 (0.56, 2.26) | 1.14 (0.64, 2.05) | 0.68 (0.40, 1.17) |
|  |  |  | p-value | 0.4 | 0.8 | 0.8 | 0.3 | 0.7 | 0.7 | 0.2 |
| 160 | ETS1 | rs8705 | OR (95% CI) | 1.11 (0.70, 1.75) | 0.89 (0.46, 1.74) | 0.67 (0.38, 1.19) | 0.92 (0.56, 1.51) | 1.11 (0.53, 2.34) | 0.80 (0.43, 1.48) | 1.17 (0.67, 2.06) |
|  |  |  | p-value | 0.7 | 0.7 | 0.2 | 0.7 | 0.8 | 0.5 | 0.6 |
| 161 | F11R | rs1062827 | OR (95% CI) | 0.81 (0.50, 1.31) | 0.98 (0.50, 1.90) | 0.69 (0.39, 1.22) | 1.64 (1.01, 2.67) | 1.21 (0.56, 2.58) | 0.94 (0.49, 1.80) | 0.80 (0.44, 1.46) |
|  |  |  | p-value | 0.4 | 1.0 | 0.2 | 0.04 | 0.6 | 0.9 | 0.5 |
| 162 | F13A1 | rs1050783 | OR (95% CI) | 0.62 (0.34, 1.15) | 0.80 (0.34, 1.86) | 1.11 (0.59, 2.08) | 0.36 (0.16, 0.78) | 1.97 (0.85, 4.56) | 0.31 (0.16, 0.61) | 1.33 (0.66, 2.68) |
|  |  |  | p-value | 0.1 | 0.6 | 0.8 | 0.01 | 0.1 | 0.0007 | 0.4 |
| 163 | FAM3B | rs417708 | OR (95% CI) | 1.04 (0.58, 1.87) | 0.47 (0.16, 1.37) | 1.10 (0.57, 2.16) | 0.92 (0.49, 1.74) | 1.65 (0.70, 3.90) | 1.23 (0.51, 2.99) | 1.28 (0.63, 2.60) |
|  |  |  | p-value | 0.9 | 0.2 | 0.8 | 0.8 | 0.3 | 0.6 | 0.5 |
| 164 | FAS | rs2234978 | OR (95% CI) | 0.72 (0.46, 1.14) | 0.93 (0.49, 1.75) | 1.09 (0.66, 1.79) | 1.13 (0.71, 1.81) | 0.38 (0.14, 1.00) | 0.50 (0.28, 0.90) | 1.17 (0.67, 2.05) |
|  |  |  | p-value | 0.2 | 0.8 | 0.7 | 0.6 | 0.05 | 0.02 | 0.6 |
| 165 | FASLG | rs10458360 | OR (95% CI) | 1.11 (0.73, 1.67) | 0.74 (0.41, 1.33) | 1.19 (0.74, 1.92) | 1.02 (0.66, 1.58) | 1.07 (0.53, 2.17) | 1.05 (0.59, 1.90) | 0.79 (0.46, 1.35) |
|  |  |  | p-value | 0.6 | 0.3 | 0.5 | 0.9 | 0.9 | 0.9 | 0.4 |
| 166 | FCAR | rs16986050 | OR (95% CI) | 0.86 (0.52, 1.45) | 1.10 (0.54, 2.21) | 1.39 (0.81, 2.38) | 1.06 (0.63, 1.79) | 0.68 (0.24, 1.91) | 1.13 (0.53, 2.42) | 0.98 (0.50, 1.92) |
|  |  |  | p-value | 0.6 | 0.8 | 0.2 | 0.8 | 0.5 | 0.8 | 1.0 |
| 167 | FCER1G | rs11421 | OR (95% CI) | 0.88 (0.52, 1.50) | 1.07 (0.53, 2.18) | 2.13 (1.24, 3.64) | 0.78 (0.43, 1.39) | 0.74 (0.28, 1.94) | 1.10 (0.53, 2.28) | 0.75 (0.37, 1.53) |
|  |  |  | p-value | 0.6 | 0.8 | 0.006 | 0.4 | 0.5 | 0.8 | 0.4 |
| 168 | FCGR2B | rs1801274 | OR (95% CI) | 1.04 (0.70, 1.54) | 1.02 (0.59, 1.77) | 0.99 (0.63, 1.56) | 0.70 (0.46, 1.07) | 1.42 (0.72, 2.81) | 0.78 (0.45, 1.36) | 1.04 (0.62, 1.73) |
|  |  |  | p-value | 0.8 | 0.9 | 1.0 | 0.1 | 0.3 | 0.4 | 0.9 |
| 169 | FGA | rs2070022 | OR (95% CI) | 1.35 (0.81, 2.25) | 1.05 (0.51, 2.16) | 0.77 (0.40, 1.47) | 1.45 (0.85, 2.47) | 1.41 (0.60, 3.28) | 2.05 (0.83, 5.05) | 0.42 (0.17, 1.02) |
|  |  |  | p-value | 0.2 | 0.9 | 0.4 | 0.2 | 0.4 | 0.1 | 0.06 |
| 170 | FGA | rs6050 | OR (95% CI) | 0.77 (0.50, 1.20) | 1.15 (0.64, 2.04) | 0.87 (0.53, 1.43) | 0.97 (0.62, 1.52) | 0.79 (0.36, 1.73) | 1.03 (0.57, 1.86) | 2.02 (1.19, 3.44) |
|  |  |  | p-value | 0.2 | 0.6 | 0.6 | 0.9 | 0.5 | 0.9 | 0.009 |
| 171 | FGB | rs4220 | OR (95% CI) | 1.05 (0.64, 1.74) | 1.07 (0.52, 2.18) | 1.34 (0.76, 2.36) | 0.88 (0.51, 1.52) | 1.11 (0.48, 2.56) | 1.27 (0.59, 2.75) | 0.85 (0.44, 1.63) |
|  |  |  | p-value | 0.8 | 0.9 | 0.3 | 0.6 | 0.8 | 0.5 | 0.6 |
| 172 | FLT3 | rs1933437 | OR (95% CI) | 0.86 (0.57, 1.30) | 0.73 (0.40, 1.32) | 1.25 (0.78, 2.00) | 0.77 (0.49, 1.20) | 1.00 (0.50, 2.02) | 0.84 (0.48, 1.47) | 1.50 (0.89, 2.55) |
|  |  |  | p-value | 0.5 | 0.3 | 0.3 | 0.2 | 1.0 | 0.5 | 0.1 |
| 173 | FOS | rs1569328 | OR (95% CI) | 0.67 (0.35, 1.28) | 1.29 (0.59, 2.82) | 1.06 (0.53, 2.09) | 0.75 (0.39, 1.47) | 1.16 (0.46, 2.94) | 0.77 (0.36, 1.65) | 1.39 (0.70, 2.76) |
|  |  |  | p-value | 0.2 | 0.5 | 0.9 | 0.4 | 0.8 | 0.5 | 0.3 |
| 174 | FPR1 | rs2070745 | OR (95% CI) | 0.92 (0.61, 1.38) | 1.12 (0.63, 1.98) | 1.10 (0.70, 1.75) | 1.04 (0.67, 1.60) | 0.79 (0.38, 1.65) | 1.87 (1.00, 3.50) | 1.65 (0.98, 2.78) |
|  |  |  | p-value | 0.7 | 0.7 | 0.7 | 0.9 | 0.5 | 0.05 | 0.06 |
| 175 | FPR1 | rs5030878 | OR (95% CI) | 0.77 (0.46, 1.27) | 1.20 (0.63, 2.28) | 1.06 (0.61, 1.84) | 1.22 (0.74, 2.00) | 2.15 (1.05, 4.41) | 1.29 (0.63, 2.64) | 0.55 (0.27, 1.13) |
|  |  |  | p-value | 0.3 | 0.6 | 0.8 | 0.4 | 0.04 | 0.5 | 0.1 |
| 176 | FPR1 | rs867228 | OR (95% CI) | 1.44 (0.92, 2.24) | 1.37 (0.74, 2.51) | 0.54 (0.29, 1.01) | 1.32 (0.83, 2.12) | 0.85 (0.36, 2.00) | 3.78 (1.34, 10.69) | 1.06 (0.58, 1.94) |
|  |  |  | p-value | 0.1 | 0.3 | 0.05 | 0.2 | 0.7 | 0.01 | 0.9 |
| 177 | FYB | rs358501 | OR (95% CI) | 1.15 (0.73, 1.81) | 0.66 (0.31, 1.40) | 1.29 (0.78, 2.13) | 1.21 (0.75, 1.95) | 0.38 (0.12, 1.23) | 1.16 (0.60, 2.24) | 0.96 (0.52, 1.78) |
|  |  |  | p-value | 0.5 | 0.3 | 0.3 | 0.4 | 0.1 | 0.7 | 0.9 |
| 178 | FYB | rs379707 | OR (95% CI) | 0.91 (0.59, 1.39) | 1.56 (0.86, 2.81) | 1.25 (0.77, 2.04) | 0.95 (0.60, 1.49) | 0.93 (0.44, 1.99) | 1.41 (0.76, 2.62) | 0.88 (0.49, 1.57) |
|  |  |  | p-value | 0.7 | 0.1 | 0.4 | 0.8 | 0.9 | 0.3 | 0.7 |
| 179 | GATA3 | rs1058240 | OR (95% CI) | 1.00 (0.62, 1.62) | 1.34 (0.69, 2.59) | 0.64 (0.34, 1.18) | 1.04 (0.62, 1.75) | 2.23 (1.02, 4.91) | 1.00 (0.50, 1.98) | 0.69 (0.34, 1.39) |
|  |  |  | p-value | 1.0 | 0.4 | 0.1 | 0.9 | 0.05 | 1.0 | 0.3 |
| 180 | GATA3 | rs3802604 | OR (95% CI) | 1.32 (0.88, 1.99) | 1.06 (0.60, 1.89) | 0.85 (0.53, 1.37) | 1.03 (0.67, 1.60) | 1.02 (0.50, 2.09) | 1.28 (0.71, 2.29) | 0.80 (0.46, 1.40) |
|  |  |  | p-value | 0.2 | 0.8 | 0.5 | 0.9 | 1.0 | 0.4 | 0.4 |
| 181 | GBP7 | rs676913 | OR (95% CI) | 0.96 (0.63, 1.47) | 0.98 (0.54, 1.77) | 0.74 (0.44, 1.23) | 0.92 (0.59, 1.44) | 0.54 (0.24, 1.20) | 0.65 (0.37, 1.15) | 1.61 (0.96, 2.70) |
|  |  |  | p-value | 0.9 | 0.9 | 0.2 | 0.7 | 0.1 | 0.1 | 0.07 |
| 182 | GHR | rs6180 | OR (95% CI) | 0.89 (0.60, 1.32) | 1.14 (0.65, 2.00) | 0.86 (0.55, 1.36) | 1.14 (0.75, 1.74) | 1.72 (0.86, 3.45) | 1.39 (0.79, 2.44) | 1.00 (0.60, 1.69) |
|  |  |  | p-value | 0.6 | 0.6 | 0.5 | 0.5 | 0.1 | 0.3 | 1.0 |
| 183 | GMIP | rs880090 | OR (95% CI) | 0.80 (0.52, 1.24) | 2.20 (1.23, 3.94) | 0.90 (0.55, 1.49) | 0.93 (0.59, 1.48) | 0.90 (0.42, 1.93) | 0.89 (0.50, 1.60) | 0.92 (0.51, 1.64) |
|  |  |  | p-value | 0.3 | 0.008 | 0.7 | 0.8 | 0.8 | 0.7 | 0.8 |
| 184 | GPR15 | rs2230344 | OR (95% CI) | 0.94 (0.54, 1.63) | 1.22 (0.60, 2.48) | 0.64 (0.31, 1.32) | 1.07 (0.60, 1.89) | 0.64 (0.21, 1.98) | 1.19 (0.55, 2.56) | 1.79 (0.96, 3.30) |
|  |  |  | p-value | 0.8 | 0.6 | 0.2 | 0.8 | 0.4 | 0.7 | 0.06 |
| 185 | GPX6 | rs406113 | OR (95% CI) | 1.09 (0.71, 1.65) | 0.67 (0.35, 1.26) | 0.94 (0.58, 1.54) | 1.31 (0.84, 2.05) | 1.10 (0.53, 2.25) | 1.50 (0.80, 2.81) | 1.17 (0.68, 2.01) |
|  |  |  | p-value | 0.7 | 0.2 | 0.8 | 0.2 | 0.8 | 0.2 | 0.6 |
| 186 | GRN | rs5848 | OR (95% CI) | 1.78 (1.17, 2.73) | 1.10 (0.60, 2.00) | 0.70 (0.42, 1.17) | 1.15 (0.74, 1.81) | 0.64 (0.28, 1.48) | 1.96 (1.00, 3.84) | 0.95 (0.54, 1.68) |
|  |  |  | p-value | 0.007 | 0.8 | 0.2 | 0.5 | 0.3 | 0.05 | 0.9 |
| 187 | GSTM3 | rs3814309 | OR (95% CI) | 1.51 (1.00, 2.27) | 0.90 (0.49, 1.64) | 0.59 (0.35, 1.00) | 1.21 (0.78, 1.88) | 0.49 (0.21, 1.17) | 1.04 (0.58, 1.87) | 1.05 (0.60, 1.84) |
|  |  |  | p-value | 0.05 | 0.7 | 0.05 | 0.4 | 0.1 | 0.9 | 0.9 |
| 188 | GSTM3 | rs7483 | OR (95% CI) | 1.48 (0.97, 2.25) | 0.93 (0.51, 1.73) | 0.61 (0.36, 1.04) | 1.15 (0.73, 1.81) | 0.50 (0.21, 1.22) | 0.97 (0.53, 1.78) | 1.02 (0.57, 1.82) |
|  |  |  | p-value | 0.07 | 0.8 | 0.07 | 0.5 | 0.1 | 0.9 | 0.9 |
| 189 | GZMB | rs2236338 | OR (95% CI) | 1.34 (0.85, 2.13) | 1.93 (1.05, 3.56) | 0.68 (0.38, 1.23) | 0.91 (0.55, 1.52) | 0.64 (0.26, 1.59) | 0.90 (0.47, 1.73) | 0.66 (0.34, 1.30) |
|  |  |  | p-value | 0.2 | 0.04 | 0.2 | 0.7 | 0.3 | 0.8 | 0.2 |
| 190 | GZMB | rs8192917 | OR (95% CI) | 1.16 (0.74, 1.82) | 2.33 (1.28, 4.24) | 0.71 (0.41, 1.25) | 1.06 (0.66, 1.72) | 0.50 (0.19, 1.33) | 0.98 (0.51, 1.86) | 0.65 (0.33, 1.26) |
|  |  |  | p-value | 0.5 | 0.006 | 0.2 | 0.8 | 0.2 | 0.9 | 0.2 |
| 191 | HAVCR1 | rs1553316 | OR (95% CI) | 0.80 (0.46, 1.39) | 1.11 (0.52, 2.37) | 1.20 (0.66, 2.18) | 1.11 (0.63, 1.96) | 1.00 (0.40, 2.49) | 0.83 (0.40, 1.69) | 0.83 (0.41, 1.68) |
|  |  |  | p-value | 0.4 | 0.8 | 0.6 | 0.7 | 1.0 | 0.6 | 0.6 |
| 192 | HDAC4 | rs1962113 | OR (95% CI) | 0.85 (0.55, 1.33) | 0.83 (0.44, 1.58) | 1.09 (0.66, 1.79) | 1.05 (0.66, 1.69) | 0.49 (0.20, 1.17) | 1.00 (0.55, 1.80) | 1.68 (0.97, 2.90) |
|  |  |  | p-value | 0.5 | 0.6 | 0.7 | 0.8 | 0.1 | 1.0 | 0.07 |
| 193 | HDAC4 | rs870790 | OR (95% CI) | 1.26 (0.84, 1.88) | 0.85 (0.47, 1.54) | 0.97 (0.60, 1.55) | 0.72 (0.46, 1.14) | 1.15 (0.57, 2.30) | 1.42 (0.78, 2.59) | 1.58 (0.93, 2.69) |
|  |  |  | p-value | 0.3 | 0.6 | 0.9 | 0.2 | 0.7 | 0.3 | 0.09 |
| 194 | HLA-DMA | rs1063478 | OR (95% CI) | 0.73 (0.40, 1.32) | 1.95 (0.96, 3.98) | 0.33 (0.13, 0.80) | 1.92 (1.09, 3.37) | 0.78 (0.27, 2.32) | 0.76 (0.35, 1.64) | 0.65 (0.28, 1.50) |
|  |  |  | p-value | 0.3 | 0.07 | 0.01 | 0.02 | 0.7 | 0.5 | 0.3 |
| 195 | HLA-DPA1 | rs1042190 | OR (95% CI) | 1.15 (0.70, 1.89) | 1.28 (0.64, 2.53) | 0.88 (0.49, 1.58) | 1.35 (0.80, 2.27) | 0.53 (0.18, 1.59) | 0.88 (0.44, 1.79) | 0.56 (0.25, 1.22) |
|  |  |  | p-value | 0.6 | 0.5 | 0.7 | 0.3 | 0.3 | 0.7 | 0.1 |
| 196 | HLA-DPA1 | rs3077 | OR (95% CI) | 1.18 (0.72, 1.91) | 1.24 (0.63, 2.45) | 0.85 (0.47, 1.52) | 1.39 (0.84, 2.33) | 0.52 (0.18, 1.57) | 0.91 (0.45, 1.83) | 0.55 (0.25, 1.21) |
|  |  |  | p-value | 0.5 | 0.5 | 0.6 | 0.2 | 0.2 | 0.8 | 0.1 |
| 197 | HLA-DRA | rs7192 | OR (95% CI) | 1.14 (0.76, 1.70) | 0.46 (0.24, 0.88) | 0.90 (0.56, 1.45) | 0.97 (0.63, 1.49) | 1.93 (0.97, 3.86) | 1.24 (0.69, 2.24) | 1.43 (0.85, 2.39) |
|  |  |  | p-value | 0.5 | 0.02 | 0.7 | 0.9 | 0.06 | 0.5 | 0.2 |
| 198 | HLA-DRA | rs8084 | OR (95% CI) | 1.13 (0.77, 1.68) | 0.48 (0.26, 0.90) | 0.89 (0.56, 1.41) | 0.92 (0.61, 1.40) | 2.27 (1.13, 4.57) | 1.16 (0.65, 2.05) | 1.33 (0.81, 2.20) |
|  |  |  | p-value | 0.5 | 0.02 | 0.6 | 0.7 | 0.02 | 0.6 | 0.3 |
| 199 | HSP90B1 | rs1882019 | OR (95% CI) | 0.90 (0.43, 1.86) | 0.88 (0.30, 2.54) | 0.96 (0.41, 2.21) | 0.85 (0.39, 1.89) | 2.20 (0.83, 5.85) | 1.87 (0.55, 6.35) | 1.23 (0.52, 2.90) |
|  |  |  | p-value | 0.8 | 0.8 | 0.9 | 0.7 | 0.1 | 0.3 | 0.6 |
| 200 | HSP90B1 | rs2164747 | OR (95% CI) | 0.94 (0.55, 1.61) | 0.37 (0.14, 0.99) | 1.53 (0.86, 2.72) | 0.76 (0.42, 1.37) | 2.25 (0.96, 5.31) | 1.08 (0.50, 2.33) | 1.25 (0.60, 2.57) |
|  |  |  | p-value | 0.8 | 0.05 | 0.1 | 0.4 | 0.06 | 0.9 | 0.6 |
| 201 | IFI16 | rs1057027 | OR (95% CI) | 1.37 (0.88, 2.14) | 2.04 (1.12, 3.71) | 0.68 (0.38, 1.20) | 0.98 (0.60, 1.60) | 1.11 (0.50, 2.47) | 2.03 (0.92, 4.49) | 0.78 (0.41, 1.49) |
|  |  |  | p-value | 0.2 | 0.02 | 0.2 | 0.9 | 0.8 | 0.08 | 0.5 |
| 202 | IFI16 | rs6940 | OR (95% CI) | 1.44 (0.88, 2.34) | 1.67 (0.88, 3.20) | 0.74 (0.39, 1.41) | 0.86 (0.49, 1.52) | 1.30 (0.56, 3.02) | 2.19 (0.85, 5.64) | 0.82 (0.40, 1.69) |
|  |  |  | p-value | 0.1 | 0.1 | 0.4 | 0.6 | 0.5 | 0.1 | 0.6 |
| 203 | IFI16 | rs866484 | OR (95% CI) | 1.48 (0.95, 2.30) | 2.36 (1.27, 4.40) | 0.72 (0.41, 1.24) | 0.74 (0.45, 1.22) | 0.92 (0.40, 2.11) | 2.08 (0.96, 4.50) | 0.99 (0.53, 1.83) |
|  |  |  | p-value | 0.08 | 0.007 | 0.2 | 0.2 | 0.8 | 0.06 | 1.0 |
| 204 | IFI35 | rs10840 | OR (95% CI) | 1.49 (0.81, 2.74) | 1.38 (0.60, 3.16) | 1.16 (0.56, 2.39) | 0.64 (0.29, 1.40) | 0.64 (0.15, 2.71) | 5.67 (0.77, 41.84) | 1.47 (0.65, 3.35) |
|  |  |  | p-value | 0.2 | 0.4 | 0.7 | 0.3 | 0.5 | 0.09 | 0.4 |
| 205 | IFIH1 | rs3747517 | OR (95% CI) | 1.13 (0.75, 1.71) | 0.76 (0.40, 1.43) | 1.20 (0.75, 1.93) | 1.02 (0.66, 1.59) | 0.63 (0.28, 1.44) | 1.29 (0.68, 2.45) | 1.19 (0.70, 2.02) |
|  |  |  | p-value | 0.6 | 0.4 | 0.4 | 0.9 | 0.3 | 0.4 | 0.5 |
| 206 | IFNAR1 | rs2834202 | OR (95% CI) | 1.06 (0.68, 1.66) | 1.12 (0.61, 2.06) | 1.78 (1.09, 2.91) | 0.60 (0.35, 1.01) | 1.20 (0.56, 2.55) | 1.50 (0.76, 2.93) | 0.89 (0.48, 1.64) |
|  |  |  | p-value | 0.8 | 0.7 | 0.02 | 0.06 | 0.6 | 0.2 | 0.7 |
| 207 | IFNGR1 | rs3799488 | OR (95% CI) | 1.70 (0.91, 3.16) | 1.12 (0.45, 2.81) | 0.26 (0.08, 0.88) | 0.76 (0.35, 1.63) | 1.83 (0.70, 4.78) | 1.01 (0.40, 2.56) | 1.08 (0.47, 2.48) |
|  |  |  | p-value | 0.09 | 0.8 | 0.03 | 0.5 | 0.2 | 1.0 | 0.9 |
| 208 | IFNGR2 | rs9808753 | OR (95% CI) | 1.20 (0.72, 1.99) | 0.39 (0.14, 1.10) | 1.22 (0.69, 2.16) | 0.71 (0.39, 1.29) | 1.51 (0.64, 3.59) | 1.11 (0.51, 2.43) | 1.37 (0.70, 2.68) |
|  |  |  | p-value | 0.5 | 0.07 | 0.5 | 0.3 | 0.3 | 0.8 | 0.4 |
| 209 | IGF1 | rs6214 | OR (95% CI) | 1.01 (0.68, 1.50) | 0.77 (0.43, 1.37) | 1.39 (0.88, 2.19) | 0.73 (0.47, 1.13) | 0.96 (0.48, 1.91) | 0.74 (0.42, 1.28) | 0.97 (0.57, 1.62) |
|  |  |  | p-value | 1.0 | 0.4 | 0.2 | 0.2 | 0.9 | 0.3 | 0.9 |
| 210 | IGF1R | rs8030950 | OR (95% CI) | 0.76 (0.49, 1.17) | 1.76 (0.99, 3.10) | 0.75 (0.45, 1.25) | 1.29 (0.83, 2.01) | 1.80 (0.91, 3.55) | 1.05 (0.58, 1.91) | 0.64 (0.35, 1.15) |
|  |  |  | p-value | 0.2 | 0.05 | 0.3 | 0.3 | 0.09 | 0.9 | 0.1 |
| 211 | IGFBP3 | rs6670 | OR (95% CI) | 0.90 (0.54, 1.50) | 0.92 (0.45, 1.90) | 0.97 (0.54, 1.74) | 1.01 (0.59, 1.73) | 1.24 (0.56, 2.76) | 0.70 (0.37, 1.31) | 0.63 (0.31, 1.32) |
|  |  |  | p-value | 0.7 | 0.8 | 0.9 | 1.0 | 0.6 | 0.3 | 0.2 |
| 212 | IGLL1 | rs131429 | OR (95% CI) | 0.77 (0.51, 1.16) | 1.17 (0.67, 2.06) | 1.09 (0.69, 1.72) | 1.21 (0.79, 1.86) | 1.48 (0.73, 2.97) | 1.69 (0.92, 3.10) | 0.95 (0.56, 1.64) |
|  |  |  | p-value | 0.2 | 0.6 | 0.7 | 0.4 | 0.3 | 0.09 | 0.9 |
| 213 | IL10 | rs3024498 | OR (95% CI) | 1.08 (0.67, 1.73) | 0.62 (0.30, 1.29) | 1.09 (0.63, 1.88) | 0.79 (0.47, 1.32) | 0.56 (0.22, 1.39) | 0.31 (0.16, 0.60) | 0.75 (0.39, 1.45) |
|  |  |  | p-value | 0.8 | 0.2 | 0.8 | 0.4 | 0.2 | 0.0004 | 0.4 |
| 214 | IL10RA | rs2512143 | OR (95% CI) | 1.10 (0.75, 1.62) | 0.74 (0.41, 1.33) | 1.23 (0.79, 1.91) | 1.00 (0.66, 1.52) | 1.22 (0.63, 2.35) | 0.99 (0.57, 1.72) | 0.74 (0.44, 1.26) |
|  |  |  | p-value | 0.6 | 0.3 | 0.4 | 1.0 | 0.6 | 1.0 | 0.3 |
| 215 | IL10RB | rs2834167 | OR (95% CI) | 1.44 (0.90, 2.29) | 1.18 (0.61, 2.28) | 0.87 (0.50, 1.54) | 1.04 (0.63, 1.73) | 0.39 (0.13, 1.17) | 0.89 (0.45, 1.75) | 0.70 (0.36, 1.38) |
|  |  |  | p-value | 0.1 | 0.6 | 0.6 | 0.9 | 0.09 | 0.7 | 0.3 |
| 216 | IL11 | rs1042506 | OR (95% CI) | 0.53 (0.26, 1.06) | 0.59 (0.22, 1.60) | 1.23 (0.62, 2.43) | 1.76 (0.96, 3.25) | 2.11 (0.83, 5.34) | 0.81 (0.36, 1.85) | 0.62 (0.25, 1.50) |
|  |  |  | p-value | 0.07 | 0.3 | 0.6 | 0.07 | 0.1 | 0.6 | 0.3 |
| 217 | IL11RA | rs3808868 | OR (95% CI) | 0.81 (0.55, 1.20) | 0.81 (0.45, 1.43) | 1.18 (0.75, 1.85) | 1.02 (0.67, 1.55) | 0.81 (0.41, 1.59) | 0.74 (0.43, 1.28) | 1.10 (0.67, 1.81) |
|  |  |  | p-value | 0.3 | 0.5 | 0.5 | 0.9 | 0.5 | 0.3 | 0.7 |
| 218 | IL12A | rs583911 | OR (95% CI) | 0.70 (0.46, 1.07) | 1.30 (0.74, 2.29) | 0.88 (0.55, 1.41) | 1.14 (0.74, 1.75) | 0.78 (0.38, 1.61) | 0.75 (0.42, 1.34) | 1.18 (0.70, 2.01) |
|  |  |  | p-value | 0.10 | 0.4 | 0.6 | 0.5 | 0.5 | 0.3 | 0.5 |
| 219 | IL12B | rs2546890 | OR (95% CI) | 1.68 (1.11, 2.54) | 0.77 (0.43, 1.37) | 1.12 (0.70, 1.79) | 0.64 (0.41, 1.00) | 1.19 (0.59, 2.41) | 1.16 (0.65, 2.07) | 0.81 (0.48, 1.39) |
|  |  |  | p-value | 0.01 | 0.4 | 0.6 | 0.05 | 0.6 | 0.6 | 0.4 |
| 220 | IL13 | rs20541 | OR (95% CI) | 1.11 (0.71, 1.75) | 0.79 (0.39, 1.59) | 1.09 (0.64, 1.84) | 0.95 (0.57, 1.56) | 0.56 (0.22, 1.44) | 0.92 (0.49, 1.72) | 1.21 (0.69, 2.13) |
|  |  |  | p-value | 0.7 | 0.5 | 0.8 | 0.8 | 0.2 | 0.8 | 0.5 |
| 221 | IL15RA | rs2228059 | OR (95% CI) | 1.19 (0.79, 1.79) | 0.78 (0.44, 1.40) | 1.02 (0.64, 1.63) | 0.76 (0.49, 1.17) | 1.02 (0.50, 2.07) | 0.55 (0.31, 0.99) | 0.84 (0.49, 1.43) |
|  |  |  | p-value | 0.4 | 0.4 | 0.9 | 0.2 | 1.0 | 0.05 | 0.5 |
| 222 | IL15RA | rs2296135 | OR (95% CI) | 1.25 (0.84, 1.86) | 0.86 (0.49, 1.51) | 0.74 (0.46, 1.18) | 0.83 (0.54, 1.27) | 1.04 (0.52, 2.10) | 0.56 (0.32, 0.99) | 0.91 (0.53, 1.56) |
|  |  |  | p-value | 0.3 | 0.6 | 0.2 | 0.4 | 0.9 | 0.05 | 0.7 |
| 223 | IL15RA | rs3136618 | OR (95% CI) | 0.80 (0.54, 1.21) | 1.35 (0.75, 2.41) | 1.06 (0.66, 1.69) | 1.35 (0.87, 2.09) | 1.01 (0.50, 2.06) | 2.08 (1.14, 3.80) | 1.17 (0.68, 2.01) |
|  |  |  | p-value | 0.3 | 0.3 | 0.8 | 0.2 | 1.0 | 0.02 | 0.6 |
| 224 | IL16 | rs1131445 | OR (95% CI) | 1.01 (0.66, 1.55) | 0.85 (0.45, 1.60) | 1.46 (0.90, 2.38) | 0.93 (0.58, 1.48) | 1.16 (0.56, 2.39) | 1.33 (0.72, 2.45) | 0.93 (0.53, 1.61) |
|  |  |  | p-value | 1.0 | 0.6 | 0.1 | 0.7 | 0.7 | 0.4 | 0.8 |
| 225 | IL16 | rs4072111 | OR (95% CI) | 1.15 (0.66, 2.00) | 1.36 (0.65, 2.86) | 0.56 (0.25, 1.25) | 1.03 (0.56, 1.89) | 0.63 (0.19, 2.04) | 1.13 (0.50, 2.53) | 1.38 (0.70, 2.72) |
|  |  |  | p-value | 0.6 | 0.4 | 0.2 | 0.9 | 0.4 | 0.8 | 0.4 |
| 226 | IL17RA | rs5992628 | OR (95% CI) | 1.22 (0.84, 1.78) | 0.92 (0.53, 1.57) | 0.95 (0.61, 1.48) | 1.13 (0.76, 1.69) | 1.12 (0.58, 2.17) | 0.86 (0.51, 1.47) | 0.48 (0.28, 0.85) |
|  |  |  | p-value | 0.3 | 0.7 | 0.8 | 0.5 | 0.7 | 0.6 | 0.01 |
| 227 | IL18R1 | rs3732127 | OR (95% CI) | 1.24 (0.74, 2.08) | 1.24 (0.59, 2.58) | 1.02 (0.55, 1.90) | 0.80 (0.45, 1.44) | 0.83 (0.30, 2.31) | 1.80 (0.72, 4.53) | 1.36 (0.69, 2.70) |
|  |  |  | p-value | 0.4 | 0.6 | 0.9 | 0.5 | 0.7 | 0.2 | 0.4 |
| 228 | IL18RAP | rs1420106 | OR (95% CI) | 1.29 (0.82, 2.04) | 1.09 (0.57, 2.09) | 0.96 (0.56, 1.65) | 0.93 (0.57, 1.54) | 0.86 (0.38, 1.93) | 1.51 (0.75, 3.04) | 1.13 (0.63, 2.02) |
|  |  |  | p-value | 0.3 | 0.8 | 0.9 | 0.8 | 0.7 | 0.2 | 0.7 |
| 229 | IL19 | rs2243191 | OR (95% CI) | 1.11 (0.73, 1.68) | 0.75 (0.40, 1.42) | 1.02 (0.63, 1.64) | 1.13 (0.73, 1.76) | 0.89 (0.41, 1.93) | 1.39 (0.73, 2.63) | 1.20 (0.69, 2.10) |
|  |  |  | p-value | 0.6 | 0.4 | 0.9 | 0.6 | 0.8 | 0.3 | 0.5 |
| 230 | IL1A | rs17561 | OR (95% CI) | 1.68 (1.10, 2.56) | 1.02 (0.55, 1.88) | 0.55 (0.31, 0.99) | 1.05 (0.67, 1.67) | 0.89 (0.42, 1.90) | 1.30 (0.67, 2.49) | 0.89 (0.50, 1.57) |
|  |  |  | p-value | 0.02 | 0.9 | 0.04 | 0.8 | 0.8 | 0.4 | 0.7 |
| 231 | IL1B | rs1143627 | OR (95% CI) | 0.81 (0.53, 1.24) | 1.10 (0.61, 1.97) | 1.27 (0.79, 2.05) | 1.09 (0.70, 1.71) | 1.24 (0.61, 2.53) | 1.16 (0.65, 2.10) | 0.89 (0.51, 1.57) |
|  |  |  | p-value | 0.3 | 0.8 | 0.3 | 0.7 | 0.6 | 0.6 | 0.7 |
| 232 | IL1F10 | rs2100071 | OR (95% CI) | 0.80 (0.49, 1.31) | 0.99 (0.51, 1.95) | 0.80 (0.45, 1.42) | 1.21 (0.73, 2.00) | 1.08 (0.50, 2.35) | 1.04 (0.54, 2.00) | 1.42 (0.80, 2.54) |
|  |  |  | p-value | 0.4 | 1.0 | 0.4 | 0.5 | 0.8 | 0.9 | 0.2 |
| 233 | IL1RAPL2 | rs3764765 | OR (95% CI) | 1.03 (0.64, 1.67) | 1.85 (1.04, 3.30) | 0.74 (0.40, 1.38) | 1.09 (0.64, 1.84) | 0.37 (0.07, 2.13) | 0.84 (0.46, 1.53) | 0.60 (0.25, 1.46) |
|  |  |  | p-value | 0.9 | 0.04 | 0.3 | 0.8 | 0.3 | 0.6 | 0.3 |
| 234 | IL1RL1 | rs1041973 | OR (95% CI) | 1.06 (0.68, 1.64) | 1.08 (0.58, 2.04) | 1.31 (0.79, 2.15) | 0.84 (0.52, 1.38) | 0.70 (0.29, 1.70) | 1.02 (0.53, 1.96) | 1.00 (0.56, 1.81) |
|  |  |  | p-value | 0.8 | 0.8 | 0.3 | 0.5 | 0.4 | 0.9 | 1.0 |
| 235 | IL1RL1 | rs3771175 | OR (95% CI) | 1.03 (0.58, 1.82) | 0.89 (0.38, 2.10) | 0.98 (0.50, 1.91) | 0.86 (0.46, 1.62) | 1.22 (0.44, 3.36) | 1.49 (0.56, 3.99) | 1.63 (0.80, 3.35) |
|  |  |  | p-value | 0.9 | 0.8 | 1.0 | 0.6 | 0.7 | 0.4 | 0.2 |
| 236 | IL20RA | rs1342642 | OR (95% CI) | 1.50 (0.98, 2.29) | 0.82 (0.42, 1.58) | 1.35 (0.83, 2.19) | 0.69 (0.42, 1.15) | 1.05 (0.49, 2.27) | 1.71 (0.84, 3.48) | 0.95 (0.53, 1.71) |
|  |  |  | p-value | 0.06 | 0.5 | 0.2 | 0.2 | 0.9 | 0.1 | 0.9 |
| 237 | IL20RB | rs108858 | OR (95% CI) | 0.89 (0.61, 1.31) | 1.16 (0.68, 1.97) | 1.34 (0.87, 2.07) | 0.98 (0.66, 1.47) | 0.81 (0.41, 1.60) | 1.05 (0.61, 1.82) | 0.87 (0.53, 1.45) |
|  |  |  | p-value | 0.6 | 0.6 | 0.2 | 0.9 | 0.5 | 0.8 | 0.6 |
| 238 | IL22RA1 | rs16829204 | OR (95% CI) | 0.93 (0.58, 1.49) | 1.21 (0.66, 2.22) | 1.04 (0.62, 1.76) | 0.79 (0.47, 1.33) | 1.17 (0.55, 2.50) | 0.89 (0.48, 1.63) | 0.89 (0.47, 1.69) |
|  |  |  | p-value | 0.8 | 0.5 | 0.9 | 0.4 | 0.7 | 0.7 | 0.7 |
| 239 | IL22RA1 | rs3795299 | OR (95% CI) | 0.73 (0.48, 1.11) | 0.97 (0.54, 1.72) | 0.85 (0.53, 1.36) | 1.49 (0.98, 2.27) | 0.80 (0.39, 1.64) | 0.79 (0.45, 1.39) | 1.09 (0.66, 1.82) |
|  |  |  | p-value | 0.1 | 0.9 | 0.5 | 0.06 | 0.5 | 0.4 | 0.7 |
| 240 | IL22RA1 | rs3795300 | OR (95% CI) | 1.51 (0.98, 2.31) | 0.78 (0.42, 1.46) | 0.99 (0.60, 1.64) | 0.75 (0.47, 1.21) | 1.64 (0.80, 3.36) | 1.66 (0.87, 3.16) | 1.03 (0.60, 1.77) |
|  |  |  | p-value | 0.06 | 0.4 | 1.0 | 0.2 | 0.2 | 0.1 | 0.9 |
| 241 | IL22RA2 | rs202566 | OR (95% CI) | 1.04 (0.62, 1.76) | 0.73 (0.33, 1.62) | 1.45 (0.81, 2.59) | 1.29 (0.75, 2.20) | 0.61 (0.23, 1.62) | 1.78 (0.77, 4.12) | 1.07 (0.57, 2.01) |
|  |  |  | p-value | 0.9 | 0.4 | 0.2 | 0.4 | 0.3 | 0.2 | 0.8 |
| 242 | IL23R | rs1004819 | OR (95% CI) | 1.08 (0.71, 1.64) | 1.20 (0.67, 2.15) | 0.98 (0.60, 1.59) | 1.09 (0.70, 1.71) | 0.68 (0.31, 1.49) | 0.77 (0.44, 1.35) | 0.70 (0.39, 1.25) |
|  |  |  | p-value | 0.7 | 0.5 | 0.9 | 0.7 | 0.3 | 0.4 | 0.2 |
| 243 | IL23R | rs1358748 | OR (95% CI) | 1.33 (0.79, 2.26) | 0.96 (0.44, 2.13) | 0.58 (0.29, 1.19) | 0.74 (0.40, 1.38) | 1.11 (0.43, 2.89) | 1.42 (0.61, 3.30) | 2.05 (1.05, 4.01) |
|  |  |  | p-value | 0.3 | 0.9 | 0.1 | 0.3 | 0.8 | 0.4 | 0.04 |
| 244 | IL23R | rs1884444 | OR (95% CI) | 0.94 (0.64, 1.38) | 0.95 (0.55, 1.64) | 0.73 (0.47, 1.15) | 1.03 (0.68, 1.56) | 1.08 (0.55, 2.10) | 1.32 (0.77, 2.26) | 1.81 (1.07, 3.06) |
|  |  |  | p-value | 0.7 | 0.8 | 0.2 | 0.9 | 0.8 | 0.3 | 0.03 |
| 245 | IL23R | rs7530511 | OR (95% CI) | 1.15 (0.66, 2.00) | 0.57 (0.23, 1.42) | 0.97 (0.50, 1.85) | 0.59 (0.30, 1.14) | 1.81 (0.75, 4.41) | 0.41 (0.20, 0.82) | 0.72 (0.32, 1.62) |
|  |  |  | p-value | 0.6 | 0.2 | 0.9 | 0.1 | 0.2 | 0.01 | 0.4 |
| 246 | IL24 | rs1150253 | OR (95% CI) | 0.87 (0.58, 1.31) | 1.65 (0.94, 2.92) | 0.88 (0.55, 1.42) | 0.77 (0.50, 1.20) | 1.97 (0.96, 4.02) | 1.04 (0.59, 1.83) | 0.98 (0.57, 1.66) |
|  |  |  | p-value | 0.5 | 0.08 | 0.6 | 0.2 | 0.06 | 0.9 | 0.9 |
| 247 | IL25 | rs8014568 | OR (95% CI) | 1.42 (0.93, 2.16) | 0.80 (0.43, 1.47) | 1.00 (0.62, 1.63) | 0.87 (0.55, 1.37) | 1.45 (0.69, 3.05) | 1.39 (0.75, 2.60) | 0.82 (0.46, 1.46) |
|  |  |  | p-value | 0.1 | 0.5 | 1.0 | 0.5 | 0.3 | 0.3 | 0.5 |
| 248 | IL28RA | rs10903034 | OR (95% CI) | 0.66 (0.44, 0.99) | 1.09 (0.63, 1.89) | 1.07 (0.68, 1.69) | 1.48 (0.97, 2.26) | 0.52 (0.25, 1.09) | 0.65 (0.37, 1.12) | 0.87 (0.51, 1.48) |
|  |  |  | p-value | 0.05 | 0.7 | 0.8 | 0.07 | 0.08 | 0.1 | 0.6 |
| 249 | IL29 | rs30461 | OR (95% CI) | 1.36 (0.82, 2.23) | 0.72 (0.32, 1.65) | 0.67 (0.35, 1.26) | 0.87 (0.48, 1.55) | 1.47 (0.59, 3.66) | 0.77 (0.38, 1.56) | 1.01 (0.49, 2.10) |
|  |  |  | p-value | 0.2 | 0.4 | 0.2 | 0.6 | 0.4 | 0.5 | 1.0 |
| 250 | IL3 | rs40401 | OR (95% CI) | 0.69 (0.43, 1.12) | 1.21 (0.64, 2.27) | 1.06 (0.63, 1.77) | 1.38 (0.86, 2.20) | 0.72 (0.31, 1.68) | 1.50 (0.75, 2.99) | 1.43 (0.83, 2.46) |
|  |  |  | p-value | 0.1 | 0.6 | 0.8 | 0.2 | 0.5 | 0.3 | 0.2 |
| 251 | IL31RA | rs161704 | OR (95% CI) | 0.90 (0.59, 1.39) | 1.22 (0.67, 2.20) | 0.77 (0.46, 1.28) | 0.77 (0.48, 1.24) | 1.48 (0.73, 3.02) | 0.66 (0.37, 1.16) | 1.08 (0.62, 1.87) |
|  |  |  | p-value | 0.6 | 0.5 | 0.3 | 0.3 | 0.3 | 0.1 | 0.8 |
| 252 | IL36A | rs2305152 | OR (95% CI) | 0.93 (0.59, 1.48) | 0.81 (0.41, 1.60) | 0.90 (0.52, 1.55) | 0.92 (0.56, 1.52) | 0.96 (0.45, 2.05) | 0.55 (0.30, 1.01) | 1.03 (0.58, 1.80) |
|  |  |  | p-value | 0.8 | 0.5 | 0.7 | 0.8 | 0.9 | 0.05 | 0.9 |
| 253 | IL36A | rs895497 | OR (95% CI) | 0.85 (0.52, 1.38) | 1.47 (0.77, 2.80) | 1.16 (0.68, 1.99) | 1.30 (0.79, 2.13) | 1.54 (0.70, 3.35) | 1.93 (0.91, 4.08) | 0.73 (0.38, 1.39) |
|  |  |  | p-value | 0.5 | 0.2 | 0.6 | 0.3 | 0.3 | 0.09 | 0.3 |
| 254 | IL36G | rs7584409 | OR (95% CI) | 0.85 (0.52, 1.38) | 1.41 (0.73, 2.71) | 1.22 (0.71, 2.10) | 1.16 (0.70, 1.92) | 1.74 (0.82, 3.71) | 1.78 (0.85, 3.74) | 0.75 (0.39, 1.42) |
|  |  |  | p-value | 0.5 | 0.3 | 0.5 | 0.6 | 0.1 | 0.1 | 0.4 |
| 255 | IL36RN | rs2515402 | OR (95% CI) | 0.80 (0.53, 1.22) | 0.85 (0.47, 1.56) | 0.71 (0.43, 1.17) | 1.15 (0.74, 1.78) | 1.27 (0.63, 2.54) | 0.84 (0.47, 1.49) | 1.53 (0.91, 2.57) |
|  |  |  | p-value | 0.3 | 0.6 | 0.2 | 0.5 | 0.5 | 0.6 | 0.1 |
| 256 | IL37 | rs3811047 | OR (95% CI) | 1.44 (0.95, 2.21) | 0.75 (0.40, 1.40) | 0.91 (0.56, 1.49) | 0.80 (0.50, 1.26) | 0.65 (0.29, 1.45) | 0.78 (0.43, 1.42) | 1.07 (0.61, 1.87) |
|  |  |  | p-value | 0.09 | 0.4 | 0.7 | 0.3 | 0.3 | 0.4 | 0.8 |
| 257 | IL4R | rs1805015 | OR (95% CI) | 1.07 (0.66, 1.74) | 0.95 (0.46, 1.93) | 0.53 (0.27, 1.03) | 1.12 (0.67, 1.87) | 2.75 (1.32, 5.75) | 0.86 (0.44, 1.67) | 0.65 (0.32, 1.34) |
|  |  |  | p-value | 0.8 | 0.9 | 0.06 | 0.7 | 0.007 | 0.7 | 0.2 |
| 258 | IL4R | rs8832 | OR (95% CI) | 1.08 (0.74, 1.57) | 0.52 (0.29, 0.92) | 1.11 (0.72, 1.71) | 0.84 (0.56, 1.26) | 1.42 (0.75, 2.69) | 1.24 (0.74, 2.08) | 1.57 (0.95, 2.59) |
|  |  |  | p-value | 0.7 | 0.02 | 0.6 | 0.4 | 0.3 | 0.4 | 0.08 |
| 259 | IL5RA | rs2290610 | OR (95% CI) | 1.10 (0.75, 1.61) | 0.88 (0.50, 1.53) | 0.89 (0.57, 1.40) | 0.86 (0.56, 1.30) | 2.00 (1.03, 3.88) | 0.83 (0.49, 1.41) | 0.74 (0.44, 1.25) |
|  |  |  | p-value | 0.6 | 0.6 | 0.6 | 0.5 | 0.04 | 0.5 | 0.3 |
| 260 | IL7R | rs1494555 | OR (95% CI) | 0.81 (0.52, 1.27) | 1.14 (0.62, 2.08) | 0.95 (0.57, 1.58) | 1.21 (0.76, 1.91) | 0.90 (0.43, 1.90) | 0.86 (0.48, 1.55) | 0.96 (0.55, 1.67) |
|  |  |  | p-value | 0.4 | 0.7 | 0.8 | 0.4 | 0.8 | 0.6 | 0.9 |
| 261 | IL7R | rs1494558 | OR (95% CI) | 0.86 (0.57, 1.32) | 1.11 (0.62, 2.00) | 0.97 (0.60, 1.57) | 1.14 (0.73, 1.78) | 0.84 (0.40, 1.78) | 0.81 (0.46, 1.42) | 0.93 (0.54, 1.60) |
|  |  |  | p-value | 0.5 | 0.7 | 0.9 | 0.6 | 0.7 | 0.5 | 0.8 |
| 262 | IL7R | rs3194051 | OR (95% CI) | 0.77 (0.49, 1.21) | 0.69 (0.35, 1.36) | 1.21 (0.74, 1.97) | 0.84 (0.52, 1.35) | 2.22 (1.10, 4.45) | 1.46 (0.73, 2.89) | 1.63 (0.95, 2.79) |
|  |  |  | p-value | 0.3 | 0.3 | 0.5 | 0.5 | 0.03 | 0.3 | 0.08 |
| 263 | IL7R | rs6897932 | OR (95% CI) | 1.63 (1.05, 2.55) | 1.27 (0.68, 2.39) | 0.85 (0.49, 1.46) | 0.67 (0.40, 1.12) | 0.47 (0.19, 1.13) | 0.99 (0.53, 1.87) | 1.02 (0.58, 1.79) |
|  |  |  | p-value | 0.03 | 0.5 | 0.6 | 0.1 | 0.09 | 1.0 | 1.0 |
| 264 | INHBA | rs2237432 | OR (95% CI) | 1.37 (0.89, 2.11) | 1.19 (0.65, 2.18) | 0.99 (0.59, 1.64) | 1.02 (0.64, 1.62) | 0.59 (0.25, 1.40) | 1.48 (0.75, 2.89) | 0.81 (0.44, 1.48) |
|  |  |  | p-value | 0.1 | 0.6 | 1.0 | 0.9 | 0.2 | 0.3 | 0.5 |
| 265 | INPP5D | rs9247 | OR (95% CI) | 1.07 (0.62, 1.85) | 0.44 (0.17, 1.18) | 1.06 (0.56, 1.99) | 1.46 (0.83, 2.59) | 1.17 (0.45, 3.06) | 0.95 (0.45, 2.02) | 0.59 (0.25, 1.38) |
|  |  |  | p-value | 0.8 | 0.1 | 0.9 | 0.2 | 0.7 | 0.9 | 0.2 |
| 266 | INSL3 | rs1003887 | OR (95% CI) | 0.93 (0.61, 1.41) | 1.63 (0.91, 2.90) | 1.01 (0.62, 1.64) | 0.64 (0.40, 1.02) | 0.90 (0.44, 1.83) | 0.69 (0.39, 1.23) | 0.99 (0.58, 1.68) |
|  |  |  | p-value | 0.7 | 0.10 | 1.0 | 0.06 | 0.8 | 0.2 | 1.0 |
| 267 | IRF2 | rs3756103 | OR (95% CI) | 0.76 (0.51, 1.16) | 0.70 (0.39, 1.27) | 1.00 (0.63, 1.60) | 1.24 (0.80, 1.92) | 0.85 (0.42, 1.73) | 0.86 (0.49, 1.52) | 1.55 (0.91, 2.64) |
|  |  |  | p-value | 0.2 | 0.2 | 1.0 | 0.3 | 0.6 | 0.6 | 0.1 |
| 268 | IRF2 | rs965225 | OR (95% CI) | 1.02 (0.50, 2.06) | 1.53 (0.62, 3.77) | 0.84 (0.35, 1.99) | 0.58 (0.25, 1.37) | 0.75 (0.21, 2.66) | 0.58 (0.24, 1.40) | 1.15 (0.48, 2.74) |
|  |  |  | p-value | 1.0 | 0.4 | 0.7 | 0.2 | 0.7 | 0.2 | 0.8 |
| 269 | IRF3 | rs2304207 | OR (95% CI) | 0.88 (0.49, 1.61) | 1.42 (0.65, 3.11) | 0.72 (0.34, 1.52) | 1.18 (0.64, 2.18) | 1.03 (0.38, 2.76) | 0.90 (0.42, 1.96) | 0.86 (0.40, 1.83) |
|  |  |  | p-value | 0.7 | 0.4 | 0.4 | 0.6 | 1.0 | 0.8 | 0.7 |
| 270 | IRF8 | rs10514611 | OR (95% CI) | 1.57 (1.02, 2.42) | 1.09 (0.59, 2.01) | 1.01 (0.60, 1.69) | 0.93 (0.58, 1.51) | 0.88 (0.39, 1.98) | 1.76 (0.85, 3.64) | 0.74 (0.39, 1.41) |
|  |  |  | p-value | 0.04 | 0.8 | 1.0 | 0.8 | 0.8 | 0.1 | 0.4 |
| 271 | ITGAE | rs1716 | OR (95% CI) | 0.65 (0.41, 1.05) | 0.65 (0.33, 1.27) | 2.86 (1.71, 4.78) | 0.83 (0.51, 1.33) | 2.46 (1.22, 4.99) | 1.27 (0.67, 2.41) | 0.66 (0.36, 1.22) |
|  |  |  | p-value | 0.08 | 0.2 | 0.00006 | 0.4 | 0.01 | 0.5 | 0.2 |
| 272 | ITGAE | rs2976230 | OR (95% CI) | 1.09 (0.70, 1.70) | 1.13 (0.61, 2.11) | 0.71 (0.40, 1.24) | 0.91 (0.56, 1.47) | 0.85 (0.37, 1.98) | 0.76 (0.41, 1.44) | 1.22 (0.68, 2.18) |
|  |  |  | p-value | 0.7 | 0.7 | 0.2 | 0.7 | 0.7 | 0.4 | 0.5 |
| 273 | ITGB2 | rs2070946 | OR (95% CI) | 0.91 (0.58, 1.42) | 1.40 (0.78, 2.51) | 0.86 (0.51, 1.46) | 0.78 (0.47, 1.29) | 0.95 (0.43, 2.08) | 1.47 (0.75, 2.91) | 1.85 (1.07, 3.19) |
|  |  |  | p-value | 0.7 | 0.3 | 0.6 | 0.3 | 0.9 | 0.3 | 0.03 |
| 274 | ITGB3 | rs11079772 | OR (95% CI) | 1.22 (0.79, 1.90) | 0.77 (0.40, 1.49) | 1.40 (0.85, 2.31) | 1.13 (0.70, 1.81) | 1.17 (0.55, 2.46) | 1.27 (0.67, 2.40) | 0.49 (0.25, 0.95) |
|  |  |  | p-value | 0.4 | 0.4 | 0.2 | 0.6 | 0.7 | 0.5 | 0.03 |
| 275 | KIR2DS2 | rs2242653 | OR (95% CI) | 1.09 (0.62, 1.91) | 0.77 (0.32, 1.83) | 0.66 (0.32, 1.37) | 1.19 (0.66, 2.15) | 0.60 (0.20, 1.79) | 0.71 (0.33, 1.50) | 1.27 (0.64, 2.52) |
|  |  |  | p-value | 0.8 | 0.6 | 0.3 | 0.6 | 0.4 | 0.4 | 0.5 |
| 276 | KLRK1 | rs1841958 | OR (95% CI) | 1.23 (0.82, 1.84) | 0.82 (0.44, 1.53) | 1.11 (0.69, 1.78) | 0.71 (0.45, 1.14) | 1.31 (0.65, 2.61) | 1.34 (0.72, 2.49) | 1.35 (0.81, 2.26) |
|  |  |  | p-value | 0.3 | 0.5 | 0.7 | 0.2 | 0.4 | 0.4 | 0.2 |
| 277 | KLRK1 | rs2617170 | OR (95% CI) | 1.21 (0.81, 1.81) | 0.95 (0.52, 1.71) | 1.09 (0.68, 1.75) | 0.71 (0.45, 1.12) | 1.31 (0.66, 2.59) | 1.53 (0.83, 2.84) | 1.42 (0.85, 2.37) |
|  |  |  | p-value | 0.3 | 0.9 | 0.7 | 0.1 | 0.4 | 0.2 | 0.2 |
| 278 | LAG3 | rs870849 | OR (95% CI) | 1.03 (0.69, 1.54) | 0.66 (0.36, 1.22) | 0.93 (0.58, 1.49) | 1.47 (0.96, 2.26) | 2.76 (1.33, 5.74) | 1.38 (0.75, 2.52) | 0.57 (0.32, 1.03) |
|  |  |  | p-value | 0.9 | 0.2 | 0.8 | 0.08 | 0.007 | 0.3 | 0.06 |
| 279 | LECT2 | rs31517 | OR (95% CI) | 1.09 (0.72, 1.64) | 0.87 (0.48, 1.58) | 1.16 (0.73, 1.86) | 0.95 (0.61, 1.49) | 0.83 (0.40, 1.74) | 1.01 (0.57, 1.78) | 0.84 (0.48, 1.47) |
|  |  |  | p-value | 0.7 | 0.7 | 0.5 | 0.8 | 0.6 | 1.0 | 0.5 |
| 280 | LGALS8 | rs1041935 | OR (95% CI) | 1.10 (0.72, 1.68) | 0.98 (0.53, 1.79) | 0.75 (0.45, 1.25) | 0.91 (0.58, 1.44) | 1.49 (0.72, 3.08) | 1.02 (0.56, 1.87) | 1.16 (0.66, 2.03) |
|  |  |  | p-value | 0.7 | 0.9 | 0.3 | 0.7 | 0.3 | 1.0 | 0.6 |
| 281 | LGALS8 | rs2243525 | OR (95% CI) | 1.28 (0.81, 2.01) | 1.00 (0.52, 1.92) | 0.63 (0.35, 1.11) | 0.72 (0.43, 1.20) | 1.00 (0.44, 2.26) | 0.62 (0.34, 1.17) | 1.32 (0.72, 2.42) |
|  |  |  | p-value | 0.3 | 1.0 | 0.1 | 0.2 | 1.0 | 0.1 | 0.4 |
| 282 | LIF | rs737812 | OR (95% CI) | 1.49 (0.96, 2.30) | 0.90 (0.47, 1.74) | 0.47 (0.26, 0.83) | 0.90 (0.55, 1.46) | 1.25 (0.61, 2.56) | 0.97 (0.53, 1.79) | 1.28 (0.75, 2.18) |
|  |  |  | p-value | 0.07 | 0.8 | 0.01 | 0.7 | 0.5 | 0.9 | 0.4 |
| 283 | LIFR | rs3110234 | OR (95% CI) | 0.87 (0.53, 1.44) | 1.37 (0.73, 2.58) | 0.99 (0.56, 1.73) | 0.76 (0.44, 1.31) | 0.78 (0.32, 1.86) | 0.87 (0.46, 1.65) | 1.17 (0.65, 2.13) |
|  |  |  | p-value | 0.6 | 0.3 | 1.0 | 0.3 | 0.6 | 0.7 | 0.6 |
| 284 | LILRA4 | rs2241384 | OR (95% CI) | 0.88 (0.52, 1.46) | 1.27 (0.64, 2.50) | 0.76 (0.41, 1.40) | 0.81 (0.46, 1.42) | 2.89 (1.36, 6.16) | 0.73 (0.37, 1.44) | 0.72 (0.35, 1.46) |
|  |  |  | p-value | 0.6 | 0.5 | 0.4 | 0.5 | 0.006 | 0.4 | 0.4 |
| 285 | LILRB1 | rs8101605 | OR (95% CI) | 0.69 (0.39, 1.24) | 1.65 (0.82, 3.31) | 1.20 (0.66, 2.19) | 1.03 (0.58, 1.84) | 1.96 (0.87, 4.42) | 2.26 (0.86, 5.92) | 0.89 (0.42, 1.85) |
|  |  |  | p-value | 0.2 | 0.2 | 0.5 | 0.9 | 0.1 | 0.10 | 0.7 |
| 286 | LILRB3 | rs448461 | OR (95% CI) | 0.94 (0.59, 1.48) | 1.12 (0.59, 2.13) | 1.14 (0.68, 1.92) | 0.96 (0.58, 1.57) | 1.42 (0.68, 2.98) | 0.98 (0.53, 1.84) | 0.73 (0.40, 1.35) |
|  |  |  | p-value | 0.8 | 0.7 | 0.6 | 0.9 | 0.4 | 1.0 | 0.3 |
| 287 | LILRB4 | rs731170 | OR (95% CI) | 1.38 (0.91, 2.10) | 0.73 (0.39, 1.37) | 0.99 (0.61, 1.62) | 0.85 (0.54, 1.35) | 1.20 (0.58, 2.48) | 1.15 (0.63, 2.09) | 0.97 (0.55, 1.70) |
|  |  |  | p-value | 0.1 | 0.3 | 1.0 | 0.5 | 0.6 | 0.6 | 0.9 |
| 288 | LIPA | rs1727 | OR (95% CI) | 0.77 (0.47, 1.27) | 0.98 (0.50, 1.92) | 1.53 (0.91, 2.58) | 1.15 (0.70, 1.88) | 0.76 (0.33, 1.77) | 1.24 (0.62, 2.47) | 0.94 (0.51, 1.72) |
|  |  |  | p-value | 0.3 | 1.0 | 0.1 | 0.6 | 0.5 | 0.5 | 0.8 |
| 289 | LIPA | rs17468739 | OR (95% CI) | 1.56 (0.89, 2.71) | 1.61 (0.78, 3.34) | 0.44 (0.18, 1.05) | 0.90 (0.48, 1.69) | 0.49 (0.15, 1.67) | 0.58 (0.28, 1.19) | 0.63 (0.27, 1.47) |
|  |  |  | p-value | 0.1 | 0.2 | 0.07 | 0.7 | 0.3 | 0.1 | 0.3 |
| 290 | LIPA | rs2070845 | OR (95% CI) | 1.20 (0.77, 1.87) | 0.98 (0.51, 1.87) | 0.70 (0.40, 1.22) | 0.99 (0.61, 1.61) | 0.44 (0.16, 1.16) | 0.57 (0.31, 1.03) | 1.07 (0.60, 1.91) |
|  |  |  | p-value | 0.4 | 1.0 | 0.2 | 1.0 | 0.10 | 0.06 | 0.8 |
| 291 | LITAF | rs4280262 | OR (95% CI) | 1.26 (0.76, 2.08) | 0.93 (0.44, 1.94) | 0.58 (0.29, 1.14) | 0.86 (0.49, 1.51) | 1.04 (0.44, 2.45) | 0.83 (0.42, 1.66) | 1.25 (0.66, 2.35) |
|  |  |  | p-value | 0.4 | 0.8 | 0.1 | 0.6 | 0.9 | 0.6 | 0.5 |
| 292 | LPAR2 | rs1054685 | OR (95% CI) | 0.74 (0.48, 1.14) | 0.69 (0.37, 1.28) | 0.97 (0.59, 1.60) | 1.39 (0.88, 2.18) | 1.39 (0.66, 2.95) | 1.07 (0.59, 1.96) | 1.25 (0.70, 2.22) |
|  |  |  | p-value | 0.2 | 0.2 | 0.9 | 0.2 | 0.4 | 0.8 | 0.5 |
| 293 | LST1 | rs3130062 | OR (95% CI) | 0.92 (0.39, 2.19) | 0.88 (0.25, 3.10) | 1.30 (0.51, 3.31) | 0.69 (0.25, 1.87) | 0.81 (0.19, 3.49) | 0.83 (0.27, 2.54) | 1.33 (0.51, 3.45) |
|  |  |  | p-value | 0.9 | 0.8 | 0.6 | 0.5 | 0.8 | 0.7 | 0.6 |
| 294 | LTBR | rs12354 | OR (95% CI) | 1.31 (0.83, 2.07) | 0.59 (0.28, 1.23) | 0.93 (0.53, 1.61) | 1.40 (0.86, 2.27) | 0.94 (0.43, 2.07) | 0.91 (0.50, 1.67) | 0.58 (0.29, 1.14) |
|  |  |  | p-value | 0.2 | 0.2 | 0.8 | 0.2 | 0.9 | 0.8 | 0.1 |
| 295 | LTF | rs1126478 | OR (95% CI) | 1.07 (0.70, 1.62) | 0.96 (0.52, 1.75) | 0.92 (0.56, 1.50) | 0.76 (0.48, 1.20) | 0.72 (0.34, 1.51) | 1.11 (0.61, 2.02) | 1.99 (1.17, 3.39) |
|  |  |  | p-value | 0.8 | 0.9 | 0.7 | 0.2 | 0.4 | 0.7 | 0.01 |
| 296 | LY75 | rs12692566 | OR (95% CI) | 0.89 (0.51, 1.58) | 0.93 (0.41, 2.08) | 1.37 (0.73, 2.56) | 1.20 (0.67, 2.16) | 0.88 (0.33, 2.34) | 0.74 (0.35, 1.57) | 0.55 (0.25, 1.24) |
|  |  |  | p-value | 0.7 | 0.9 | 0.3 | 0.5 | 0.8 | 0.4 | 0.2 |
| 297 | LY75 | rs2666986 | OR (95% CI) | 0.70 (0.44, 1.12) | 0.59 (0.29, 1.20) | 1.52 (0.94, 2.44) | 1.08 (0.68, 1.71) | 1.78 (0.89, 3.56) | 1.15 (0.61, 2.16) | 0.96 (0.54, 1.71) |
|  |  |  | p-value | 0.1 | 0.1 | 0.09 | 0.8 | 0.1 | 0.7 | 0.9 |
| 298 | LY9 | rs509749 | OR (95% CI) | 0.72 (0.48, 1.08) | 0.94 (0.53, 1.66) | 1.22 (0.76, 1.94) | 1.13 (0.73, 1.74) | 1.06 (0.53, 2.13) | 0.95 (0.54, 1.66) | 1.07 (0.64, 1.81) |
|  |  |  | p-value | 0.1 | 0.8 | 0.4 | 0.6 | 0.9 | 0.9 | 0.8 |
| 299 | LY9 | rs574610 | OR (95% CI) | 0.86 (0.58, 1.29) | 0.86 (0.49, 1.52) | 1.02 (0.64, 1.61) | 1.16 (0.76, 1.79) | 1.00 (0.49, 2.01) | 0.92 (0.53, 1.60) | 1.04 (0.62, 1.77) |
|  |  |  | p-value | 0.5 | 0.6 | 0.9 | 0.5 | 1.0 | 0.8 | 0.9 |
| 300 | LYN | rs952784 | OR (95% CI) | 1.28 (0.77, 2.11) | 0.58 (0.26, 1.32) | 1.14 (0.63, 2.05) | 0.92 (0.53, 1.60) | 0.71 (0.28, 1.83) | 1.16 (0.55, 2.44) | 1.21 (0.64, 2.28) |
|  |  |  | p-value | 0.3 | 0.2 | 0.7 | 0.8 | 0.5 | 0.7 | 0.6 |
| 301 | LYST | rs3768072 | OR (95% CI) | 0.84 (0.55, 1.28) | 1.27 (0.73, 2.19) | 1.10 (0.69, 1.76) | 0.88 (0.57, 1.38) | 1.59 (0.79, 3.19) | 1.25 (0.68, 2.29) | 0.89 (0.49, 1.61) |
|  |  |  | p-value | 0.4 | 0.4 | 0.7 | 0.6 | 0.2 | 0.5 | 0.7 |
| 302 | MALT1 | rs1059442 | OR (95% CI) | 0.81 (0.46, 1.43) | 1.37 (0.65, 2.89) | 0.85 (0.45, 1.62) | 0.97 (0.54, 1.77) | 1.31 (0.50, 3.43) | 1.19 (0.51, 2.77) | 1.41 (0.69, 2.87) |
|  |  |  | p-value | 0.5 | 0.4 | 0.6 | 0.9 | 0.6 | 0.7 | 0.3 |
| 303 | MALT1 | rs7506506 | OR (95% CI) | 1.35 (0.76, 2.41) | 1.02 (0.44, 2.37) | 0.49 (0.21, 1.13) | 1.22 (0.66, 2.25) | 1.19 (0.45, 3.12) | 1.12 (0.46, 2.72) | 0.97 (0.45, 2.07) |
|  |  |  | p-value | 0.3 | 1.0 | 0.09 | 0.5 | 0.7 | 0.8 | 0.9 |
| 304 | MAP3K6 | rs1138294 | OR (95% CI) | 1.16 (0.77, 1.73) | 1.50 (0.86, 2.60) | 0.81 (0.50, 1.33) | 0.87 (0.56, 1.37) | 0.83 (0.40, 1.72) | 1.07 (0.61, 1.88) | 1.03 (0.60, 1.77) |
|  |  |  | p-value | 0.5 | 0.2 | 0.4 | 0.6 | 0.6 | 0.8 | 0.9 |
| 305 | MAPK13 | rs2071863 | OR (95% CI) | 1.05 (0.64, 1.71) | 1.11 (0.56, 2.21) | 0.82 (0.45, 1.50) | 0.99 (0.59, 1.68) | 0.69 (0.26, 1.86) | 0.84 (0.42, 1.69) | 1.01 (0.52, 1.96) |
|  |  |  | p-value | 0.9 | 0.8 | 0.5 | 1.0 | 0.5 | 0.6 | 1.0 |
| 306 | MAPK9 | rs9605 | OR (95% CI) | 1.41 (0.97, 2.06) | 0.62 (0.35, 1.11) | 0.90 (0.58, 1.39) | 0.76 (0.50, 1.16) | 0.92 (0.47, 1.81) | 0.69 (0.41, 1.16) | 1.10 (0.67, 1.81) |
|  |  |  | p-value | 0.07 | 0.1 | 0.6 | 0.2 | 0.8 | 0.2 | 0.7 |
| 307 | MASP2 | rs1033638 | OR (95% CI) | 0.57 (0.34, 0.95) | 0.87 (0.44, 1.72) | 1.05 (0.61, 1.80) | 1.76 (1.08, 2.86) | 0.48 (0.16, 1.40) | 1.20 (0.58, 2.47) | 1.78 (0.97, 3.27) |
|  |  |  | p-value | 0.03 | 0.7 | 0.9 | 0.02 | 0.2 | 0.6 | 0.06 |
| 308 | MBL2 | rs2506 | OR (95% CI) | 0.82 (0.52, 1.29) | 1.64 (0.88, 3.04) | 0.81 (0.48, 1.37) | 1.01 (0.62, 1.64) | 0.58 (0.22, 1.54) | 1.07 (0.56, 2.07) | 1.50 (0.83, 2.73) |
|  |  |  | p-value | 0.4 | 0.1 | 0.4 | 1.0 | 0.3 | 0.8 | 0.2 |
| 309 | MBL2 | rs930507 | OR (95% CI) | 1.47 (0.94, 2.30) | 1.00 (0.50, 1.97) | 0.84 (0.47, 1.49) | 1.22 (0.75, 2.00) | 0.26 (0.06, 1.07) | 1.35 (0.64, 2.82) | 0.90 (0.48, 1.70) |
|  |  |  | p-value | 0.09 | 1.0 | 0.5 | 0.4 | 0.06 | 0.4 | 0.7 |
| 310 | MERTK | rs13027171 | OR (95% CI) | 1.16 (0.74, 1.82) | 0.98 (0.52, 1.86) | 1.58 (0.96, 2.62) | 0.56 (0.33, 0.98) | 0.90 (0.42, 1.94) | 0.77 (0.43, 1.40) | 0.67 (0.36, 1.26) |
|  |  |  | p-value | 0.5 | 1.0 | 0.07 | 0.04 | 0.8 | 0.4 | 0.2 |
| 311 | METTL1 | rs703842 | OR (95% CI) | 0.84 (0.55, 1.29) | 0.69 (0.36, 1.32) | 1.13 (0.70, 1.83) | 0.87 (0.55, 1.37) | 1.02 (0.50, 2.10) | 1.10 (0.61, 2.00) | 1.82 (1.08, 3.09) |
|  |  |  | p-value | 0.4 | 0.3 | 0.6 | 0.5 | 0.9 | 0.7 | 0.03 |
| 312 | MICA | rs1051792 | OR (95% CI) | 0.99 (0.66, 1.49) | 1.48 (0.85, 2.60) | 0.82 (0.50, 1.32) | 1.18 (0.77, 1.82) | 1.39 (0.67, 2.86) | 1.60 (0.86, 2.98) | 0.92 (0.53, 1.61) |
|  |  |  | p-value | 1.0 | 0.2 | 0.4 | 0.4 | 0.4 | 0.1 | 0.8 |
| 313 | MLN | rs2281820 | OR (95% CI) | 0.98 (0.67, 1.43) | 1.38 (0.81, 2.34) | 1.12 (0.73, 1.73) | 0.83 (0.55, 1.26) | 1.04 (0.54, 2.02) | 1.16 (0.68, 1.98) | 1.00 (0.60, 1.66) |
|  |  |  | p-value | 0.9 | 0.2 | 0.6 | 0.4 | 0.9 | 0.6 | 1.0 |
| 314 | MMD | rs2286806 | OR (95% CI) | 0.66 (0.44, 0.98) | 1.49 (0.86, 2.60) | 0.97 (0.62, 1.51) | 0.82 (0.54, 1.24) | 1.44 (0.73, 2.81) | 0.99 (0.57, 1.70) | 1.71 (1.02, 2.88) |
|  |  |  | p-value | 0.04 | 0.2 | 0.9 | 0.4 | 0.3 | 1.0 | 0.04 |
| 315 | MMD | rs4476230 | OR (95% CI) | 1.29 (0.84, 1.98) | 0.82 (0.44, 1.51) | 1.28 (0.78, 2.09) | 1.04 (0.66, 1.65) | 0.87 (0.42, 1.79) | 0.90 (0.51, 1.62) | 0.47 (0.26, 0.85) |
|  |  |  | p-value | 0.2 | 0.5 | 0.3 | 0.9 | 0.7 | 0.7 | 0.01 |
| 316 | MMP1 | rs17293642 | OR (95% CI) | 2.17 (1.25, 3.77) | 0.59 (0.23, 1.55) | 0.85 (0.42, 1.74) | 0.75 (0.38, 1.46) | 0.71 (0.24, 2.07) | 0.66 (0.32, 1.36) | 0.42 (0.16, 1.10) |
|  |  |  | p-value | 0.006 | 0.3 | 0.7 | 0.4 | 0.5 | 0.3 | 0.08 |
| 317 | MMP1 | rs470215^a^ | OR (95% CI) | 0.75 (0.50, 1.13) | 1.38 (0.79, 2.40) | 1.32 (0.83, 2.08) | 0.75 (0.49, 1.17) | 1.20 (0.60, 2.39) | 0.88 (0.50, 1.55) | 1.09 (0.65, 1.82) |
|  |  |  | p-value | 0.2 | 0.3 | 0.2 | 0.2 | 0.6 | 0.6 | 0.8 |
| 318 | MMP1 | rs4754880 | OR (95% CI) | 1.03 (0.64, 1.65) | 1.06 (0.54, 2.09) | 0.56 (0.29, 1.08) | 1.12 (0.68, 1.85) | 2.49 (1.22, 5.09) | 1.71 (0.77, 3.82) | 1.18 (0.64, 2.16) |
|  |  |  | p-value | 0.9 | 0.9 | 0.08 | 0.7 | 0.01 | 0.2 | 0.6 |
| 319 | MMP1 | rs7112080 | OR (95% CI) | 0.92 (0.43, 1.99) | 1.15 (0.42, 3.13) | 1.10 (0.47, 2.59) | 1.09 (0.50, 2.40) | 0.60 (0.14, 2.60) | 0.77 (0.31, 1.95) | 0.85 (0.32, 2.28) |
|  |  |  | p-value | 0.8 | 0.8 | 0.8 | 0.8 | 0.5 | 0.6 | 0.7 |
| 320 | MMP10 | rs17293348 | OR (95% CI) | 2.16 (1.24, 3.77) | 0.57 (0.22, 1.51) | 1.07 (0.54, 2.11) | 0.72 (0.37, 1.42) | 0.70 (0.23, 2.07) | 0.76 (0.36, 1.62) | 0.41 (0.15, 1.09) |
|  |  |  | p-value | 0.007 | 0.3 | 0.9 | 0.3 | 0.5 | 0.5 | 0.07 |
| 321 | MMP10 | rs3819099 | OR (95% CI) | 2.29 (1.30, 4.01) | 0.60 (0.23, 1.57) | 0.86 (0.42, 1.79) | 0.76 (0.39, 1.49) | 0.71 (0.24, 2.12) | 0.74 (0.35, 1.57) | 0.42 (0.16, 1.11) |
|  |  |  | p-value | 0.004 | 0.3 | 0.7 | 0.4 | 0.5 | 0.4 | 0.08 |
| 322 | MMP12 | rs28381705 | OR (95% CI) | 1.13 (0.59, 2.14) | 1.38 (0.60, 3.19) | 1.10 (0.52, 2.32) | 0.30 (0.11, 0.77) | 0.46 (0.10, 2.05) | 0.56 (0.24, 1.27) | 1.66 (0.75, 3.70) |
|  |  |  | p-value | 0.7 | 0.4 | 0.8 | 0.01 | 0.3 | 0.2 | 0.2 |
| 323 | MMP14 | rs3751488 | OR (95% CI) | 0.84 (0.52, 1.36) | 1.34 (0.72, 2.49) | 1.35 (0.81, 2.26) | 0.61 (0.35, 1.07) | 1.59 (0.76, 3.33) | 1.46 (0.70, 3.03) | 1.35 (0.75, 2.41) |
|  |  |  | p-value | 0.5 | 0.4 | 0.3 | 0.08 | 0.2 | 0.3 | 0.3 |
| 324 | MMP16 | rs1467251 | OR (95% CI) | 1.16 (0.71, 1.87) | 0.96 (0.47, 1.95) | 0.70 (0.38, 1.29) | 0.91 (0.54, 1.56) | 1.53 (0.69, 3.36) | 1.34 (0.63, 2.85) | 1.35 (0.73, 2.51) |
|  |  |  | p-value | 0.6 | 0.9 | 0.3 | 0.7 | 0.3 | 0.4 | 0.3 |
| 325 | MMP16 | rs2664347 | OR (95% CI) | 1.39 (0.87, 2.21) | 1.21 (0.63, 2.31) | 0.59 (0.32, 1.08) | 1.52 (0.93, 2.49) | 0.80 (0.34, 1.86) | 1.34 (0.67, 2.67) | 0.69 (0.36, 1.33) |
|  |  |  | p-value | 0.2 | 0.6 | 0.09 | 0.09 | 0.6 | 0.4 | 0.3 |
| 326 | MMP16 | rs2664368 | OR (95% CI) | 0.71 (0.44, 1.12) | 1.74 (1.00, 3.03) | 0.72 (0.42, 1.23) | 0.87 (0.54, 1.38) | 1.18 (0.56, 2.47) | 0.71 (0.40, 1.28) | 1.39 (0.79, 2.47) |
|  |  |  | p-value | 0.1 | 0.05 | 0.2 | 0.5 | 0.7 | 0.3 | 0.3 |
| 327 | MMP2 | rs1132896 | OR (95% CI) | 1.07 (0.72, 1.59) | 0.92 (0.51, 1.63) | 0.98 (0.61, 1.57) | 0.66 (0.42, 1.05) | 1.61 (0.85, 3.05) | 1.09 (0.62, 1.91) | 1.24 (0.76, 2.00) |
|  |  |  | p-value | 0.7 | 0.8 | 0.9 | 0.08 | 0.1 | 0.8 | 0.4 |
| 328 | MMP2 | rs2241145 | OR (95% CI) | 1.16 (0.80, 1.69) | 0.92 (0.54, 1.57) | 1.06 (0.69, 1.63) | 0.71 (0.47, 1.07) | 1.92 (0.97, 3.80) | 1.16 (0.69, 1.96) | 0.95 (0.59, 1.52) |
|  |  |  | p-value | 0.4 | 0.8 | 0.8 | 0.1 | 0.06 | 0.6 | 0.8 |
| 329 | MMP2 | rs243834 | OR (95% CI) | 0.73 (0.49, 1.08) | 1.18 (0.69, 2.03) | 0.99 (0.64, 1.54) | 1.54 (1.02, 2.32) | 0.49 (0.24, 1.01) | 0.83 (0.49, 1.41) | 1.03 (0.64, 1.67) |
|  |  |  | p-value | 0.1 | 0.5 | 1.0 | 0.04 | 0.05 | 0.5 | 0.9 |
| 330 | MMP3 | rs679620 | OR (95% CI) | 1.12 (0.75, 1.67) | 0.72 (0.40, 1.28) | 0.90 (0.56, 1.43) | 0.91 (0.59, 1.40) | 1.40 (0.70, 2.81) | 0.58 (0.32, 1.02) | 0.78 (0.46, 1.31) |
|  |  |  | p-value | 0.6 | 0.3 | 0.6 | 0.7 | 0.3 | 0.06 | 0.3 |
| 331 | MMP7 | rs10502001 | OR (95% CI) | 0.47 (0.26, 0.85) | 0.35 (0.14, 0.93) | 1.26 (0.72, 2.23) | 2.20 (1.32, 3.66) | 1.12 (0.50, 2.47) | 0.75 (0.39, 1.44) | 0.90 (0.48, 1.67) |
|  |  |  | p-value | 0.01 | 0.04 | 0.4 | 0.003 | 0.8 | 0.4 | 0.7 |
| 332 | MMP7 | rs14983 | OR (95% CI) | 0.51 (0.29, 0.91) | 0.35 (0.13, 0.92) | 1.23 (0.70, 2.17) | 2.03 (1.22, 3.39) | 1.11 (0.50, 2.47) | 0.77 (0.40, 1.48) | 0.99 (0.54, 1.81) |
|  |  |  | p-value | 0.02 | 0.03 | 0.5 | 0.006 | 0.8 | 0.4 | 1.0 |
| 333 | MMP8 | rs1940475 | OR (95% CI) | 1.38 (0.93, 2.05) | 1.24 (0.70, 2.17) | 1.09 (0.69, 1.71) | 0.83 (0.54, 1.28) | 0.66 (0.32, 1.37) | 0.93 (0.53, 1.62) | 0.74 (0.43, 1.27) |
|  |  |  | p-value | 0.1 | 0.5 | 0.7 | 0.4 | 0.3 | 0.8 | 0.3 |
| 334 | MMP8 | rs2701993 | OR (95% CI) | 1.00 (0.68, 1.47) | 1.48 (0.85, 2.58) | 0.88 (0.56, 1.38) | 0.93 (0.61, 1.41) | 0.65 (0.32, 1.34) | 0.79 (0.46, 1.36) | 1.06 (0.63, 1.79) |
|  |  |  | p-value | 1.0 | 0.2 | 0.6 | 0.7 | 0.2 | 0.4 | 0.8 |
| 335 | MMP9 | rs17576 | OR (95% CI) | 0.98 (0.66, 1.45) | 0.64 (0.35, 1.17) | 0.75 (0.47, 1.20) | 0.97 (0.64, 1.48) | 1.27 (0.65, 2.49) | 0.63 (0.36, 1.08) | 1.22 (0.73, 2.03) |
|  |  |  | p-value | 0.9 | 0.1 | 0.2 | 0.9 | 0.5 | 0.09 | 0.5 |
| 336 | MMP9 | rs20544 | OR (95% CI) | 0.88 (0.60, 1.31) | 1.21 (0.69, 2.12) | 0.68 (0.43, 1.07) | 0.74 (0.48, 1.12) | 1.18 (0.61, 2.29) | 0.68 (0.39, 1.17) | 1.68 (1.00, 2.81) |
|  |  |  | p-value | 0.5 | 0.5 | 0.10 | 0.2 | 0.6 | 0.2 | 0.05 |
| 337 | MMP9 | rs2274755 | OR (95% CI) | 0.54 (0.27, 1.08) | 0.73 (0.28, 1.88) | 1.11 (0.55, 2.24) | 0.92 (0.47, 1.81) | 0.56 (0.16, 1.96) | 0.42 (0.20, 0.91) | 1.68 (0.80, 3.55) |
|  |  |  | p-value | 0.08 | 0.5 | 0.8 | 0.8 | 0.4 | 0.03 | 0.2 |
| 338 | MMP9 | rs3918249 | OR (95% CI) | 1.06 (0.71, 1.58) | 0.60 (0.33, 1.11) | 0.81 (0.51, 1.29) | 0.82 (0.53, 1.27) | 1.20 (0.61, 2.37) | 0.65 (0.37, 1.13) | 1.37 (0.82, 2.28) |
|  |  |  | p-value | 0.8 | 0.1 | 0.4 | 0.4 | 0.6 | 0.1 | 0.2 |
| 339 | MMP9 | rs9509 | OR (95% CI) | 1.06 (0.48, 2.38) | 2.06 (0.69, 6.11) | 1.16 (0.47, 2.84) | 0.54 (0.19, 1.53) | 1.33 (0.29, 6.16) | 2.52 (0.52, 12.28) | 1.47 (0.51, 4.28) |
|  |  |  | p-value | 0.9 | 0.2 | 0.8 | 0.2 | 0.7 | 0.3 | 0.5 |
| 340 | MTHFR | rs1537514 | OR (95% CI) | 0.59 (0.29, 1.23) | 1.09 (0.45, 2.64) | 1.20 (0.60, 2.40) | 0.93 (0.45, 1.90) | 0.89 (0.26, 3.09) | 0.88 (0.38, 2.04) | 1.46 (0.63, 3.41) |
|  |  |  | p-value | 0.2 | 0.8 | 0.6 | 0.8 | 0.9 | 0.8 | 0.4 |
| 341 | MTHFR | rs1801131 | OR (95% CI) | 1.14 (0.74, 1.77) | 1.60 (0.89, 2.88) | 1.06 (0.64, 1.75) | 0.78 (0.48, 1.26) | 0.60 (0.26, 1.40) | 1.14 (0.61, 2.15) | 0.99 (0.55, 1.78) |
|  |  |  | p-value | 0.5 | 0.1 | 0.8 | 0.3 | 0.2 | 0.7 | 1.0 |
| 342 | MTHFR | rs1801133 | OR (95% CI) | 0.85 (0.54, 1.34) | 0.75 (0.39, 1.45) | 1.00 (0.59, 1.69) | 1.10 (0.69, 1.75) | 1.75 (0.87, 3.52) | 0.75 (0.41, 1.37) | 0.81 (0.46, 1.43) |
|  |  |  | p-value | 0.5 | 0.4 | 1.0 | 0.7 | 0.1 | 0.3 | 0.5 |
| 343 | MTHFR | rs4846047 | OR (95% CI) | 1.19 (0.77, 1.84) | 1.80 (0.98, 3.32) | 1.03 (0.62, 1.72) | 0.73 (0.45, 1.19) | 0.55 (0.23, 1.30) | 1.13 (0.59, 2.17) | 1.09 (0.61, 1.96) |
|  |  |  | p-value | 0.4 | 0.06 | 0.9 | 0.2 | 0.2 | 0.7 | 0.8 |
| 344 | MTHFR | rs4846049 | OR (95% CI) | 1.21 (0.79, 1.85) | 1.78 (0.98, 3.22) | 0.99 (0.60, 1.63) | 0.71 (0.44, 1.14) | 0.61 (0.27, 1.37) | 1.07 (0.58, 1.97) | 1.00 (0.57, 1.78) |
|  |  |  | p-value | 0.4 | 0.06 | 1.0 | 0.2 | 0.2 | 0.8 | 1.0 |
| 345 | MTHFR | rs6697244 | OR (95% CI) | 0.98 (0.64, 1.49) | 1.66 (0.92, 3.01) | 1.12 (0.69, 1.81) | 0.75 (0.48, 1.18) | 0.56 (0.25, 1.22) | 1.08 (0.60, 1.96) | 1.32 (0.75, 2.31) |
|  |  |  | p-value | 0.9 | 0.09 | 0.7 | 0.2 | 0.1 | 0.8 | 0.3 |
| 346 | MTHFR | rs7538516 | OR (95% CI) | 0.99 (0.65, 1.51) | 1.70 (0.93, 3.09) | 1.05 (0.65, 1.71) | 0.76 (0.48, 1.20) | 0.56 (0.25, 1.23) | 1.06 (0.58, 1.94) | 1.33 (0.76, 2.33) |
|  |  |  | p-value | 1.0 | 0.08 | 0.8 | 0.2 | 0.1 | 0.8 | 0.3 |
| 347 | MYD88 | rs7744 | OR (95% CI) | 0.66 (0.35, 1.25) | 0.84 (0.36, 1.94) | 0.90 (0.46, 1.79) | 1.58 (0.91, 2.76) | 0.77 (0.26, 2.27) | 1.15 (0.49, 2.72) | 1.42 (0.70, 2.85) |
|  |  |  | p-value | 0.2 | 0.7 | 0.8 | 0.1 | 0.6 | 0.7 | 0.3 |
| 348 | NCF2 | rs2274064 | OR (95% CI) | 1.07 (0.71, 1.60) | 1.14 (0.64, 2.03) | 0.74 (0.46, 1.19) | 0.90 (0.59, 1.40) | 0.70 (0.34, 1.44) | 1.69 (0.93, 3.08) | 2.35 (1.34, 4.13) |
|  |  |  | p-value | 0.7 | 0.7 | 0.2 | 0.6 | 0.3 | 0.09 | 0.003 |
| 349 | NFAT5 | rs1437134 | OR (95% CI) | 1.07 (0.69, 1.67) | 0.89 (0.48, 1.65) | 0.58 (0.34, 1.01) | 0.83 (0.52, 1.33) | 1.11 (0.54, 2.30) | 0.94 (0.52, 1.72) | 1.87 (1.06, 3.29) |
|  |  |  | p-value | 0.7 | 0.7 | 0.06 | 0.4 | 0.8 | 0.9 | 0.03 |
| 350 | NFATC1 | rs7236492 | OR (95% CI) | 1.02 (0.56, 1.87) | 1.48 (0.69, 3.18) | 0.29 (0.09, 0.94) | 1.67 (0.93, 3.01) | 0.63 (0.20, 2.06) | 1.07 (0.46, 2.51) | 1.10 (0.54, 2.27) |
|  |  |  | p-value | 1.0 | 0.3 | 0.04 | 0.09 | 0.4 | 0.9 | 0.8 |
| 351 | NFATC1 | rs9518 | OR (95% CI) | 1.42 (0.91, 2.20) | 1.02 (0.53, 1.97) | 1.14 (0.68, 1.91) | 0.95 (0.58, 1.55) | 0.55 (0.21, 1.46) | 1.85 (0.87, 3.93) | 1.07 (0.59, 1.93) |
|  |  |  | p-value | 0.1 | 0.9 | 0.6 | 0.8 | 0.2 | 0.1 | 0.8 |
| 352 | NFATC3 | rs12598 | OR (95% CI) | 1.21 (0.53, 2.79) | 1.35 (0.45, 4.10) | 1.39 (0.55, 3.54) | 0.76 (0.28, 2.05) | 1.40 (0.40, 4.85) | 0.79 (0.26, 2.41) | 0.16 (0.02, 1.29) |
|  |  |  | p-value | 0.7 | 0.6 | 0.5 | 0.6 | 0.6 | 0.7 | 0.09 |
| 353 | NFATC4 | rs2295298 | OR (95% CI) | 0.91 (0.59, 1.40) | 0.76 (0.41, 1.44) | 0.72 (0.43, 1.21) | 1.07 (0.68, 1.68) | 0.93 (0.45, 1.92) | 0.44 (0.24, 0.81) | 0.98 (0.57, 1.69) |
|  |  |  | p-value | 0.7 | 0.4 | 0.2 | 0.8 | 0.8 | 0.008 | 0.9 |
| 354 | NFKBIE | rs2282151 | OR (95% CI) | 1.29 (0.77, 2.16) | 0.32 (0.11, 0.95) | 0.74 (0.38, 1.44) | 1.16 (0.66, 2.04) | 1.50 (0.64, 3.52) | 0.63 (0.32, 1.24) | 0.75 (0.36, 1.56) |
|  |  |  | p-value | 0.3 | 0.04 | 0.4 | 0.6 | 0.4 | 0.2 | 0.4 |
| 355 | NMI | rs1048135 | OR (95% CI) | 1.06 (0.71, 1.60) | 0.86 (0.49, 1.54) | 1.20 (0.75, 1.91) | 0.54 (0.34, 0.87) | 1.80 (0.91, 3.58) | 0.82 (0.47, 1.42) | 1.11 (0.66, 1.86) |
|  |  |  | p-value | 0.8 | 0.6 | 0.4 | 0.01 | 0.09 | 0.5 | 0.7 |
| 356 | NOD2 | rs3135499 | OR (95% CI) | 0.74 (0.49, 1.11) | 1.07 (0.60, 1.88) | 1.37 (0.86, 2.17) | 1.29 (0.84, 1.97) | 1.12 (0.55, 2.29) | 1.06 (0.60, 1.86) | 0.69 (0.39, 1.20) |
|  |  |  | p-value | 0.1 | 0.8 | 0.2 | 0.2 | 0.7 | 0.9 | 0.2 |
| 357 | NOD2 | rs5743291 | OR (95% CI) | 0.98 (0.51, 1.90) | 1.25 (0.53, 2.95) | 0.94 (0.43, 2.08) | 1.79 (0.96, 3.33) | 0.79 (0.25, 2.51) | 1.39 (0.50, 3.86) | 0.47 (0.17, 1.36) |
|  |  |  | p-value | 1.0 | 0.6 | 0.9 | 0.07 | 0.7 | 0.5 | 0.2 |
| 358 | none | rs10229888 | OR (95% CI) | 0.68 (0.45, 1.03) | 1.07 (0.61, 1.89) | 1.06 (0.67, 1.68) | 1.16 (0.76, 1.77) | 1.23 (0.61, 2.48) | 1.12 (0.64, 1.98) | 1.30 (0.76, 2.20) |
|  |  |  | p-value | 0.07 | 0.8 | 0.8 | 0.5 | 0.6 | 0.7 | 0.3 |
| 359 | none | rs11982525 | OR (95% CI) | 1.37 (0.84, 2.21) | 0.72 (0.33, 1.58) | 0.60 (0.31, 1.15) | 1.34 (0.79, 2.25) | 0.50 (0.17, 1.46) | 0.79 (0.41, 1.52) | 1.00 (0.53, 1.89) |
|  |  |  | p-value | 0.2 | 0.4 | 0.1 | 0.3 | 0.2 | 0.5 | 1.0 |
| 360 | none | rs12029632 | OR (95% CI) | 1.03 (0.67, 1.58) | 0.52 (0.25, 1.06) | 1.21 (0.75, 1.97) | 1.19 (0.76, 1.87) | 1.18 (0.56, 2.48) | 1.15 (0.61, 2.15) | 0.97 (0.55, 1.71) |
|  |  |  | p-value | 0.9 | 0.07 | 0.4 | 0.4 | 0.7 | 0.7 | 0.9 |
| 361 | none | rs13295258 | OR (95% CI) | 1.38 (0.88, 2.15) | 1.01 (0.53, 1.92) | 0.84 (0.49, 1.45) | 0.71 (0.43, 1.20) | 0.76 (0.32, 1.81) | 0.82 (0.43, 1.54) | 1.15 (0.63, 2.09) |
|  |  |  | p-value | 0.2 | 1.0 | 0.5 | 0.2 | 0.5 | 0.5 | 0.7 |
| 362 | none | rs7582291 | OR (95% CI) | 1.10 (0.74, 1.64) | 1.07 (0.61, 1.88) | 0.70 (0.43, 1.12) | 0.98 (0.64, 1.50) | 0.95 (0.46, 1.93) | 0.98 (0.56, 1.73) | 1.40 (0.83, 2.38) |
|  |  |  | p-value | 0.6 | 0.8 | 0.1 | 0.9 | 0.9 | 1.0 | 0.2 |
| 363 | none | rs7881148 | OR (95% CI) | 1.26 (0.88, 1.79) | 0.85 (0.49, 1.48) | 0.51 (0.30, 0.89) | 1.26 (0.85, 1.85) | 0.64 (0.29, 1.41) | 1.12 (0.68, 1.85) | 1.48 (0.94, 2.34) |
|  |  |  | p-value | 0.2 | 0.6 | 0.02 | 0.2 | 0.3 | 0.7 | 0.09 |
| 364 | NOS1 | rs816357 | OR (95% CI) | 0.99 (0.57, 1.72) | 0.88 (0.38, 2.04) | 1.84 (1.02, 3.30) | 0.81 (0.43, 1.52) | 0.45 (0.11, 1.93) | 0.84 (0.38, 1.83) | 0.74 (0.32, 1.72) |
|  |  |  | p-value | 1.0 | 0.8 | 0.04 | 0.5 | 0.3 | 0.7 | 0.5 |
| 365 | NR4A2 | rs12803 | OR (95% CI) | 1.21 (0.80, 1.84) | 0.41 (0.21, 0.80) | 0.80 (0.49, 1.31) | 1.06 (0.68, 1.65) | 1.72 (0.83, 3.57) | 0.89 (0.49, 1.61) | 1.10 (0.64, 1.90) |
|  |  |  | p-value | 0.4 | 0.009 | 0.4 | 0.8 | 0.1 | 0.7 | 0.7 |
| 366 | NR4A2 | rs834835 | OR (95% CI) | 0.93 (0.61, 1.41) | 1.61 (0.90, 2.88) | 1.25 (0.78, 2.01) | 0.76 (0.48, 1.19) | 0.88 (0.42, 1.86) | 1.08 (0.58, 1.98) | 1.05 (0.61, 1.78) |
|  |  |  | p-value | 0.7 | 0.1 | 0.4 | 0.2 | 0.7 | 0.8 | 0.9 |
| 367 | OAS1 | rs2660 | OR (95% CI) | 1.17 (0.77, 1.78) | 0.81 (0.44, 1.49) | 1.31 (0.81, 2.13) | 0.68 (0.42, 1.10) | 1.05 (0.52, 2.13) | 0.98 (0.55, 1.77) | 1.06 (0.62, 1.82) |
|  |  |  | p-value | 0.5 | 0.5 | 0.3 | 0.1 | 0.9 | 1.0 | 0.8 |
| 368 | OAS2 | rs1293764 | OR (95% CI) | 1.12 (0.73, 1.71) | 0.85 (0.46, 1.58) | 1.58 (0.97, 2.58) | 0.77 (0.48, 1.23) | 0.76 (0.35, 1.65) | 0.92 (0.51, 1.65) | 0.86 (0.49, 1.50) |
|  |  |  | p-value | 0.6 | 0.6 | 0.07 | 0.3 | 0.5 | 0.8 | 0.6 |
| 369 | OAS2 | rs13311 | OR (95% CI) | 0.86 (0.56, 1.32) | 0.80 (0.43, 1.49) | 1.09 (0.67, 1.77) | 1.12 (0.71, 1.76) | 0.85 (0.39, 1.83) | 0.85 (0.47, 1.53) | 1.06 (0.60, 1.86) |
|  |  |  | p-value | 0.5 | 0.5 | 0.7 | 0.6 | 0.7 | 0.6 | 0.8 |
| 370 | OR2B11 | rs4925663 | OR (95% CI) | 1.16 (0.80, 1.68) | 1.79 (1.05, 3.04) | 0.70 (0.44, 1.10) | 1.20 (0.80, 1.78) | 0.86 (0.45, 1.67) | 1.90 (1.05, 3.42) | 0.98 (0.60, 1.59) |
|  |  |  | p-value | 0.4 | 0.03 | 0.1 | 0.4 | 0.7 | 0.03 | 0.9 |
| 371 | PARP1 | rs1136410 | OR (95% CI) | 1.32 (0.81, 2.18) | 1.42 (0.72, 2.78) | 0.46 (0.21, 1.02) | 1.01 (0.58, 1.75) | 0.88 (0.34, 2.25) | 1.51 (0.64, 3.61) | 1.16 (0.61, 2.20) |
|  |  |  | p-value | 0.3 | 0.3 | 0.05 | 1.0 | 0.8 | 0.3 | 0.7 |
| 372 | PARP1 | rs8679 | OR (95% CI) | 0.98 (0.60, 1.62) | 0.55 (0.23, 1.32) | 1.41 (0.82, 2.45) | 1.07 (0.63, 1.81) | 0.68 (0.27, 1.70) | 0.84 (0.43, 1.64) | 0.98 (0.54, 1.78) |
|  |  |  | p-value | 0.9 | 0.2 | 0.2 | 0.8 | 0.4 | 0.6 | 0.9 |
| 373 | PAX5 | rs12552580 | OR (95% CI) | 0.67 (0.35, 1.26) | 1.24 (0.56, 2.74) | 1.58 (0.86, 2.93) | 1.36 (0.75, 2.47) | 0.86 (0.28, 2.62) | 1.58 (0.60, 4.11) | 0.70 (0.30, 1.65) |
|  |  |  | p-value | 0.2 | 0.6 | 0.1 | 0.3 | 0.8 | 0.4 | 0.4 |
| 374 | PDCD1LG2 | rs7854413 | OR (95% CI) | 1.41 (0.84, 2.39) | 2.08 (1.03, 4.21) | 0.98 (0.53, 1.83) | 0.65 (0.34, 1.27) | 0.78 (0.24, 2.56) | 2.37 (0.83, 6.77) | 0.94 (0.42, 2.09) |
|  |  |  | p-value | 0.2 | 0.04 | 1.0 | 0.2 | 0.7 | 0.1 | 0.9 |
| 375 | PDCD5 | rs10500262 | OR (95% CI) | 0.91 (0.61, 1.37) | 1.74 (0.96, 3.14) | 0.84 (0.52, 1.35) | 1.01 (0.66, 1.55) | 0.47 (0.23, 0.96) | 0.98 (0.56, 1.73) | 1.34 (0.80, 2.27) |
|  |  |  | p-value | 0.6 | 0.07 | 0.5 | 1.0 | 0.04 | 1.0 | 0.3 |
| 376 | PDCD6IP | rs1127732 | OR (95% CI) | 0.79 (0.47, 1.34) | 1.71 (0.90, 3.26) | 1.68 (0.99, 2.85) | 0.72 (0.40, 1.28) | 0.56 (0.19, 1.62) | 1.70 (0.75, 3.85) | 1.31 (0.70, 2.42) |
|  |  |  | p-value | 0.4 | 0.1 | 0.06 | 0.3 | 0.3 | 0.2 | 0.4 |
| 377 | PDF | rs1059519 | OR (95% CI) | 0.81 (0.53, 1.24) | 1.10 (0.63, 1.95) | 1.71 (1.08, 2.69) | 1.10 (0.71, 1.70) | 0.77 (0.36, 1.64) | 1.16 (0.65, 2.07) | 0.64 (0.35, 1.16) |
|  |  |  | p-value | 0.3 | 0.7 | 0.02 | 0.7 | 0.5 | 0.6 | 0.1 |
| 378 | PDF | rs6512265 | OR (95% CI) | 0.86 (0.56, 1.33) | 1.15 (0.65, 2.05) | 1.67 (1.04, 2.67) | 1.05 (0.67, 1.64) | 0.78 (0.37, 1.68) | 1.22 (0.67, 2.24) | 0.66 (0.36, 1.20) |
|  |  |  | p-value | 0.5 | 0.6 | 0.03 | 0.8 | 0.5 | 0.5 | 0.2 |
| 379 | PDGFRA | rs1547905 | OR (95% CI) | 1.29 (0.77, 2.16) | 0.54 (0.21, 1.37) | 0.85 (0.44, 1.64) | 1.42 (0.83, 2.43) | 0.88 (0.34, 2.23) | 1.27 (0.58, 2.80) | 0.94 (0.47, 1.90) |
|  |  |  | p-value | 0.3 | 0.2 | 0.6 | 0.2 | 0.8 | 0.6 | 0.9 |
| 380 | PDGFRA | rs2228230 | OR (95% CI) | 1.06 (0.65, 1.71) | 0.56 (0.25, 1.28) | 1.08 (0.62, 1.86) | 1.38 (0.84, 2.26) | 0.95 (0.40, 2.29) | 1.25 (0.61, 2.56) | 0.98 (0.50, 1.89) |
|  |  |  | p-value | 0.8 | 0.2 | 0.8 | 0.2 | 0.9 | 0.5 | 0.9 |
| 381 | PDGFRA | rs2229307 | OR (95% CI) | 1.46 (0.89, 2.41) | 0.41 (0.14, 1.17) | 0.72 (0.37, 1.40) | 1.28 (0.74, 2.22) | 1.51 (0.66, 3.45) | 1.28 (0.58, 2.84) | 0.87 (0.41, 1.82) |
|  |  |  | p-value | 0.1 | 0.09 | 0.3 | 0.4 | 0.3 | 0.5 | 0.7 |
| 382 | PDGFRA | rs6554162 | OR (95% CI) | 0.99 (0.67, 1.47) | 0.97 (0.55, 1.72) | 1.04 (0.66, 1.64) | 0.87 (0.57, 1.35) | 1.01 (0.50, 2.05) | 0.95 (0.55, 1.65) | 1.23 (0.73, 2.05) |
|  |  |  | p-value | 1.0 | 0.9 | 0.9 | 0.5 | 1.0 | 0.8 | 0.4 |
| 383 | PECAM1 | rs1050382 | OR (95% CI) | 1.07 (0.72, 1.57) | 0.88 (0.50, 1.53) | 0.69 (0.43, 1.09) | 0.85 (0.56, 1.29) | 1.24 (0.64, 2.42) | 0.43 (0.24, 0.78) | 0.92 (0.55, 1.52) |
|  |  |  | p-value | 0.7 | 0.6 | 0.1 | 0.4 | 0.5 | 0.006 | 0.7 |
| 384 | PECAM1 | rs1131012 | OR (95% CI) | 0.96 (0.65, 1.43) | 0.95 (0.54, 1.68) | 0.84 (0.53, 1.35) | 0.89 (0.58, 1.36) | 0.82 (0.41, 1.63) | 0.53 (0.30, 0.94) | 1.01 (0.61, 1.67) |
|  |  |  | p-value | 0.8 | 0.9 | 0.5 | 0.6 | 0.6 | 0.03 | 1.0 |
| 385 | PGLYRP2 | rs892145 | OR (95% CI) | 1.26 (0.83, 1.92) | 1.44 (0.79, 2.63) | 0.88 (0.54, 1.44) | 0.65 (0.41, 1.05) | 1.42 (0.68, 2.96) | 1.29 (0.70, 2.39) | 1.03 (0.59, 1.78) |
|  |  |  | p-value | 0.3 | 0.2 | 0.6 | 0.08 | 0.3 | 0.4 | 0.9 |
| 386 | PGLYRP3 | rs843971 | OR (95% CI) | 1.05 (0.69, 1.60) | 0.67 (0.36, 1.24) | 0.86 (0.53, 1.41) | 0.98 (0.63, 1.53) | 0.87 (0.41, 1.84) | 0.74 (0.42, 1.33) | 1.45 (0.83, 2.56) |
|  |  |  | p-value | 0.8 | 0.2 | 0.6 | 0.9 | 0.7 | 0.3 | 0.2 |
| 387 | PIAS4 | rs2289863 | OR (95% CI) | 1.05 (0.70, 1.57) | 1.28 (0.73, 2.24) | 1.35 (0.85, 2.15) | 0.57 (0.36, 0.90) | 1.21 (0.59, 2.47) | 0.99 (0.56, 1.74) | 1.08 (0.62, 1.86) |
|  |  |  | p-value | 0.8 | 0.4 | 0.2 | 0.02 | 0.6 | 1.0 | 0.8 |
| 388 | PIK3R1 | rs1043526 | OR (95% CI) | 1.17 (0.62, 2.18) | 1.28 (0.56, 2.96) | 0.90 (0.42, 1.94) | 0.65 (0.31, 1.36) | 0.61 (0.18, 2.10) | 0.54 (0.25, 1.17) | 0.94 (0.41, 2.13) |
|  |  |  | p-value | 0.6 | 0.6 | 0.8 | 0.3 | 0.4 | 0.1 | 0.9 |
| 389 | PIK3R1 | rs3730089 | OR (95% CI) | 0.64 (0.36, 1.12) | 0.99 (0.48, 2.06) | 1.33 (0.76, 2.33) | 1.31 (0.77, 2.22) | 1.33 (0.56, 3.13) | 1.11 (0.53, 2.35) | 0.76 (0.36, 1.62) |
|  |  |  | p-value | 0.1 | 1.0 | 0.3 | 0.3 | 0.5 | 0.8 | 0.5 |
| 390 | PILRB | rs705866 | OR (95% CI) | 1.35 (0.80, 2.28) | 0.79 (0.34, 1.83) | 0.84 (0.43, 1.66) | 0.92 (0.51, 1.67) | 1.67 (0.76, 3.67) | 0.94 (0.44, 2.00) | 0.65 (0.31, 1.35) |
|  |  |  | p-value | 0.3 | 0.6 | 0.6 | 0.8 | 0.2 | 0.9 | 0.2 |
| 391 | PIM1 | rs10507 | OR (95% CI) | 1.21 (0.82, 1.79) | 1.29 (0.75, 2.22) | 1.10 (0.70, 1.73) | 0.70 (0.44, 1.09) | 0.48 (0.20, 1.15) | 1.08 (0.61, 1.94) | 1.26 (0.76, 2.10) |
|  |  |  | p-value | 0.3 | 0.4 | 0.7 | 0.1 | 0.1 | 0.8 | 0.4 |
| 392 | PLA2G2D | rs584367 | OR (95% CI) | 0.94 (0.62, 1.43) | 0.72 (0.39, 1.33) | 1.23 (0.75, 2.00) | 1.07 (0.68, 1.67) | 1.26 (0.63, 2.49) | 1.14 (0.63, 2.06) | 0.96 (0.57, 1.62) |
|  |  |  | p-value | 0.8 | 0.3 | 0.4 | 0.8 | 0.5 | 0.7 | 0.9 |
| 393 | PLA2G2D | rs617180 | OR (95% CI) | 1.02 (0.69, 1.53) | 0.70 (0.39, 1.25) | 0.86 (0.54, 1.38) | 0.99 (0.65, 1.52) | 0.99 (0.50, 1.95) | 0.57 (0.32, 1.00) | 0.92 (0.55, 1.54) |
|  |  |  | p-value | 0.9 | 0.2 | 0.5 | 1.0 | 1.0 | 0.05 | 0.8 |
| 394 | PLAU | rs2227551 | OR (95% CI) | 1.57 (1.03, 2.41) | 0.98 (0.52, 1.82) | 1.14 (0.69, 1.88) | 1.15 (0.73, 1.82) | 0.69 (0.30, 1.56) | 2.40 (1.13, 5.07) | 0.69 (0.37, 1.26) |
|  |  |  | p-value | 0.04 | 0.9 | 0.6 | 0.5 | 0.4 | 0.02 | 0.2 |
| 395 | PLAU | rs2227564 | OR (95% CI) | 1.52 (0.96, 2.41) | 0.91 (0.46, 1.80) | 1.17 (0.68, 2.02) | 1.13 (0.69, 1.86) | 0.84 (0.35, 2.01) | 2.03 (0.91, 4.52) | 0.59 (0.29, 1.19) |
|  |  |  | p-value | 0.07 | 0.8 | 0.6 | 0.6 | 0.7 | 0.08 | 0.1 |
| 396 | PLAU | rs4065 | OR (95% CI) | 1.45 (0.96, 2.17) | 1.05 (0.59, 1.86) | 1.26 (0.79, 2.01) | 1.24 (0.81, 1.91) | 1.02 (0.51, 2.05) | 2.65 (1.37, 5.13) | 0.55 (0.31, 0.97) |
|  |  |  | p-value | 0.08 | 0.9 | 0.3 | 0.3 | 0.9 | 0.004 | 0.04 |
| 397 | PLRG1 | rs12641958 | OR (95% CI) | 0.88 (0.57, 1.36) | 1.48 (0.84, 2.62) | 0.74 (0.44, 1.24) | 0.93 (0.59, 1.47) | 0.90 (0.43, 1.88) | 0.65 (0.37, 1.14) | 0.95 (0.54, 1.67) |
|  |  |  | p-value | 0.6 | 0.2 | 0.3 | 0.8 | 0.8 | 0.1 | 0.9 |
| 398 | PLXNC1 | rs2291326 | OR (95% CI) | 1.20 (0.75, 1.92) | 0.60 (0.27, 1.31) | 1.14 (0.66, 1.96) | 1.04 (0.62, 1.74) | 0.90 (0.38, 2.13) | 0.79 (0.42, 1.50) | 0.71 (0.36, 1.41) |
|  |  |  | p-value | 0.5 | 0.2 | 0.6 | 0.9 | 0.8 | 0.5 | 0.3 |
| 399 | PLXNC1 | rs2365736 | OR (95% CI) | 1.06 (0.72, 1.56) | 0.72 (0.40, 1.29) | 1.03 (0.66, 1.61) | 1.28 (0.85, 1.92) | 1.68 (0.88, 3.19) | 1.66 (0.91, 3.03) | 0.89 (0.53, 1.48) |
|  |  |  | p-value | 0.8 | 0.3 | 0.9 | 0.2 | 0.1 | 0.1 | 0.7 |
| 400 | PNO1 | rs2044693 | OR (95% CI) | 1.14 (0.73, 1.77) | 0.61 (0.31, 1.21) | 1.18 (0.71, 1.97) | 0.83 (0.51, 1.35) | 1.38 (0.67, 2.81) | 0.96 (0.52, 1.77) | 1.04 (0.59, 1.83) |
|  |  |  | p-value | 0.6 | 0.2 | 0.5 | 0.5 | 0.4 | 0.9 | 0.9 |
| 401 | PPARA | rs6008259 | OR (95% CI) | 1.09 (0.70, 1.71) | 1.21 (0.65, 2.26) | 1.15 (0.69, 1.90) | 0.74 (0.44, 1.23) | 0.96 (0.42, 2.21) | 1.93 (0.92, 4.04) | 1.36 (0.75, 2.47) |
|  |  |  | p-value | 0.7 | 0.6 | 0.6 | 0.2 | 0.9 | 0.08 | 0.3 |
| 402 | PPARGC1B | rs7732671 | OR (95% CI) | 1.37 (0.72, 2.62) | 0.61 (0.20, 1.84) | 0.42 (0.16, 1.12) | 2.22 (1.15, 4.26) | 0.83 (0.23, 3.00) | 2.03 (0.59, 7.02) | 0.96 (0.39, 2.39) |
|  |  |  | p-value | 0.3 | 0.4 | 0.08 | 0.02 | 0.8 | 0.3 | 0.9 |
| 403 | PPIC | rs451195 | OR (95% CI) | 0.77 (0.47, 1.26) | 0.56 (0.27, 1.17) | 0.94 (0.54, 1.65) | 1.09 (0.65, 1.82) | 0.83 (0.37, 1.84) | 0.60 (0.32, 1.12) | 1.57 (0.88, 2.82) |
|  |  |  | p-value | 0.3 | 0.1 | 0.8 | 0.7 | 0.6 | 0.1 | 0.1 |
| 404 | PPIE | rs562056 | OR (95% CI) | 0.94 (0.53, 1.70) | 1.17 (0.53, 2.56) | 1.42 (0.75, 2.68) | 1.07 (0.58, 1.97) | 0.86 (0.32, 2.30) | 1.31 (0.55, 3.09) | 0.77 (0.36, 1.65) |
|  |  |  | p-value | 0.8 | 0.7 | 0.3 | 0.8 | 0.8 | 0.5 | 0.5 |
| 405 | PPP1R15A | rs610308 | OR (95% CI) | 1.06 (0.70, 1.62) | 1.30 (0.72, 2.34) | 0.99 (0.61, 1.62) | 0.79 (0.50, 1.25) | 1.20 (0.57, 2.54) | 1.09 (0.59, 2.00) | 0.97 (0.55, 1.72) |
|  |  |  | p-value | 0.8 | 0.4 | 1.0 | 0.3 | 0.6 | 0.8 | 0.9 |
| 406 | PPP1R15A | rs611251 | OR (95% CI) | 0.99 (0.58, 1.68) | 1.15 (0.55, 2.42) | 1.00 (0.55, 1.83) | 0.87 (0.49, 1.57) | 0.94 (0.34, 2.55) | 0.79 (0.38, 1.63) | 0.99 (0.48, 2.03) |
|  |  |  | p-value | 1.0 | 0.7 | 1.0 | 0.7 | 0.9 | 0.5 | 1.0 |
| 407 | PPP2R2B | rs160974 | OR (95% CI) | 1.11 (0.74, 1.67) | 0.63 (0.34, 1.15) | 1.31 (0.81, 2.10) | 0.96 (0.62, 1.49) | 0.85 (0.42, 1.72) | 0.69 (0.39, 1.22) | 0.74 (0.43, 1.27) |
|  |  |  | p-value | 0.6 | 0.1 | 0.3 | 0.9 | 0.6 | 0.2 | 0.3 |
| 408 | PPP2R2B | rs161039 | OR (95% CI) | 1.29 (0.82, 2.02) | 0.70 (0.34, 1.46) | 1.35 (0.81, 2.25) | 0.78 (0.46, 1.32) | 0.34 (0.10, 1.15) | 0.97 (0.51, 1.83) | 1.17 (0.64, 2.17) |
|  |  |  | p-value | 0.3 | 0.3 | 0.2 | 0.4 | 0.08 | 0.9 | 0.6 |
| 409 | PPP2R2B | rs249907 | OR (95% CI) | 0.94 (0.62, 1.44) | 0.76 (0.41, 1.43) | 0.85 (0.52, 1.39) | 1.28 (0.83, 1.99) | 1.58 (0.78, 3.23) | 0.71 (0.40, 1.28) | 0.67 (0.37, 1.23) |
|  |  |  | p-value | 0.8 | 0.4 | 0.5 | 0.3 | 0.2 | 0.3 | 0.2 |
| 410 | PPP3R1 | rs2029091 | OR (95% CI) | 1.04 (0.71, 1.51) | 1.03 (0.61, 1.75) | 1.15 (0.75, 1.76) | 0.90 (0.60, 1.36) | 1.00 (0.52, 1.92) | 1.47 (0.84, 2.59) | 1.20 (0.74, 1.94) |
|  |  |  | p-value | 0.8 | 0.9 | 0.5 | 0.6 | 1.0 | 0.2 | 0.5 |
| 411 | PRKCB | rs1015408 | OR (95% CI) | 0.96 (0.57, 1.63) | 1.03 (0.51, 2.11) | 1.20 (0.67, 2.15) | 1.16 (0.68, 1.99) | 0.49 (0.17, 1.47) | 1.26 (0.59, 2.69) | 1.14 (0.58, 2.27) |
|  |  |  | p-value | 0.9 | 0.9 | 0.5 | 0.6 | 0.2 | 0.5 | 0.7 |
| 412 | PRKCB | rs2239339 | OR (95% CI) | 0.81 (0.44, 1.48) | 0.86 (0.37, 1.97) | 1.54 (0.82, 2.89) | 1.15 (0.63, 2.09) | 0.63 (0.21, 1.89) | 1.33 (0.56, 3.14) | 1.17 (0.57, 2.40) |
|  |  |  | p-value | 0.5 | 0.7 | 0.2 | 0.7 | 0.4 | 0.5 | 0.7 |
| 413 | PRKCB | rs411103 | OR (95% CI) | 0.73 (0.50, 1.08) | 0.96 (0.56, 1.65) | 0.91 (0.58, 1.41) | 1.39 (0.92, 2.10) | 0.84 (0.42, 1.66) | 0.85 (0.50, 1.45) | 1.20 (0.72, 1.99) |
|  |  |  | p-value | 0.1 | 0.9 | 0.7 | 0.1 | 0.6 | 0.5 | 0.5 |
| 414 | PRKCE | rs6722418 | OR (95% CI) | 0.82 (0.48, 1.42) | 1.58 (0.81, 3.08) | 1.01 (0.55, 1.84) | 0.89 (0.50, 1.58) | 0.65 (0.22, 1.88) | 1.56 (0.66, 3.69) | 1.59 (0.85, 2.97) |
|  |  |  | p-value | 0.5 | 0.2 | 1.0 | 0.7 | 0.4 | 0.3 | 0.1 |
| 415 | PRKCE | rs7582320 | OR (95% CI) | 0.74 (0.49, 1.12) | 1.06 (0.60, 1.86) | 1.08 (0.68, 1.71) | 1.25 (0.81, 1.92) | 1.45 (0.72, 2.91) | 0.98 (0.56, 1.74) | 0.77 (0.45, 1.33) |
|  |  |  | p-value | 0.2 | 0.8 | 0.7 | 0.3 | 0.3 | 1.0 | 0.4 |
| 416 | PRKCQ | rs2236380 | OR (95% CI) | 0.87 (0.56, 1.36) | 0.72 (0.37, 1.40) | 1.44 (0.87, 2.38) | 1.20 (0.75, 1.92) | 0.90 (0.42, 1.95) | 1.41 (0.74, 2.69) | 1.20 (0.69, 2.09) |
|  |  |  | p-value | 0.5 | 0.3 | 0.2 | 0.5 | 0.8 | 0.3 | 0.5 |
| 417 | PRKCZ | rs3123592 | OR (95% CI) | 1.13 (0.60, 2.11) | 0.91 (0.36, 2.26) | 1.15 (0.56, 2.36) | 0.66 (0.32, 1.39) | 0.69 (0.20, 2.40) | 0.69 (0.30, 1.60) | 1.05 (0.45, 2.45) |
|  |  |  | p-value | 0.7 | 0.8 | 0.7 | 0.3 | 0.6 | 0.4 | 0.9 |
| 418 | PTCRA | rs9471966 | OR (95% CI) | 0.90 (0.56, 1.47) | 1.45 (0.74, 2.87) | 0.88 (0.50, 1.54) | 0.68 (0.40, 1.17) | 2.05 (0.92, 4.55) | 0.98 (0.51, 1.91) | 1.24 (0.67, 2.31) |
|  |  |  | p-value | 0.7 | 0.3 | 0.6 | 0.2 | 0.08 | 1.0 | 0.5 |
| 419 | PTEN | rs701848 | OR (95% CI) | 0.95 (0.63, 1.42) | 1.11 (0.63, 1.94) | 1.14 (0.72, 1.81) | 0.88 (0.57, 1.36) | 0.74 (0.36, 1.51) | 0.84 (0.48, 1.47) | 1.01 (0.60, 1.72) |
|  |  |  | p-value | 0.8 | 0.7 | 0.6 | 0.6 | 0.4 | 0.5 | 1.0 |
| 420 | PTGER3 | rs5693 | OR (95% CI) | 0.82 (0.50, 1.33) | 1.45 (0.74, 2.83) | 1.09 (0.63, 1.88) | 1.26 (0.75, 2.10) | 0.90 (0.38, 2.11) | 1.15 (0.59, 2.26) | 0.73 (0.38, 1.41) |
|  |  |  | p-value | 0.4 | 0.3 | 0.8 | 0.4 | 0.8 | 0.7 | 0.4 |
| 421 | PTGER3 | rs959 | OR (95% CI) | 0.90 (0.56, 1.44) | 0.71 (0.35, 1.46) | 0.61 (0.33, 1.12) | 1.99 (1.25, 3.17) | 0.91 (0.40, 2.10) | 1.18 (0.60, 2.32) | 1.07 (0.58, 1.97) |
|  |  |  | p-value | 0.7 | 0.4 | 0.1 | 0.004 | 0.8 | 0.6 | 0.8 |
| 422 | PTGER4 | rs4957343 | OR (95% CI) | 0.90 (0.57, 1.41) | 0.77 (0.40, 1.47) | 0.94 (0.55, 1.58) | 1.52 (0.95, 2.43) | 0.46 (0.21, 1.05) | 0.83 (0.45, 1.53) | 1.11 (0.64, 1.92) |
|  |  |  | p-value | 0.6 | 0.4 | 0.8 | 0.08 | 0.06 | 0.5 | 0.7 |
| 423 | PTGIS | rs4647 | OR (95% CI) | 0.60 (0.35, 1.02) | 0.87 (0.43, 1.75) | 1.10 (0.64, 1.90) | 1.30 (0.79, 2.13) | 2.01 (0.99, 4.10) | 1.22 (0.61, 2.44) | 1.06 (0.58, 1.92) |
|  |  |  | p-value | 0.06 | 0.7 | 0.7 | 0.3 | 0.05 | 0.6 | 0.9 |
| 424 | PTGIS | rs5600 | OR (95% CI) | 0.61 (0.36, 1.03) | 0.99 (0.50, 1.98) | 1.03 (0.60, 1.79) | 1.23 (0.75, 2.02) | 1.94 (0.94, 3.98) | 1.29 (0.64, 2.58) | 1.18 (0.66, 2.12) |
|  |  |  | p-value | 0.06 | 1.0 | 0.9 | 0.4 | 0.07 | 0.5 | 0.6 |
| 425 | PTK2B | rs751019 | OR (95% CI) | 0.75 (0.49, 1.13) | 0.98 (0.55, 1.74) | 1.34 (0.83, 2.17) | 0.88 (0.57, 1.35) | 1.50 (0.76, 2.96) | 1.07 (0.60, 1.91) | 1.24 (0.75, 2.07) |
|  |  |  | p-value | 0.2 | 0.9 | 0.2 | 0.6 | 0.2 | 0.8 | 0.4 |
| 426 | PTK2B | rs939269 | OR (95% CI) | 0.92 (0.60, 1.40) | 0.71 (0.37, 1.35) | 0.65 (0.39, 1.10) | 1.37 (0.89, 2.13) | 1.02 (0.48, 2.15) | 1.01 (0.55, 1.85) | 1.39 (0.81, 2.38) |
|  |  |  | p-value | 0.7 | 0.3 | 0.1 | 0.2 | 1.0 | 1.0 | 0.2 |
| 427 | PTPN12 | rs3750050 | OR (95% CI) | 0.76 (0.46, 1.26) | 0.80 (0.39, 1.65) | 0.63 (0.34, 1.16) | 2.01 (1.25, 3.25) | 1.03 (0.43, 2.50) | 1.73 (0.77, 3.89) | 1.43 (0.77, 2.66) |
|  |  |  | p-value | 0.3 | 0.6 | 0.1 | 0.004 | 0.9 | 0.2 | 0.3 |
| 428 | PTPN12 | rs9640663 | OR (95% CI) | 1.24 (0.83, 1.87) | 0.90 (0.50, 1.61) | 1.54 (0.97, 2.47) | 0.73 (0.46, 1.14) | 1.06 (0.54, 2.10) | 0.90 (0.52, 1.57) | 0.61 (0.34, 1.07) |
|  |  |  | p-value | 0.3 | 0.7 | 0.07 | 0.2 | 0.9 | 0.7 | 0.08 |
| 429 | PTPN13 | rs10033029 | OR (95% CI) | 1.03 (0.52, 2.02) | 1.43 (0.61, 3.35) | 0.71 (0.29, 1.72) | 1.05 (0.52, 2.13) | 1.94 (0.77, 4.89) | 1.88 (0.57, 6.21) | 0.90 (0.36, 2.23) |
|  |  |  | p-value | 0.9 | 0.4 | 0.5 | 0.9 | 0.2 | 0.3 | 0.8 |
| 430 | PTPN13 | rs989902 | OR (95% CI) | 0.78 (0.51, 1.18) | 1.07 (0.59, 1.92) | 1.32 (0.81, 2.14) | 1.27 (0.81, 1.98) | 1.74 (0.85, 3.60) | 1.88 (1.02, 3.49) | 0.94 (0.54, 1.63) |
|  |  |  | p-value | 0.2 | 0.8 | 0.3 | 0.3 | 0.1 | 0.04 | 0.8 |
| 431 | PTPN22 | rs2476601 | OR (95% CI) | 1.45 (0.67, 3.15) | 1.01 (0.32, 3.16) | 0.82 (0.30, 2.26) | 0.52 (0.19, 1.42) | 1.06 (0.29, 3.90) | 0.73 (0.25, 2.11) | 1.09 (0.41, 2.93) |
|  |  |  | p-value | 0.3 | 1.0 | 0.7 | 0.2 | 0.9 | 0.6 | 0.9 |
| 432 | PTPN22 | rs3811021 | OR (95% CI) | 1.19 (0.71, 2.02) | 0.76 (0.33, 1.74) | 1.30 (0.72, 2.36) | 1.27 (0.73, 2.19) | 0.47 (0.14, 1.54) | 1.67 (0.69, 4.04) | 0.98 (0.49, 1.94) |
|  |  |  | p-value | 0.5 | 0.5 | 0.4 | 0.4 | 0.2 | 0.3 | 1.0 |
| 433 | PTPRB | rs2465811 | OR (95% CI) | 1.08 (0.72, 1.63) | 1.18 (0.67, 2.09) | 1.25 (0.79, 2.00) | 0.63 (0.39, 1.02) | 1.15 (0.56, 2.33) | 1.39 (0.73, 2.62) | 1.25 (0.74, 2.13) |
|  |  |  | p-value | 0.7 | 0.6 | 0.3 | 0.06 | 0.7 | 0.3 | 0.4 |
| 434 | PTPRD | rs1353983 | OR (95% CI) | 0.83 (0.56, 1.25) | 1.29 (0.74, 2.27) | 0.64 (0.39, 1.04) | 1.24 (0.81, 1.89) | 1.53 (0.79, 2.96) | 0.94 (0.54, 1.63) | 0.97 (0.58, 1.60) |
|  |  |  | p-value | 0.4 | 0.4 | 0.07 | 0.3 | 0.2 | 0.8 | 0.9 |
| 435 | PTPRD | rs1836225 | OR (95% CI) | 1.74 (1.00, 3.00) | 0.46 (0.16, 1.33) | 0.52 (0.23, 1.19) | 1.16 (0.63, 2.15) | 1.36 (0.57, 3.24) | 1.04 (0.47, 2.32) | 0.85 (0.41, 1.79) |
|  |  |  | p-value | 0.05 | 0.2 | 0.1 | 0.6 | 0.5 | 0.9 | 0.7 |
| 436 | PTPRD | rs2281747 | OR (95% CI) | 1.06 (0.61, 1.82) | 0.98 (0.44, 2.19) | 1.27 (0.69, 2.33) | 1.20 (0.68, 2.13) | 0.29 (0.07, 1.25) | 0.78 (0.37, 1.65) | 0.62 (0.28, 1.38) |
|  |  |  | p-value | 0.8 | 1.0 | 0.5 | 0.5 | 0.10 | 0.5 | 0.2 |
| 437 | PTPRE | rs7081735 | OR (95% CI) | 1.03 (0.67, 1.58) | 1.45 (0.80, 2.65) | 0.97 (0.59, 1.61) | 0.65 (0.40, 1.05) | 0.89 (0.43, 1.85) | 1.00 (0.55, 1.83) | 1.24 (0.73, 2.11) |
|  |  |  | p-value | 0.9 | 0.2 | 0.9 | 0.08 | 0.8 | 1.0 | 0.4 |
| 438 | PTPRE | rs7895103 | OR (95% CI) | 0.86 (0.53, 1.38) | 0.75 (0.37, 1.53) | 1.65 (0.98, 2.77) | 0.61 (0.36, 1.06) | 0.70 (0.29, 1.65) | 0.84 (0.44, 1.59) | 1.73 (0.99, 3.02) |
|  |  |  | p-value | 0.5 | 0.4 | 0.06 | 0.08 | 0.4 | 0.6 | 0.06 |
| 439 | PTPRG | rs12629204 | OR (95% CI) | 0.63 (0.35, 1.14) | 1.30 (0.63, 2.70) | 1.51 (0.86, 2.66) | 0.96 (0.53, 1.73) | 1.55 (0.65, 3.69) | 0.86 (0.42, 1.75) | 0.57 (0.25, 1.32) |
|  |  |  | p-value | 0.1 | 0.5 | 0.2 | 0.9 | 0.3 | 0.7 | 0.2 |
| 440 | PTPRG | rs9808938 | OR (95% CI) | 0.78 (0.52, 1.17) | 1.45 (0.85, 2.47) | 1.24 (0.80, 1.94) | 0.81 (0.52, 1.25) | 1.56 (0.80, 3.04) | 0.90 (0.52, 1.56) | 0.85 (0.49, 1.46) |
|  |  |  | p-value | 0.2 | 0.2 | 0.3 | 0.3 | 0.2 | 0.7 | 0.5 |
| 441 | PTPRJ | rs1503185 | OR (95% CI) | 1.45 (0.89, 2.35) | 0.67 (0.29, 1.52) | 0.96 (0.53, 1.72) | 0.54 (0.29, 1.03) | 1.52 (0.68, 3.41) | 0.85 (0.43, 1.68) | 0.95 (0.49, 1.87) |
|  |  |  | p-value | 0.1 | 0.3 | 0.9 | 0.06 | 0.3 | 0.6 | 0.9 |
| 442 | PTPRJ | rs1566734 | OR (95% CI) | 1.11 (0.65, 1.89) | 0.68 (0.28, 1.63) | 1.08 (0.58, 2.01) | 0.63 (0.32, 1.21) | 1.38 (0.59, 3.20) | 0.70 (0.35, 1.41) | 0.96 (0.48, 1.93) |
|  |  |  | p-value | 0.7 | 0.4 | 0.8 | 0.2 | 0.5 | 0.3 | 0.9 |
| 443 | PTPRJ | rs4752904 | OR (95% CI) | 1.20 (0.80, 1.80) | 0.70 (0.39, 1.23) | 0.90 (0.57, 1.44) | 1.01 (0.66, 1.54) | 0.95 (0.48, 1.89) | 0.79 (0.45, 1.38) | 0.95 (0.56, 1.61) |
|  |  |  | p-value | 0.4 | 0.2 | 0.7 | 1.0 | 0.9 | 0.4 | 0.8 |
| 444 | PTPRK | rs1417900 | OR (95% CI) | 1.33 (0.85, 2.07) | 0.47 (0.22, 1.03) | 0.97 (0.58, 1.64) | 0.93 (0.57, 1.50) | 1.26 (0.58, 2.76) | 0.78 (0.41, 1.49) | 0.93 (0.50, 1.72) |
|  |  |  | p-value | 0.2 | 0.06 | 0.9 | 0.8 | 0.6 | 0.5 | 0.8 |
| 445 | PTPRR | rs10879183 | OR (95% CI) | 1.35 (0.84, 2.16) | 0.64 (0.29, 1.40) | 1.19 (0.69, 2.06) | 0.94 (0.55, 1.60) | 1.76 (0.82, 3.74) | 1.05 (0.53, 2.09) | 0.49 (0.23, 1.05) |
|  |  |  | p-value | 0.2 | 0.3 | 0.5 | 0.8 | 0.1 | 0.9 | 0.07 |
| 446 | PTPRR | rs3803036 | OR (95% CI) | 0.94 (0.62, 1.44) | 2.14 (1.17, 3.90) | 1.05 (0.65, 1.71) | 0.58 (0.36, 0.95) | 1.06 (0.49, 2.31) | 1.08 (0.58, 2.01) | 1.28 (0.72, 2.26) |
|  |  |  | p-value | 0.8 | 0.01 | 0.8 | 0.03 | 0.9 | 0.8 | 0.4 |
| 447 | PTPRT | rs6065565 | OR (95% CI) | 0.82 (0.52, 1.31) | 0.93 (0.48, 1.79) | 1.00 (0.59, 1.69) | 0.97 (0.60, 1.56) | 1.33 (0.63, 2.80) | 0.90 (0.48, 1.71) | 1.10 (0.62, 1.95) |
|  |  |  | p-value | 0.4 | 0.8 | 1.0 | 0.9 | 0.4 | 0.8 | 0.7 |
| 448 | PTPRU | rs2235937 | OR (95% CI) | 1.02 (0.66, 1.59) | 0.83 (0.43, 1.59) | 0.87 (0.52, 1.47) | 0.99 (0.61, 1.58) | 1.58 (0.77, 3.24) | 1.56 (0.78, 3.11) | 1.47 (0.84, 2.58) |
|  |  |  | p-value | 0.9 | 0.6 | 0.6 | 1.0 | 0.2 | 0.2 | 0.2 |
| 449 | PTPRZ1 | rs1147504 | OR (95% CI) | 1.19 (0.80, 1.76) | 0.98 (0.56, 1.70) | 1.12 (0.71, 1.77) | 1.07 (0.71, 1.62) | 0.89 (0.45, 1.76) | 1.38 (0.78, 2.43) | 0.81 (0.48, 1.35) |
|  |  |  | p-value | 0.4 | 0.9 | 0.6 | 0.8 | 0.7 | 0.3 | 0.4 |
| 450 | PUS7L | rs1057190 | OR (95% CI) | 1.26 (0.70, 2.27) | 0.75 (0.29, 1.91) | 1.92 (1.01, 3.64) | 0.73 (0.37, 1.46) | 1.48 (0.51, 4.28) | 3.56 (0.97, 13.03) | 0.88 (0.36, 2.11) |
|  |  |  | p-value | 0.4 | 0.5 | 0.05 | 0.4 | 0.5 | 0.06 | 0.8 |
| 451 | PVRL2 | rs6859 | OR (95% CI) | 1.00 (0.67, 1.47) | 1.46 (0.83, 2.55) | 0.71 (0.44, 1.13) | 1.08 (0.71, 1.65) | 0.68 (0.33, 1.39) | 1.13 (0.65, 1.98) | 1.29 (0.78, 2.14) |
|  |  |  | p-value | 1.0 | 0.2 | 0.1 | 0.7 | 0.3 | 0.7 | 0.3 |
| 452 | RAG1 | rs2227973 | OR (95% CI) | 1.13 (0.66, 1.95) | 0.84 (0.36, 1.99) | 1.33 (0.73, 2.43) | 1.52 (0.88, 2.63) | 0.67 (0.21, 2.14) | 1.19 (0.51, 2.82) | 0.31 (0.09, 0.99) |
|  |  |  | p-value | 0.7 | 0.7 | 0.4 | 0.1 | 0.5 | 0.7 | 0.05 |
| 453 | RAG1 | rs3740955 | OR (95% CI) | 1.01 (0.67, 1.51) | 1.33 (0.75, 2.34) | 1.05 (0.66, 1.69) | 1.07 (0.70, 1.65) | 1.25 (0.63, 2.47) | 1.28 (0.71, 2.31) | 0.71 (0.41, 1.24) |
|  |  |  | p-value | 1.0 | 0.3 | 0.8 | 0.8 | 0.5 | 0.4 | 0.2 |
| 454 | S1PR2 | rs2116941 | OR (95% CI) | 0.84 (0.50, 1.42) | 0.54 (0.23, 1.31) | 1.09 (0.61, 1.93) | 1.27 (0.76, 2.14) | 1.20 (0.52, 2.77) | 0.78 (0.40, 1.53) | 0.98 (0.50, 1.90) |
|  |  |  | p-value | 0.5 | 0.2 | 0.8 | 0.4 | 0.7 | 0.5 | 0.9 |
| 455 | S1PR3 | rs1867 | OR (95% CI) | 0.99 (0.60, 1.63) | 1.11 (0.57, 2.19) | 1.05 (0.59, 1.86) | 0.96 (0.56, 1.64) | 0.60 (0.23, 1.58) | 0.96 (0.49, 1.87) | 1.16 (0.62, 2.18) |
|  |  |  | p-value | 1.0 | 0.8 | 0.9 | 0.9 | 0.3 | 0.9 | 0.6 |
| 456 | SARM1 | rs2239907 | OR (95% CI) | 1.21 (0.82, 1.78) | 0.93 (0.53, 1.61) | 1.35 (0.86, 2.12) | 0.85 (0.56, 1.29) | 0.73 (0.36, 1.45) | 1.40 (0.79, 2.46) | 0.99 (0.60, 1.65) |
|  |  |  | p-value | 0.3 | 0.8 | 0.2 | 0.5 | 0.4 | 0.2 | 1.0 |
| 457 | SARM1 | rs2239908 | OR (95% CI) | 1.26 (0.85, 1.86) | 1.02 (0.59, 1.77) | 1.09 (0.69, 1.72) | 0.96 (0.63, 1.45) | 0.75 (0.37, 1.50) | 1.50 (0.84, 2.68) | 0.98 (0.59, 1.63) |
|  |  |  | p-value | 0.3 | 0.9 | 0.7 | 0.8 | 0.4 | 0.2 | 0.9 |
| 458 | SARM1 | rs739439 | OR (95% CI) | 1.04 (0.61, 1.78) | 0.90 (0.43, 1.92) | 1.58 (0.89, 2.82) | 0.75 (0.41, 1.36) | 0.89 (0.36, 2.19) | 1.30 (0.59, 2.89) | 1.01 (0.51, 2.01) |
|  |  |  | p-value | 0.9 | 0.8 | 0.1 | 0.3 | 0.8 | 0.5 | 1.0 |
| 459 | SELE | rs5361 | OR (95% CI) | 2.12 (1.18, 3.82) | 0.49 (0.15, 1.62) | 1.16 (0.56, 2.40) | 0.73 (0.35, 1.54) | 0.80 (0.25, 2.60) | 1.72 (0.54, 5.49) | 0.78 (0.32, 1.90) |
|  |  |  | p-value | 0.01 | 0.2 | 0.7 | 0.4 | 0.7 | 0.4 | 0.6 |
| 460 | SELP | rs6131 | OR (95% CI) | 1.81 (1.18, 2.78) | 0.74 (0.36, 1.50) | 1.05 (0.62, 1.76) | 0.55 (0.31, 0.98) | 0.80 (0.34, 1.90) | 1.14 (0.59, 2.19) | 1.11 (0.62, 2.00) |
|  |  |  | p-value | 0.007 | 0.4 | 0.9 | 0.04 | 0.6 | 0.7 | 0.7 |
| 461 | SELP | rs6133 | OR (95% CI) | 1.06 (0.66, 1.70) | 0.70 (0.33, 1.48) | 1.30 (0.78, 2.18) | 0.74 (0.42, 1.28) | 1.09 (0.45, 2.66) | 1.33 (0.65, 2.73) | 1.64 (0.88, 3.07) |
|  |  |  | p-value | 0.8 | 0.3 | 0.3 | 0.3 | 0.9 | 0.4 | 0.1 |
| 462 | SERPINB2 | rs9320030 | OR (95% CI) | 0.85 (0.52, 1.39) | 1.09 (0.56, 2.13) | 1.04 (0.60, 1.81) | 1.07 (0.64, 1.77) | 0.81 (0.33, 1.99) | 0.99 (0.50, 1.98) | 1.20 (0.65, 2.23) |
|  |  |  | p-value | 0.5 | 0.8 | 0.9 | 0.8 | 0.6 | 1.0 | 0.6 |
| 463 | SERPINE1 | rs2070682 | OR (95% CI) | 1.11 (0.74, 1.68) | 0.97 (0.54, 1.74) | 0.71 (0.43, 1.15) | 1.11 (0.72, 1.72) | 1.21 (0.60, 2.44) | 0.87 (0.49, 1.54) | 0.83 (0.48, 1.42) |
|  |  |  | p-value | 0.6 | 0.9 | 0.2 | 0.6 | 0.6 | 0.6 | 0.5 |
| 464 | SH2B3 | rs3184504 | OR (95% CI) | 1.37 (0.86, 2.16) | 0.31 (0.15, 0.64) | 0.92 (0.54, 1.57) | 0.88 (0.54, 1.44) | 1.21 (0.57, 2.56) | 0.70 (0.37, 1.30) | 1.16 (0.65, 2.05) |
|  |  |  | p-value | 0.2 | 0.002 | 0.8 | 0.6 | 0.6 | 0.3 | 0.6 |
| 465 | SLAMF1 | rs2295612 | OR (95% CI) | 1.38 (0.78, 2.43) | 1.56 (0.72, 3.41) | 0.70 (0.33, 1.46) | 0.33 (0.15, 0.74) | 2.05 (0.88, 4.76) | 0.89 (0.41, 1.92) | 1.14 (0.57, 2.27) |
|  |  |  | p-value | 0.3 | 0.3 | 0.3 | 0.007 | 0.10 | 0.8 | 0.7 |
| 466 | SPINK5 | rs3777134 | OR (95% CI) | 0.93 (0.62, 1.42) | 0.99 (0.55, 1.79) | 1.20 (0.74, 1.95) | 0.79 (0.50, 1.24) | 0.88 (0.42, 1.85) | 0.81 (0.45, 1.47) | 1.08 (0.62, 1.87) |
|  |  |  | p-value | 0.7 | 1.0 | 0.5 | 0.3 | 0.7 | 0.5 | 0.8 |
| 467 | SPINK5 | rs6892205 | OR (95% CI) | 1.14 (0.76, 1.70) | 1.08 (0.62, 1.89) | 0.94 (0.59, 1.49) | 1.26 (0.82, 1.94) | 1.10 (0.55, 2.19) | 2.31 (1.26, 4.24) | 1.12 (0.66, 1.89) |
|  |  |  | p-value | 0.5 | 0.8 | 0.8 | 0.3 | 0.8 | 0.007 | 0.7 |
| 468 | STAT2 | rs2066807 | OR (95% CI) | 0.64 (0.27, 1.50) | 1.96 (0.79, 4.83) | 0.52 (0.18, 1.53) | 0.28 (0.08, 0.95) | 1.05 (0.30, 3.71) | 0.60 (0.24, 1.48) | 3.00 (1.32, 6.83) |
|  |  |  | p-value | 0.3 | 0.1 | 0.2 | 0.04 | 0.9 | 0.3 | 0.009 |
| 469 | STAT3 | rs1053023 | OR (95% CI) | 1.03 (0.64, 1.66) | 1.08 (0.54, 2.13) | 0.94 (0.53, 1.65) | 0.89 (0.53, 1.50) | 1.38 (0.62, 3.07) | 0.78 (0.40, 1.51) | 0.83 (0.43, 1.61) |
|  |  |  | p-value | 0.9 | 0.8 | 0.8 | 0.7 | 0.4 | 0.5 | 0.6 |
| 470 | STAT3 | rs3744483 | OR (95% CI) | 1.03 (0.64, 1.66) | 1.08 (0.54, 2.13) | 0.94 (0.53, 1.65) | 0.89 (0.53, 1.50) | 1.38 (0.62, 3.07) | 0.78 (0.40, 1.51) | 0.83 (0.43, 1.61) |
|  |  |  | p-value | 0.9 | 0.8 | 0.8 | 0.7 | 0.4 | 0.5 | 0.6 |
| 471 | STK17A | rs1044141 | OR (95% CI) | 1.08 (0.67, 1.74) | 0.99 (0.50, 1.95) | 1.22 (0.72, 2.09) | 0.95 (0.56, 1.59) | 0.45 (0.15, 1.30) | 1.00 (0.51, 1.95) | 0.95 (0.51, 1.80) |
|  |  |  | p-value | 0.7 | 1.0 | 0.5 | 0.8 | 0.1 | 1.0 | 0.9 |
| 472 | SULT2A1 | rs2910393 | OR (95% CI) | 0.80 (0.50, 1.30) | 1.00 (0.53, 1.88) | 1.18 (0.71, 1.98) | 1.15 (0.72, 1.84) | 0.82 (0.36, 1.87) | 0.98 (0.52, 1.85) | 0.93 (0.50, 1.70) |
|  |  |  | p-value | 0.4 | 1.0 | 0.5 | 0.6 | 0.6 | 0.9 | 0.8 |
| 473 | SYK | rs1049164 | OR (95% CI) | 0.97 (0.57, 1.64) | 1.55 (0.79, 3.01) | 1.25 (0.70, 2.24) | 0.93 (0.53, 1.64) | 0.94 (0.38, 2.31) | 1.87 (0.78, 4.49) | 0.89 (0.44, 1.80) |
|  |  |  | p-value | 0.9 | 0.2 | 0.5 | 0.8 | 0.9 | 0.2 | 0.8 |
| 474 | TAP2 | rs13501 | OR (95% CI) | 0.89 (0.60, 1.33) | 0.74 (0.42, 1.33) | 0.62 (0.38, 1.01) | 1.55 (1.02, 2.35) | 0.97 (0.49, 1.89) | 1.02 (0.59, 1.75) | 1.28 (0.78, 2.12) |
|  |  |  | p-value | 0.6 | 0.3 | 0.06 | 0.04 | 0.9 | 1.0 | 0.3 |
| 475 | TAP2 | rs241447 | OR (95% CI) | 0.85 (0.56, 1.31) | 0.86 (0.47, 1.57) | 0.58 (0.34, 0.99) | 1.55 (1.00, 2.41) | 1.29 (0.65, 2.58) | 1.34 (0.73, 2.47) | 1.34 (0.78, 2.28) |
|  |  |  | p-value | 0.5 | 0.6 | 0.05 | 0.05 | 0.5 | 0.3 | 0.3 |
| 476 | TAPBP | rs2071888 | OR (95% CI) | 1.08 (0.73, 1.60) | 1.00 (0.58, 1.75) | 0.95 (0.60, 1.51) | 1.28 (0.84, 1.95) | 1.16 (0.58, 2.31) | 0.65 (0.37, 1.15) | 0.37 (0.20, 0.67) |
|  |  |  | p-value | 0.7 | 1.0 | 0.8 | 0.2 | 0.7 | 0.1 | 0.001 |
| 477 | TBX21 | rs7502875 | OR (95% CI) | 1.22 (0.77, 1.95) | 0.78 (0.38, 1.57) | 0.87 (0.50, 1.53) | 1.13 (0.68, 1.87) | 0.90 (0.39, 2.08) | 1.19 (0.61, 2.33) | 1.08 (0.58, 2.00) |
|  |  |  | p-value | 0.4 | 0.5 | 0.6 | 0.6 | 0.8 | 0.6 | 0.8 |
| 478 | TDGF1 | rs3737002 | OR (95% CI) | 1.49 (0.96, 2.31) | 0.95 (0.49, 1.82) | 1.21 (0.72, 2.01) | 0.76 (0.45, 1.26) | 0.88 (0.40, 1.95) | 1.65 (0.81, 3.36) | 0.94 (0.53, 1.69) |
|  |  |  | p-value | 0.07 | 0.9 | 0.5 | 0.3 | 0.8 | 0.2 | 0.8 |
| 479 | TEC | rs3805184 | OR (95% CI) | 0.67 (0.43, 1.06) | 0.71 (0.37, 1.37) | 1.26 (0.79, 2.01) | 1.15 (0.74, 1.76) | 0.84 (0.39, 1.80) | 0.62 (0.36, 1.09) | 1.12 (0.66, 1.91) |
|  |  |  | p-value | 0.08 | 0.3 | 0.3 | 0.5 | 0.7 | 0.09 | 0.7 |
| 480 | TESK1 | rs2275422 | OR (95% CI) | 1.12 (0.76, 1.64) | 0.79 (0.45, 1.39) | 0.75 (0.48, 1.18) | 0.88 (0.58, 1.33) | 1.01 (0.52, 1.95) | 1.01 (0.59, 1.74) | 1.65 (1.00, 2.74) |
|  |  |  | p-value | 0.6 | 0.4 | 0.2 | 0.5 | 1.0 | 1.0 | 0.05 |
| 481 | TESK1 | rs7028425 | OR (95% CI) | 1.08 (0.72, 1.61) | 1.01 (0.57, 1.79) | 0.63 (0.38, 1.03) | 1.07 (0.70, 1.64) | 1.17 (0.60, 2.27) | 1.13 (0.63, 2.01) | 1.27 (0.77, 2.10) |
|  |  |  | p-value | 0.7 | 1.0 | 0.07 | 0.8 | 0.7 | 0.7 | 0.4 |
| 482 | TGFB1 | rs1800471 | OR (95% CI) | 0.82 (0.35, 1.93) | 1.29 (0.43, 3.85) | 2.30 (1.03, 5.11) | 0.71 (0.26, 1.89) | 0.89 (0.20, 4.00) | 1.30 (0.38, 4.43) | 0.61 (0.18, 2.15) |
|  |  |  | p-value | 0.6 | 0.7 | 0.04 | 0.5 | 0.9 | 0.7 | 0.4 |
| 483 | TGFB1 | rs2241718 | OR (95% CI) | 0.90 (0.52, 1.54) | 0.85 (0.38, 1.89) | 0.46 (0.21, 0.99) | 1.06 (0.61, 1.86) | 1.98 (0.92, 4.25) | 0.61 (0.31, 1.20) | 1.02 (0.53, 1.96) |
|  |  |  | p-value | 0.7 | 0.7 | 0.05 | 0.8 | 0.08 | 0.2 | 1.0 |
| 484 | TGFB1 | rs2241720 | OR (95% CI) | 0.92 (0.54, 1.58) | 0.97 (0.45, 2.09) | 0.43 (0.20, 0.94) | 1.03 (0.59, 1.80) | 1.96 (0.91, 4.22) | 0.63 (0.32, 1.23) | 1.00 (0.52, 1.93) |
|  |  |  | p-value | 0.8 | 0.9 | 0.04 | 0.9 | 0.09 | 0.2 | 1.0 |
| 485 | TGFB1 | rs6957 | OR (95% CI) | 0.84 (0.50, 1.40) | 0.94 (0.45, 1.97) | 0.48 (0.24, 0.96) | 1.04 (0.61, 1.77) | 1.84 (0.86, 3.96) | 0.60 (0.32, 1.14) | 1.21 (0.65, 2.25) |
|  |  |  | p-value | 0.5 | 0.9 | 0.04 | 0.9 | 0.1 | 0.1 | 0.5 |
| 486 | TGFB1 | rs723877 | OR (95% CI) | 1.02 (0.68, 1.54) | 0.86 (0.48, 1.57) | 1.34 (0.84, 2.14) | 0.89 (0.57, 1.38) | 0.80 (0.38, 1.68) | 0.76 (0.42, 1.38) | 0.88 (0.51, 1.51) |
|  |  |  | p-value | 0.9 | 0.6 | 0.2 | 0.6 | 0.5 | 0.4 | 0.6 |
| 487 | TGFB2 | rs2000220 | OR (95% CI) | 0.68 (0.44, 1.06) | 1.23 (0.67, 2.23) | 1.21 (0.74, 2.00) | 1.16 (0.73, 1.82) | 1.05 (0.50, 2.20) | 0.99 (0.55, 1.80) | 0.95 (0.54, 1.68) |
|  |  |  | p-value | 0.09 | 0.5 | 0.4 | 0.5 | 0.9 | 1.0 | 0.9 |
| 488 | TGFB2 | rs2796820 | OR (95% CI) | 1.66 (1.02, 2.71) | 0.60 (0.27, 1.36) | 0.96 (0.53, 1.73) | 0.75 (0.42, 1.33) | 0.42 (0.14, 1.26) | 0.81 (0.41, 1.61) | 1.07 (0.56, 2.05) |
|  |  |  | p-value | 0.04 | 0.2 | 0.9 | 0.3 | 0.1 | 0.5 | 0.8 |
| 489 | TGFB3 | rs3917201 | OR (95% CI) | 1.54 (0.98, 2.41) | 0.72 (0.36, 1.44) | 0.94 (0.55, 1.59) | 1.03 (0.63, 1.68) | 0.60 (0.24, 1.53) | 1.12 (0.58, 2.17) | 0.84 (0.45, 1.59) |
|  |  |  | p-value | 0.06 | 0.3 | 0.8 | 0.9 | 0.3 | 0.7 | 0.6 |
| 490 | TGFBR1 | rs334349 | OR (95% CI) | 0.65 (0.41, 1.02) | 0.79 (0.41, 1.51) | 0.86 (0.52, 1.43) | 1.71 (1.08, 2.70) | 0.73 (0.32, 1.66) | 0.63 (0.35, 1.11) | 1.08 (0.61, 1.91) |
|  |  |  | p-value | 0.06 | 0.5 | 0.6 | 0.02 | 0.5 | 0.1 | 0.8 |
| 491 | THPO | rs6141^a^ | OR (95% CI) | 1.00 (0.68, 1.48) | 0.85 (0.49, 1.50) | 0.97 (0.62, 1.53) | 1.12 (0.74, 1.69) | 1.14 (0.58, 2.24) | 0.93 (0.54, 1.61) | 0.86 (0.51, 1.46) |
|  |  |  | p-value | 1.0 | 0.6 | 0.9 | 0.6 | 0.7 | 0.8 | 0.6 |
| 492 | TICAM2 | rs256996 | OR (95% CI) | 1.16 (0.80, 1.69) | 1.13 (0.66, 1.92) | 0.65 (0.41, 1.02) | 1.17 (0.78, 1.75) | 0.76 (0.40, 1.46) | 0.76 (0.45, 1.28) | 0.87 (0.54, 1.42) |
|  |  |  | p-value | 0.4 | 0.7 | 0.06 | 0.4 | 0.4 | 0.3 | 0.6 |
| 493 | TLR1 | rs4833095 | OR (95% CI) | 0.84 (0.54, 1.30) | 0.99 (0.54, 1.82) | 1.47 (0.91, 2.38) | 0.86 (0.54, 1.37) | 1.16 (0.56, 2.42) | 1.10 (0.60, 2.02) | 1.08 (0.61, 1.89) |
|  |  |  | p-value | 0.4 | 1.0 | 0.1 | 0.5 | 0.7 | 0.8 | 0.8 |
| 494 | TLR10 | rs11096955 | OR (95% CI) | 1.03 (0.69, 1.54) | 1.03 (0.59, 1.79) | 1.51 (0.96, 2.38) | 0.88 (0.57, 1.34) | 0.79 (0.38, 1.66) | 1.03 (0.58, 1.81) | 0.83 (0.47, 1.44) |
|  |  |  | p-value | 0.9 | 0.9 | 0.07 | 0.5 | 0.5 | 0.9 | 0.5 |
| 495 | TLR3 | rs3775291 | OR (95% CI) | 0.79 (0.50, 1.25) | 1.17 (0.65, 2.13) | 1.12 (0.68, 1.85) | 0.92 (0.58, 1.47) | 1.14 (0.55, 2.35) | 0.70 (0.38, 1.28) | 0.84 (0.48, 1.50) |
|  |  |  | p-value | 0.3 | 0.6 | 0.7 | 0.7 | 0.7 | 0.2 | 0.6 |
| 496 | TLR5 | rs5744174 | OR (95% CI) | 1.21 (0.80, 1.83) | 0.60 (0.32, 1.11) | 1.20 (0.74, 1.93) | 0.71 (0.45, 1.12) | 1.40 (0.70, 2.78) | 0.96 (0.54, 1.71) | 1.11 (0.65, 1.90) |
|  |  |  | p-value | 0.4 | 0.1 | 0.5 | 0.1 | 0.3 | 0.9 | 0.7 |
| 497 | TLR6 | rs5743810 | OR (95% CI) | 0.96 (0.62, 1.49) | 2.25 (1.21, 4.20) | 0.70 (0.41, 1.19) | 1.01 (0.64, 1.60) | 0.88 (0.43, 1.78) | 1.05 (0.58, 1.93) | 0.83 (0.48, 1.42) |
|  |  |  | p-value | 0.9 | 0.01 | 0.2 | 1.0 | 0.7 | 0.9 | 0.5 |
| 498 | TLR7 | rs179008 | OR (95% CI) | 1.08 (0.72, 1.63) | 1.03 (0.58, 1.84) | 0.93 (0.57, 1.53) | 1.08 (0.70, 1.68) | 0.82 (0.39, 1.74) | 1.28 (0.69, 2.36) | 1.18 (0.71, 1.95) |
|  |  |  | p-value | 0.7 | 0.9 | 0.8 | 0.7 | 0.6 | 0.4 | 0.5 |
| 499 | TLR8 | rs3764880 | OR (95% CI) | 0.81 (0.57, 1.16) | 1.22 (0.76, 1.94) | 1.17 (0.80, 1.70) | 0.96 (0.66, 1.39) | 1.37 (0.78, 2.41) | 1.32 (0.81, 2.15) | 1.12 (0.71, 1.77) |
|  |  |  | p-value | 0.3 | 0.4 | 0.4 | 0.8 | 0.3 | 0.3 | 0.6 |
| 500 | TMCO6 | rs17208187 | OR (95% CI) | 1.35 (0.82, 2.20) | 1.02 (0.50, 2.08) | 0.46 (0.22, 0.94) | 0.83 (0.48, 1.45) | 1.07 (0.47, 2.44) | 0.68 (0.35, 1.34) | 1.03 (0.55, 1.93) |
|  |  |  | p-value | 0.2 | 1.0 | 0.03 | 0.5 | 0.9 | 0.3 | 0.9 |
| 501 | TNFAIP6 | rs1046668 | OR (95% CI) | 1.11 (0.65, 1.88) | 1.32 (0.64, 2.71) | 1.00 (0.54, 1.84) | 0.70 (0.37, 1.30) | 0.88 (0.32, 2.47) | 0.83 (0.40, 1.71) | 1.05 (0.50, 2.23) |
|  |  |  | p-value | 0.7 | 0.4 | 1.0 | 0.3 | 0.8 | 0.6 | 0.9 |
| 502 | TNFAIP6 | rs1046675 | OR (95% CI) | 1.29 (0.73, 2.27) | 1.56 (0.74, 3.32) | 0.88 (0.44, 1.76) | 0.64 (0.32, 1.26) | 0.69 (0.23, 2.08) | 0.82 (0.38, 1.78) | 1.03 (0.47, 2.23) |
|  |  |  | p-value | 0.4 | 0.2 | 0.7 | 0.2 | 0.5 | 0.6 | 0.9 |
| 503 | TNFRSF10A | rs2230229 | OR (95% CI) | 0.84 (0.47, 1.47) | 1.63 (0.82, 3.25) | 1.32 (0.73, 2.38) | 0.78 (0.42, 1.44) | 0.34 (0.08, 1.44) | 0.53 (0.26, 1.04) | 0.63 (0.28, 1.45) |
|  |  |  | p-value | 0.5 | 0.2 | 0.4 | 0.4 | 0.1 | 0.07 | 0.3 |
| 504 | TNFRSF10B | rs2889 | OR (95% CI) | 0.94 (0.60, 1.47) | 1.38 (0.75, 2.54) | 0.89 (0.53, 1.51) | 0.75 (0.46, 1.22) | 1.04 (0.49, 2.21) | 1.11 (0.59, 2.09) | 1.47 (0.83, 2.58) |
|  |  |  | p-value | 0.8 | 0.3 | 0.7 | 0.2 | 0.9 | 0.7 | 0.2 |
| 505 | TNFRSF11A | rs1805034 | OR (95% CI) | 0.98 (0.65, 1.47) | 0.86 (0.48, 1.55) | 1.15 (0.72, 1.84) | 1.04 (0.67, 1.61) | 0.87 (0.42, 1.79) | 1.16 (0.65, 2.06) | 1.09 (0.64, 1.88) |
|  |  |  | p-value | 0.9 | 0.6 | 0.6 | 0.9 | 0.7 | 0.6 | 0.7 |
| 506 | TNFRSF11B | rs3134054 | OR (95% CI) | 1.37 (0.89, 2.11) | 1.43 (0.78, 2.63) | 1.32 (0.80, 2.17) | 0.70 (0.44, 1.12) | 0.60 (0.27, 1.33) | 1.48 (0.78, 2.79) | 0.83 (0.46, 1.48) |
|  |  |  | p-value | 0.1 | 0.2 | 0.3 | 0.1 | 0.2 | 0.2 | 0.5 |
| 507 | TNFRSF11B | rs6469783 | OR (95% CI) | 1.21 (0.74, 1.98) | 0.85 (0.39, 1.84) | 0.61 (0.32, 1.19) | 1.09 (0.64, 1.87) | 0.56 (0.17, 1.84) | 0.87 (0.43, 1.77) | 1.25 (0.63, 2.48) |
|  |  |  | p-value | 0.5 | 0.7 | 0.1 | 0.8 | 0.3 | 0.7 | 0.5 |
| 508 | TNFRSF14 | rs2234167 | OR (95% CI) | 1.50 (0.78, 2.88) | 0.14 (0.02, 1.03) | 0.93 (0.41, 2.12) | 1.49 (0.75, 2.97) | 1.05 (0.34, 3.24) | 1.23 (0.44, 3.40) | 0.89 (0.36, 2.17) |
|  |  |  | p-value | 0.2 | 0.05 | 0.9 | 0.3 | 0.9 | 0.7 | 0.8 |
| 509 | TNFRSF19 | rs3751364 | OR (95% CI) | 0.79 (0.49, 1.26) | 1.14 (0.61, 2.12) | 1.19 (0.72, 1.97) | 0.74 (0.44, 1.22) | 1.84 (0.90, 3.79) | 1.16 (0.60, 2.24) | 1.32 (0.74, 2.35) |
|  |  |  | p-value | 0.3 | 0.7 | 0.5 | 0.2 | 0.10 | 0.7 | 0.3 |
| 510 | TRAF1 | rs4836834 | OR (95% CI) | 1.18 (0.80, 1.74) | 0.88 (0.51, 1.52) | 1.11 (0.71, 1.74) | 0.87 (0.57, 1.31) | 1.61 (0.83, 3.10) | 0.99 (0.58, 1.70) | 0.73 (0.43, 1.22) |
|  |  |  | p-value | 0.4 | 0.6 | 0.7 | 0.5 | 0.2 | 1.0 | 0.2 |
| 511 | TRAF3 | rs1131877 | OR (95% CI) | 0.94 (0.60, 1.48) | 1.22 (0.65, 2.30) | 0.57 (0.32, 1.02) | 1.51 (0.94, 2.43) | 1.02 (0.46, 2.22) | 1.03 (0.54, 1.96) | 0.99 (0.55, 1.79) |
|  |  |  | p-value | 0.8 | 0.5 | 0.06 | 0.09 | 1.0 | 0.9 | 1.0 |
| 512 | TRAF3IP2 | rs1043730 | OR (95% CI) | 0.90 (0.55, 1.49) | 1.36 (0.70, 2.62) | 0.53 (0.27, 1.04) | 0.91 (0.53, 1.55) | 0.89 (0.39, 2.06) | 0.84 (0.43, 1.63) | 1.78 (0.99, 3.21) |
|  |  |  | p-value | 0.7 | 0.4 | 0.06 | 0.7 | 0.8 | 0.6 | 0.06 |
| 513 | TRAP1 | rs13926 | OR (95% CI) | 0.86 (0.58, 1.28) | 0.93 (0.53, 1.64) | 0.87 (0.55, 1.39) | 1.12 (0.74, 1.71) | 0.76 (0.38, 1.51) | 0.68 (0.39, 1.20) | 1.11 (0.67, 1.85) |
|  |  |  | p-value | 0.5 | 0.8 | 0.6 | 0.6 | 0.4 | 0.2 | 0.7 |
| 514 | TROVE2 | rs10737620 | OR (95% CI) | 1.07 (0.70, 1.63) | 0.84 (0.45, 1.56) | 1.15 (0.71, 1.86) | 1.16 (0.75, 1.81) | 0.82 (0.38, 1.78) | 1.01 (0.55, 1.86) | 0.74 (0.41, 1.33) |
|  |  |  | p-value | 0.7 | 0.6 | 0.6 | 0.5 | 0.6 | 1.0 | 0.3 |
| 515 | TXK | rs11724347 | OR (95% CI) | 1.33 (0.59, 2.98) | 0.84 (0.25, 2.87) | 1.01 (0.37, 2.71) | 0.85 (0.33, 2.14) | 0.38 (0.05, 2.93) | 1.20 (0.35, 4.09) | 1.60 (0.61, 4.23) |
|  |  |  | p-value | 0.5 | 0.8 | 1.0 | 0.7 | 0.4 | 0.8 | 0.3 |
| 516 | UNC84B | rs2072797 | OR (95% CI) | 0.56 (0.25, 1.28) | 1.59 (0.63, 4.02) | 1.02 (0.44, 2.35) | 1.95 (0.97, 3.95) | 0.49 (0.11, 2.22) | 1.48 (0.48, 4.50) | 1.01 (0.40, 2.52) |
|  |  |  | p-value | 0.2 | 0.3 | 1.0 | 0.06 | 0.4 | 0.5 | 1.0 |
| 517 | VEGFA | rs3025000 | OR (95% CI) | 0.69 (0.43, 1.10) | 1.11 (0.61, 2.02) | 0.92 (0.55, 1.53) | 0.95 (0.59, 1.52) | 1.68 (0.83, 3.39) | 1.15 (0.62, 2.14) | 1.53 (0.88, 2.64) |
|  |  |  | p-value | 0.1 | 0.7 | 0.7 | 0.8 | 0.1 | 0.7 | 0.1 |
| 518 | VEGFA | rs3025010 | OR (95% CI) | 0.97 (0.65, 1.44) | 1.07 (0.62, 1.86) | 1.06 (0.67, 1.67) | 1.11 (0.73, 1.69) | 1.20 (0.61, 2.37) | 1.20 (0.68, 2.12) | 0.78 (0.46, 1.33) |
|  |  |  | p-value | 0.9 | 0.8 | 0.8 | 0.6 | 0.6 | 0.5 | 0.4 |
| 519 | VEGFA | rs833068 | OR (95% CI) | 0.83 (0.54, 1.27) | 1.21 (0.68, 2.16) | 0.90 (0.55, 1.46) | 0.94 (0.60, 1.48) | 1.83 (0.91, 3.66) | 1.75 (0.92, 3.32) | 1.57 (0.92, 2.69) |
|  |  |  | p-value | 0.4 | 0.5 | 0.7 | 0.8 | 0.09 | 0.09 | 0.1 |
| 520 | WDR92 | rs13009282 | OR (95% CI) | 0.77 (0.48, 1.22) | 1.54 (0.83, 2.85) | 0.65 (0.37, 1.16) | 1.37 (0.85, 2.19) | 0.61 (0.26, 1.45) | 0.66 (0.36, 1.22) | 0.93 (0.52, 1.65) |
|  |  |  | p-value | 0.3 | 0.2 | 0.1 | 0.2 | 0.3 | 0.2 | 0.8 |
| 521 | ZAP70 | rs2276645 | OR (95% CI) | 1.22 (0.81, 1.82) | 1.19 (0.68, 2.10) | 0.49 (0.28, 0.83) | 0.81 (0.52, 1.26) | 1.49 (0.77, 2.89) | 0.72 (0.42, 1.24) | 1.01 (0.61, 1.69) |
|  |  |  | p-value | 0.3 | 0.5 | 0.008 | 0.3 | 0.2 | 0.2 | 1.0 |
| 522 | ZNF675 | rs11671053 | OR (95% CI) | 1.46 (0.96, 2.22) | 0.94 (0.52, 1.70) | 0.73 (0.45, 1.20) | 0.97 (0.62, 1.51) | 1.06 (0.51, 2.19) | 1.00 (0.55, 1.82) | 0.84 (0.48, 1.46) |
|  |  |  | p-value | 0.08 | 0.8 | 0.2 | 0.9 | 0.9 | 1.0 | 0.5 |
| ^a^Questionable genotype quality, site may have copy number variation  ^a^Questionable genotype quality, site may have copy number variation  ^a^Questionable genotype quality, site may have copy number variation  ^a^Questionable genotype quality, site may have copy number variation  ^a^Questionable genotype quality, site may have copy number variation  ^a^Questionable genotype quality, site may have copy number variation  ^a^Questionable genotype quality, site may have copy number variation  ^a^Questionable genotype quality, site may have copy number variation  ^a^Questionable genotype quality, site may have copy number variation  ^a^Questionable genotype quality, site may have copy number variation  ^a^Questionable genotype quality, site may have copy number variation | | | | | | | | | | |

| **Supplementary Table 4: P-values for sequence kernel association tests (SKAT) of functional pathways and tumor mutation status** | | | | | | | |
| --- | --- | --- | --- | --- | --- | --- | --- |
|  | **KIT exon 11 codon 557-558 deletion** | **KIT exon 11 insertion** | **KIT exon 11 other deletion** | **KIT exon 11 point mutation** | **Other KIT mutation** | **PDGFRA mutation** | **Wild type** |
| AHR and dioxin response^a^ | 0.5/0.5 | 0.7/0.6 | 0.6/0.6 | 0.7/0.7 | 0.01/0.01 | 0.03/0.09 | 0.8/0.8 |
| Alcohol dehydrogenase | 0.4 | 1.0 | 0.8 | 0.6 | 0.1 | 0.9 | 0.3 |
| Anti-apoptosis | 0.7 | 0.3 | 1.0 | 0.7 | 0.7 | 0.6 | 0.6 |
| Apoptotic process | 0.7 | 0.8 | 0.1 | 0.6 | 0.2 | 0.9 | 1.0 |
| Cell proliferation | 0.6 | 0.2 | 0.5 | 1.0 | 0.2 | 0.2 | 0.8 |
| Cell surface receptor signaling pathway | 0.2 | 1.0 | 0.5 | 0.5 | 0.7 | 0.4 | 0.4 |
| Chemokine activity | 1.0 | 0.8 | 0.7 | 1.0 | 0.4 | 0.6 | 0.3 |
| Chemotaxis | 0.6 | 0.3 | 0.7 | 1.0 | 0.2 | 0.7 | 0.7 |
| Cytochrome P450 | 0.6 | 0.1 | 0.9 | 0.5 | 0.1 | 0.2 | 0.8 |
| Cytokine activity^a^ | 0.7/0.7 | 0.5/0.5 | 0.4/0.3 | 0.5/0.5 | 0.2/0.3 | 0.5/0.5 | 0.5/0.6 |
| Cytokine receptor activity | 0.5 | 0.3 | 0.9 | 1.0 | 0.6 | 0.4 | 0.1 |
| Cytokine-mediated signaling pathway | 0.1 | 0.2 | 0.4 | 0.4 | 0.5 | 0.1 | 0.8 |
| Defense response | 0.3 | 0.4 | 0.1 | 0.2 | 0.1 | 0.005 | 0.2 |
| Defense response to bacterium | 0.2 | 0.6 | 0.3 | 0.6 | 0.2 | 0.08 | 0.1 |
| Defense response to virus | 1.0 | 0.2 | 0.9 | 0.5 | 0.2 | 0.4 | 0.2 |
| G-protein coupled receptor signaling pathway | 0.3 | 0.2 | 0.8 | 0.8 | 0.06 | 0.9 | 0.2 |
| Humoral immune response | 0.1 | 0.2 | 0.9 | 0.9 | 0.6 | 0.5 | 0.02 |
| Immune response | 0.4 | 0.3 | 0.3 | 0.2 | 0.3 | 0.1 | 0.4 |
| Induction of apoptosis | 0.5 | 1.0 | 0.5 | 0.8 | 0.2 | 0.05 | 1.0 |
| Inflammatory response | 0.9 | 0.7 | 0.5 | 0.6 | 0.7 | 0.4 | 0.3 |
| Innate immune response | 0.5 | 0.6 | 0.2 | 0.9 | 0.3 | 0.7 | 0.8 |
| Intracellular signal transduction | 0.1 | 0.5 | 0.4 | 0.2 | 0.1 | 0.3 | 0.8 |
| MMPs^a^ | 0.4/0.4 | 0.8/0.7 | 0.8/0.8 | 0.2/0.2 | 0.5/0.4 | 0.5/0.4 | 0.8/0.9 |
| Negative regulation of apoptotic process | 0.1 | 0.4 | 0.03 | 0.5 | 0.2 | 0.4 | 0.8 |
| Negative regulation of cell proliferation | 0.1 | 0.6 | 0.2 | 0.9 | 0.5 | 0.04 | 0.9 |
| Negative regulation of immune response | 0.4 | 0.7 | 0.7 | 0.9 | 0.08 | 0.01 | 1.0 |
| Other toxin metabolism | 0.4 | 0.7 | 0.7 | 0.2 | 0.6 | 0.8 | 0.8 |
| PDGFRA | 0.9 | 1.0 | 0.6 | 0.8 | 0.4 | 0.8 | 0.5 |
| Positive regulation of apoptotic process | 0.4 | 0.8 | 0.9 | 0.8 | 0.5 | 0.7 | 0.4 |
| Positive regulation of cell proliferation^a^ | 0.3/0.3 | 0.7/0.6 | 0.4/0.3 | 0.2/0.2 | 0.2/0.3 | 0.3/0.4 | 0.9/0.9 |
| Positive regulation of immune response | 0.2 | 0.1 | 0.4 | 0.9 | 0.2 | 0.03 | 0.9 |
| Positive regulation of inflammatory response | 1.0 | 0.5 | 0.7 | 0.8 | 0.6 | 0.9 | 0.9 |
| Protein kinase activity | 0.6 | 0.6 | 0.1 | 0.6 | 0.2 | 0.2 | 0.9 |
| Protein phosphorylation | 0.2 | 0.5 | 0.1 | 0.6 | 0.08 | 0.02 | 0.8 |
| Protein tyrosine kinase activity | 0.1 | 0.9 | 0.04 | 0.5 | 0.5 | 0.9 | 1.0 |
| Protein tyrosine phosphatase activity | 0.9 | 0.3 | 0.2 | 0.7 | 0.7 | 0.9 | 0.3 |
| Receptor activity | 0.2 | 0.4 | 0.2 | 0.9 | 0.7 | 0.4 | 0.7 |
| Regulation of apoptotic process | 0.8 | 0.6 | 0.9 | 0.5 | 0.4 | 0.9 | 0.7 |
| Regulation of cell proliferation | 0.3 | 1.0 | 0.7 | 0.5 | 0.5 | 1.0 | 0.6 |
| Regulation of immune response | 1.0 | 0.5 | 0.8 | 0.6 | 0.7 | 0.6 | 0.7 |
| Regulation of receptor activity | 0.4 | 1.0 | 0.5 | 0.5 | 0.4 | 0.9 | 0.8 |
| Response to chemical stimulus | 0.7 | 0.3 | 0.8 | 0.8 | 0.3 | 0.7 | 0.4 |
| Response to hypoxia | 0.5 | 0.8 | 0.06 | 0.7 | 0.2 | 0.2 | 0.8 |
| Response to oxidative stress | 0.3 | 0.9 | 0.2 | 0.4 | 0.5 | 0.7 | 0.1 |
| Response to stress | 0.4 | 0.7 | 0.02 | 0.8 | 0.4 | 0.8 | 1.0 |
| Response to virus | 0.8 | 0.7 | 0.2 | 0.3 | 0.5 | 0.3 | 0.7 |
| Signal transducer activity | 0.4 | 0.2 | 0.1 | 0.8 | 0.7 | 0.6 | 1.0 |
| Signal transduction | 0.5 | 0.2 | 0.3 | 0.9 | 0.1 | 0.7 | 0.4 |
| T cell receptor signaling pathway | 0.5 | 0.7 | 0.08 | 0.06 | 0.6 | 0.07 | 0.7 |
| Transmembrane receptor protein tyrosine phosphatase activity | 0.7 | 0.2 | 0.3 | 0.5 | 0.4 | 0.9 | 0.1 |
| Transmembrane signaling receptor activity | 0.6 | 0.8 | 0.7 | 0.3 | 0.9 | 0.2 | 0.6 |
| ^a^P-values provided for tests including (first value) or excluding (second value) potentially problematic SNPs (rs4947982: AHR/Dioxin; rs6141: cytokine activity and positive regulation of cell proliferation; rs470215: MMPs) | | | | | | | |
